# Supplementary material for: Efineptakin alfa (NT-I7) improves overall survival and induces immune niches in murine tumors
Source: Genes Dis. 2026 Mar 10;14(1):102136. doi: 10.1016/j.gendis.2026.102136 (PMC13583571; doi:10.1016/j.gendis.2026.102136)

Supplementary Figure 1. Effect of NT-I7 treatment on lymphoid tissues, bone marrow, and blood

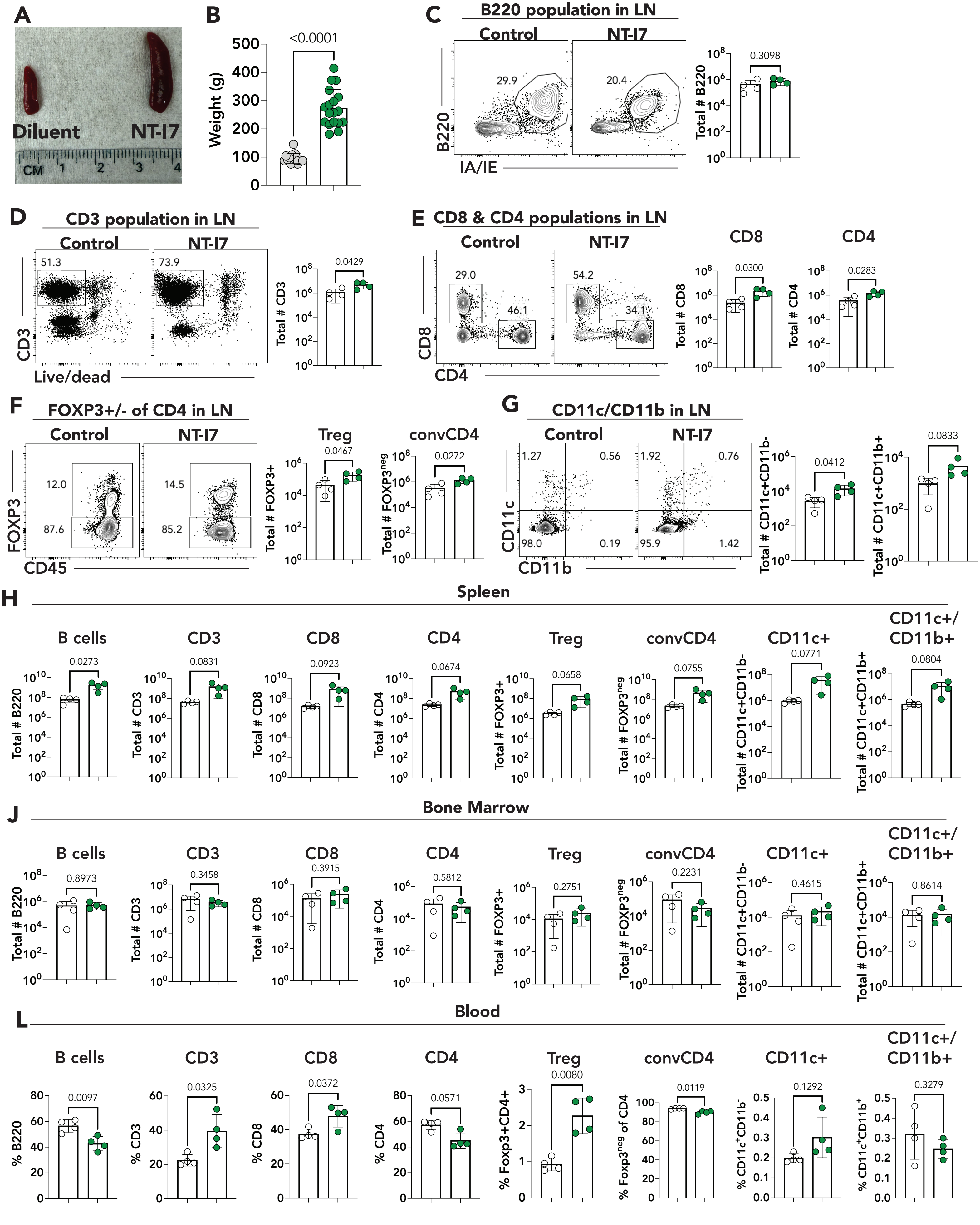

Supplementary Figure 2. Localization of stem-like CD8 T cells in the TME

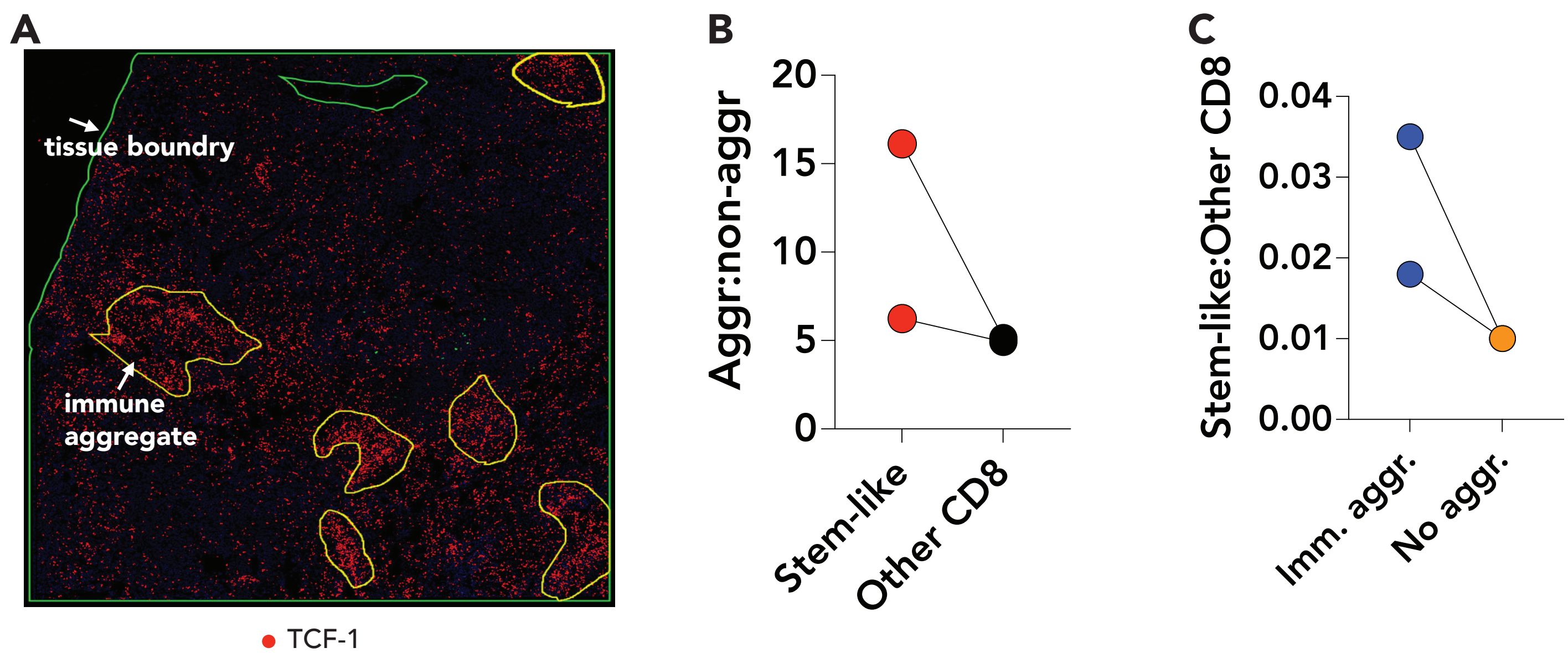

Supplementary Figure 3. Lymphoid structures in lymph node and intraperitoneal CT26 tumors

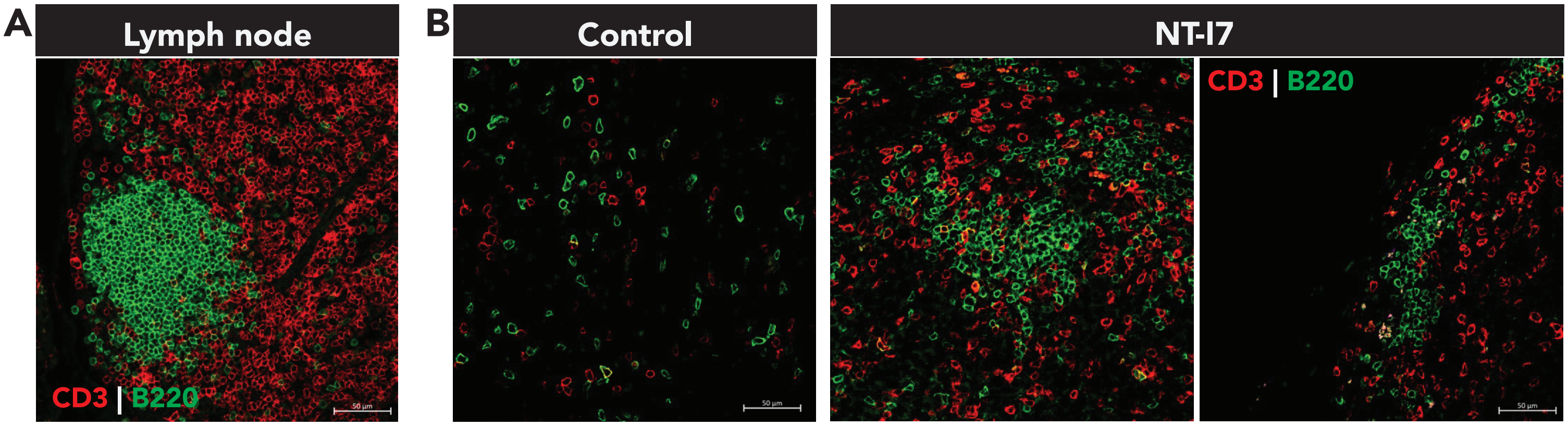

Supplement: Supplementary file 1 [file mmc3.pdf]
