# Supplementary material for: Efineptakin alfa (NT-I7) improves overall survival and induces immune niches in murine tumors
Source: Genes Dis. 2026 Mar 10;14(1):102136. doi: 10.1016/j.gendis.2026.102136 (PMC13583571; doi:10.1016/j.gendis.2026.102136)
Supplement: Supplementary file 2 [file mmc1.docx]

Supplementary table 1: Unsupervised clustering

| p_val | avg_log2FC | pct.1 | pct.2 | p_val_adj | cluster | gene |
| --- | --- | --- | --- | --- | --- | --- |
| 3.42588617839352e-59 | 2.767099 | 0.114 | 0.103 | 1.03E-55 | 0 | Pi16 |
| 1.21185172432553e-57 | 2.515347 | 0.602 | 0.267 | 3.64E-54 | 0 | Adgrf5 |
| 1.56695438328988e-46 | 2.265301 | 0.253 | 0.173 | 4.70E-43 | 0 | Car4 |
| 9.5198326073611e-44 | 2.358455 | 0.346 | 0.206 | 2.86E-40 | 0 | Fmo1 |
| 1.64222753481123e-39 | 3.071503 | 0.446 | 0.246 | 4.93E-36 | 0 | Tspan7 |
| 4.19878774800248e-38 | 2.363204 | 0.438 | 0.256 | 1.26E-34 | 0 | Hopx |
| 2.78803981596929e-37 | 3.963803 | 0.068 | 0.105 | 8.36E-34 | 0 | C1qtnf7 |
| 7.73810334851318e-37 | 2.340915 | 0.56 | 0.289 | 2.32E-33 | 0 | Gsn |
| 5.83712789296582e-36 | 2.690482 | 0.502 | 0.266 | 1.75E-32 | 0 | Ramp2 |
| 4.99622627688483e-35 | 2.791833 | 0.087 | 0.138 | 1.50E-31 | 0 | Megf6 |
| 1.87477238686568e-34 | 3.018738 | 0.143 | 0.179 | 5.62E-31 | 0 | Gpr155 |
| 2.80844307834718e-34 | 2.649139 | 0.525 | 0.277 | 8.43E-31 | 0 | Egfl7 |
| 4.03172144436328e-34 | 3.177989 | 0.093 | 0.169 | 1.21E-30 | 0 | Per3 |
| 2.02202854335547e-32 | 2.478362 | 0.178 | 0.16 | 6.07E-29 | 0 | Ighd |
| 4.75417418259885e-32 | 2.388905 | 0.324 | 0.233 | 1.43E-28 | 0 | Hsd11b1 |
| 1.53621334685232e-31 | 2.309247 | 0.201 | 0.182 | 4.61E-28 | 0 | Pdzd2 |
| 8.44609871065604e-31 | 2.143200 | 0.137 | 0.172 | 2.53E-27 | 0 | Msrb3 |
| 9.59951369952958e-31 | 2.198633 | 0.12 | 0.169 | 2.88E-27 | 0 | Col4a4 |
| 9.94789883390602e-31 | 3.111933 | 0.176 | 0.183 | 2.98E-27 | 0 | Retnlg |
| 5.08220496704564e-30 | 2.230399 | 0.376 | 0.247 | 1.52E-26 | 0 | Ppp1r14a |
| 9.63199938320816e-30 | 2.367554 | 0.191 | 0.179 | 2.89E-26 | 0 | Adrb2 |
| 8.64867677099479e-29 | 2.868925 | 0.162 | 0.18 | 2.59E-25 | 0 | Cd79b |
| 1.04711415441839e-28 | 2.297911 | 0.145 | 0.187 | 3.14E-25 | 0 | Hey1 |
| 7.76138235055136e-28 | 2.818515 | 0.197 | 0.194 | 2.33E-24 | 0 | Scn7a |
| 1.87778865037255e-27 | 2.054226 | 0.16 | 0.176 | 5.63E-24 | 0 | Ccdc85a |
| 5.6742497014289e-27 | 2.358870 | 0.106 | 0.153 | 1.70E-23 | 0 | Satb1 |
| 1.0305227135102e-26 | 2.281329 | 0.172 | 0.197 | 3.09E-23 | 0 | Atp6v0d2 |
| 2.85544182535253e-26 | 2.868754 | 0.236 | 0.233 | 8.57E-23 | 0 | Nkd1 |
| 3.09242785815108e-26 | 2.853963 | 0.347 | 0.26 | 9.28E-23 | 0 | Bcam |
| 5.07481560648328e-26 | 3.312075 | 0.093 | 0.13 | 1.52E-22 | 0 | Abi3bp |
| 8.56269479142025e-26 | 3.422433 | 0.388 | 0.255 | 2.57E-22 | 0 | Acvrl1 |
| 9.02840192272537e-26 | 3.526015 | 0.12 | 0.149 | 2.71E-22 | 0 | Ripor2 |
| 5.02847784837693e-25 | 2.441705 | 0.346 | 0.252 | 1.51E-21 | 0 | Foxf1 |
| 5.39529784683739e-25 | 2.718308 | 0.438 | 0.283 | 1.62E-21 | 0 | Slc43a3 |
| 2.15761455460366e-24 | 2.340014 | 0.1 | 0.191 | 6.47E-21 | 0 | Hcst |
| 6.68280985345831e-24 | 2.108219 | 0.129 | 0.168 | 2.00E-20 | 0 | Npr3 |
| 5.73901408265113e-23 | 2.064520 | 0.232 | 0.25 | 1.72E-19 | 0 | Ednrb |
| 5.79447405566358e-23 | 2.717883 | 0.199 | 0.207 | 1.74E-19 | 0 | Mgll |
| 8.22958207430668e-23 | 2.697071 | 0.181 | 0.209 | 2.47E-19 | 0 | Sell |
| 8.73776607031938e-23 | 2.193025 | 0.145 | 0.186 | 2.62E-19 | 0 | Cish |
| 2.01963018430787e-22 | 3.148433 | 0.156 | 0.18 | 6.06E-19 | 0 | Slc16a9 |
| 5.22918251884697e-22 | 2.362760 | 0.133 | 0.173 | 1.57E-18 | 0 | Npr2 |
| 9.5925159267621e-22 | 2.498836 | 0.168 | 0.216 | 2.88E-18 | 0 | Plcb4 |
| 1.19987095896342e-21 | 2.019702 | 0.156 | 0.193 | 3.60E-18 | 0 | Cpm |
| 3.63824701713783e-21 | 2.939505 | 0.241 | 0.237 | 1.09E-17 | 0 | Tent5c |
| 4.8369243089955e-21 | 2.307412 | 0.469 | 0.309 | 1.45E-17 | 0 | Rnase4 |
| 5.6176868433464e-21 | 2.457953 | 0.098 | 0.151 | 1.69E-17 | 0 | Samd12 |
| 5.97493828732896e-21 | 3.200453 | 0.145 | 0.196 | 1.79E-17 | 0 | Mal2 |
| 6.3939016488939e-21 | 2.496258 | 0.174 | 0.189 | 1.92E-17 | 0 | Akap5 |
| 7.12876950037636e-21 | 3.400356 | 0.241 | 0.248 | 2.14E-17 | 0 | Irx1 |
| 1.59272870591469e-20 | 3.524172 | 0.12 | 0.177 | 4.78E-17 | 0 | Nova2 |
| 1.75265272884434e-20 | 3.342273 | 0.145 | 0.171 | 5.26E-17 | 0 | Reck |
| 2.36827116984081e-20 | 2.221439 | 0.137 | 0.215 | 7.10E-17 | 0 | Itpr2 |
| 4.43848335752982e-20 | 2.925869 | 0.199 | 0.241 | 1.33E-16 | 0 | Gadd45b |
| 9.05816368907167e-20 | 3.057621 | 0.307 | 0.272 | 2.72E-16 | 0 | Alas2 |
| 2.62442268151404e-19 | 3.324619 | 0.292 | 0.257 | 7.87E-16 | 0 | Arhgef3 |
| 3.13868226080306e-19 | 2.607911 | 0.124 | 0.19 | 9.42E-16 | 0 | Gbp9 |
| 4.04360641675228e-19 | 2.693313 | 0.106 | 0.173 | 1.21E-15 | 0 | Tie1 |
| 4.22057532957221e-19 | 3.188258 | 0.1 | 0.175 | 1.27E-15 | 0 | Mturn |
| 5.53306247771587e-19 | 2.125865 | 0.12 | 0.173 | 1.66E-15 | 0 | Cyfip2 |
| 6.60249809082652e-19 | 2.365677 | 0.147 | 0.222 | 1.98E-15 | 0 | Mmp2 |
| 8.82558564397086e-19 | 2.929912 | 0.28 | 0.256 | 2.65E-15 | 0 | Prex2 |
| 1.1380417457381e-18 | 2.620395 | 0.313 | 0.269 | 3.41E-15 | 0 | Lamp3 |
| 3.04366051399473e-18 | 2.919569 | 0.266 | 0.251 | 9.13E-15 | 0 | Spock2 |
| 4.03937643488503e-18 | 3.421986 | 0.253 | 0.244 | 1.21E-14 | 0 | Tek |
| 5.05384391738216e-18 | 3.724302 | 0.139 | 0.216 | 1.52E-14 | 0 | Klhl6 |
| 5.96580966515715e-18 | 2.984297 | 0.068 | 0.141 | 1.79E-14 | 0 | Pdgfd |
| 1.03730602281791e-17 | 2.637866 | 0.145 | 0.21 | 3.11E-14 | 0 | Pag1 |
| 1.27536070669054e-17 | 2.722184 | 0.212 | 0.241 | 3.83E-14 | 0 | Hc |
| 1.42116678743216e-17 | 2.283094 | 0.104 | 0.185 | 4.26E-14 | 0 | Clip4 |
| 1.4629428212767e-17 | 2.077730 | 0.133 | 0.189 | 4.39E-14 | 0 | Limch1 |
| 6.30524820850833e-17 | 2.197338 | 0.307 | 0.273 | 1.89E-13 | 0 | Cmklr1 |
| 9.1857912833347e-17 | 2.145431 | 0.077 | 0.167 | 2.76E-13 | 0 | Lsr |
| 1.06408263698471e-16 | 2.522610 | 0.388 | 0.295 | 3.19E-13 | 0 | Pde4b |
| 1.30222953682639e-16 | 2.714117 | 0.102 | 0.189 | 3.91E-13 | 0 | Fgf2 |
| 1.63228960216808e-16 | 2.352589 | 0.135 | 0.195 | 4.90E-13 | 0 | Tbx4 |
| 1.72712475554838e-16 | 2.270152 | 0.068 | 0.182 | 5.18E-13 | 0 | Pcdhgb4 |
| 1.88530094675461e-16 | 2.209885 | 0.241 | 0.261 | 5.66E-13 | 0 | H2-Q6 |
| 2.26249975019822e-16 | 3.901338 | 0.087 | 0.183 | 6.79E-13 | 0 | Tbc1d8 |
| 4.10802582285667e-16 | 3.609336 | 0.168 | 0.224 | 1.23E-12 | 0 | Rnf144a |
| 5.83301750245444e-16 | 2.150074 | 0.116 | 0.225 | 1.75E-12 | 0 | Kctd6 |
| 7.2701382566892e-16 | 3.262100 | 0.145 | 0.22 | 2.18E-12 | 0 | Mcemp1 |
| 8.81439129115067e-16 | 3.342783 | 0.201 | 0.236 | 2.64E-12 | 0 | Shroom3 |
| 1.45112645512211e-15 | 2.957489 | 0.181 | 0.411 | 4.35E-12 | 0 | Rnf126 |
| 1.66189633030091e-15 | 2.780398 | 0.409 | 0.289 | 4.99E-12 | 0 | Emp2 |
| 1.08968048182362e-14 | 2.751373 | 0.114 | 0.224 | 3.27E-11 | 0 | Arrdc2 |
| 1.55668282359338e-14 | 2.415305 | 0.129 | 0.209 | 4.67E-11 | 0 | Wtip |
| 1.60782239454263e-14 | 3.186974 | 0.234 | 0.252 | 4.82E-11 | 0 | Sfta2 |
| 1.64725833373242e-14 | 2.073677 | 0.164 | 0.234 | 4.94E-11 | 0 | Pcx |
| 3.18152044753267e-14 | 3.085291 | 0.139 | 0.213 | 9.54E-11 | 0 | H2-DMb2 |
| 4.43032530663996e-14 | 2.210899 | 0.108 | 0.185 | 1.33E-10 | 0 | Tbx3 |
| 5.35080364885314e-14 | 4.068576 | 0.112 | 0.201 | 1.61E-10 | 0 | Uba7 |
| 9.41696436445281e-14 | 2.795968 | 0.305 | 0.299 | 2.83E-10 | 0 | Rgs12 |
| 1.17330832854558e-13 | 2.089680 | 0.158 | 0.238 | 3.52E-10 | 0 | Gimap4 |
| 2.08437791573985e-13 | 2.124421 | 0.153 | 0.229 | 6.25E-10 | 0 | Mfng |
| 2.54227722563494e-13 | 2.875990 | 0.208 | 0.253 | 7.63E-10 | 0 | Lifr |
| 3.1597193355795e-13 | 2.337601 | 0.097 | 0.193 | 9.48E-10 | 0 | Arap2 |
| 4.25591533917342e-13 | 2.312004 | 0.133 | 0.226 | 1.28E-09 | 0 | Itgal |
| 5.0030841222104e-13 | 2.936025 | 0.098 | 0.197 | 1.50E-09 | 0 | Per2 |
| 5.3062966304979e-13 | 3.234339 | 0.154 | 0.227 | 1.59E-09 | 0 | Zfp362 |
| 6.15339523819412e-13 | 3.564050 | 0.149 | 0.217 | 1.85E-09 | 0 | Gja4 |
| 6.76987874086399e-13 | 2.617451 | 0.232 | 0.274 | 2.03E-09 | 0 | Add3 |
| 1.34893644986203e-12 | 2.644587 | 0.276 | 0.281 | 4.05E-09 | 0 | Rapgef5 |
| 1.37151938862355e-12 | 3.128203 | 0.127 | 0.237 | 4.11E-09 | 0 | Bcar3 |
| 1.65556548799404e-12 | 2.714039 | 0.378 | 0.314 | 4.97E-09 | 0 | Tsc22d3 |
| 1.83482958352611e-12 | 2.107971 | 0.322 | 0.29 | 5.50E-09 | 0 | Pecam1 |
| 2.6084496391981e-12 | 2.262276 | 0.127 | 0.252 | 7.83E-09 | 0 | Htra3 |
| 3.39255340104934e-12 | 2.254029 | 0.108 | 0.215 | 1.02E-08 | 0 | Lamc2 |
| 4.1344088607148e-12 | 2.390635 | 0.205 | 0.25 | 1.24E-08 | 0 | Sema3f |
| 4.76274186448997e-12 | 3.333083 | 0.11 | 0.23 | 1.43E-08 | 0 | Fam117a |
| 5.33883309674026e-12 | 2.482307 | 0.207 | 0.261 | 1.60E-08 | 0 | Dennd3 |
| 8.34531923644379e-12 | 3.647284 | 0.228 | 0.28 | 2.50E-08 | 0 | Arhgap29 |
| 9.26110398176052e-12 | 2.243025 | 0.241 | 0.295 | 2.78E-08 | 0 | Cd37 |
| 1.04023228973475e-11 | 3.237880 | 0.154 | 0.24 | 3.12E-08 | 0 | Adamts2 |
| 1.13727755207802e-11 | 2.204436 | 0.16 | 0.231 | 3.41E-08 | 0 | Fgf1 |
| 1.41684131557259e-11 | 3.566502 | 0.112 | 0.232 | 4.25E-08 | 0 | Vsir |
| 1.55452071827923e-11 | 3.194753 | 0.187 | 0.251 | 4.66E-08 | 0 | Slfn5 |
| 1.59965314670669e-11 | 2.829567 | 0.16 | 0.226 | 4.80E-08 | 0 | Cytip |
| 2.02989594474825e-11 | 2.614042 | 0.203 | 0.253 | 6.09E-08 | 0 | Klf4 |
| 2.24678946312964e-11 | 2.729728 | 0.168 | 0.249 | 6.74E-08 | 0 | She |
| 3.32440376733854e-11 | 5.131412 | 0.236 | 0.284 | 9.97E-08 | 0 | Map3k6 |
| 3.3410428687699e-11 | 2.714606 | 0.22 | 0.272 | 1.00E-07 | 0 | Arrb1 |
| 4.16656058928986e-11 | 2.410901 | 0.392 | 0.295 | 1.25E-07 | 0 | Slc9a3r2 |
| 4.74925725556418e-11 | 2.560527 | 0.164 | 0.249 | 1.42E-07 | 0 | Marco |
| 5.19979500009907e-11 | 2.200578 | 0.234 | 0.275 | 1.56E-07 | 0 | Cystm1 |
| 6.02778617093088e-11 | 2.151067 | 0.347 | 0.301 | 1.81E-07 | 0 | F11r |
| 8.36194882298992e-11 | 2.734419 | 0.168 | 0.244 | 2.51E-07 | 0 | Abi3 |
| 8.53217603102225e-11 | 3.180596 | 0.278 | 0.289 | 2.56E-07 | 0 | Arhgap31 |
| 9.29395937525686e-11 | 2.570135 | 0.122 | 0.237 | 2.79E-07 | 0 | Tnfaip8l2 |
| 1.0234280205596e-10 | 2.363953 | 0.133 | 0.214 | 3.07E-07 | 0 | Gstt1 |
| 1.04235328794089e-10 | 3.834779 | 0.12 | 0.201 | 3.13E-07 | 0 | Il16 |
| 1.0639612748216e-10 | 2.183335 | 0.535 | 0.378 | 3.19E-07 | 0 | Serinc3 |
| 1.15191984123214e-10 | 2.497105 | 0.332 | 0.309 | 3.46E-07 | 0 | Ptprc |
| 1.24208683391617e-10 | 3.469747 | 0.066 | 0.194 | 3.73E-07 | 0 | Cyp27a1 |
| 1.24223025730913e-10 | 2.183732 | 0.112 | 0.22 | 3.73E-07 | 0 | Trim30a |
| 1.414963014848e-10 | 3.423047 | 0.183 | 0.275 | 4.24E-07 | 0 | Phkg2 |
| 2.20243156282903e-10 | 2.465047 | 0.197 | 0.26 | 6.61E-07 | 0 | Gimap6 |
| 2.35069856146837e-10 | 3.495471 | 0.124 | 0.223 | 7.05E-07 | 0 | Plxna2 |
| 2.78009936092999e-10 | 2.161526 | 0.151 | 0.23 | 8.34E-07 | 0 | Nod1 |
| 2.91427781659927e-10 | 3.161987 | 0.1 | 0.207 | 8.74E-07 | 0 | Grap |
| 2.99014115170271e-10 | 2.674694 | 0.141 | 0.237 | 8.97E-07 | 0 | Pygl |
| 3.06134551748232e-10 | 3.501568 | 0.129 | 0.21 | 9.18E-07 | 0 | Myct1 |
| 3.50366375333991e-10 | 3.798860 | 0.106 | 0.201 | 1.05E-06 | 0 | Ang |
| 3.68383574191041e-10 | 2.796117 | 0.212 | 0.274 | 1.11E-06 | 0 | Dock6 |
| 4.67407184824487e-10 | 4.045438 | 0.11 | 0.233 | 1.40E-06 | 0 | Dennd4a |
| 4.98022732391945e-10 | 4.363899 | 0.131 | 0.225 | 1.49E-06 | 0 | Stard8 |
| 6.81207834012433e-10 | 2.966490 | 0.133 | 0.231 | 2.04E-06 | 0 | Fst |
| 7.00130423827289e-10 | 2.840834 | 0.33 | 0.309 | 2.10E-06 | 0 | Creg1 |
| 8.52689645027571e-10 | 3.543105 | 0.212 | 0.27 | 2.56E-06 | 0 | Sept4 |
| 9.82463140409848e-10 | 3.446341 | 0.261 | 0.28 | 2.95E-06 | 0 | Cyp2d22 |
| 1.03829850065912e-09 | 3.093162 | 0.295 | 0.287 | 3.11E-06 | 0 | Esam |
| 1.19955535085784e-09 | 2.421416 | 0.257 | 0.26 | 3.60E-06 | 0 | Jcad |
| 1.45474127226e-09 | 2.428176 | 0.297 | 0.286 | 4.36E-06 | 0 | Pltp |
| 1.86263200631121e-09 | 2.668392 | 0.21 | 0.284 | 5.59E-06 | 0 | Antxr1 |
| 1.92447854413548e-09 | 2.304272 | 0.351 | 0.301 | 5.77E-06 | 0 | Qsox1 |
| 2.3212468549019e-09 | 3.358594 | 0.218 | 0.271 | 6.96E-06 | 0 | Afap1l1 |
| 2.51611084437738e-09 | 3.892987 | 0.125 | 0.221 | 7.55E-06 | 0 | Vldlr |
| 2.55530673758749e-09 | 3.101892 | 0.278 | 0.293 | 7.67E-06 | 0 | Trp53inp1 |
| 3.42905404911499e-09 | 2.045547 | 0.172 | 0.271 | 1.03E-05 | 0 | Fgr |
| 3.61953934157231e-09 | 2.540991 | 0.264 | 0.276 | 1.09E-05 | 0 | Tmcc2 |
| 3.65240210926685e-09 | 3.555891 | 0.27 | 0.275 | 1.10E-05 | 0 | Pmp22 |
| 4.2481362167469e-09 | 2.888528 | 0.11 | 0.209 | 1.27E-05 | 0 | Abcb1a |
| 5.36262925789135e-09 | 2.279486 | 0.178 | 0.253 | 1.61E-05 | 0 | Erg |
| 6.05954284320291e-09 | 2.256824 | 0.261 | 0.284 | 1.82E-05 | 0 | Itga1 |
| 6.95601983744993e-09 | 2.081585 | 0.261 | 0.423 | 2.09E-05 | 0 | Ndst1 |
| 8.0577508972401e-09 | 3.311369 | 0.108 | 0.248 | 2.42E-05 | 0 | Rapgef2 |
| 1.49161762362507e-08 | 2.556036 | 0.299 | 0.305 | 4.47E-05 | 0 | Bmpr2 |
| 1.71723204036874e-08 | 2.357835 | 0.313 | 0.327 | 5.15E-05 | 0 | H2-K1 |
| 1.99760755337877e-08 | 3.450165 | 0.147 | 0.269 | 5.99E-05 | 0 | Plaat3 |
| 3.71499577732018e-08 | 3.339573 | 0.241 | 0.285 | 1.11E-04 | 0 | Selplg |
| 3.7398583392698e-08 | 3.222439 | 0.093 | 0.198 | 1.12E-04 | 0 | C1ra |
| 3.84934731179703e-08 | 2.196293 | 0.133 | 0.238 | 1.15E-04 | 0 | Ntn4 |
| 4.54883069332891e-08 | 2.899006 | 0.249 | 0.285 | 1.36E-04 | 0 | Gprc5a |
| 4.71557243284321e-08 | 2.557079 | 0.1 | 0.229 | 1.41E-04 | 0 | Zhx3 |
| 5.45075529419077e-08 | 2.041891 | 0.286 | 0.401 | 1.64E-04 | 0 | C1qa |
| 5.54541171340648e-08 | 2.455628 | 0.307 | 0.302 | 1.66E-04 | 0 | Snrk |
| 6.37967675228293e-08 | 3.339612 | 0.237 | 0.3 | 1.91E-04 | 0 | Ankrd37 |
| 8.30793849293791e-08 | 2.239112 | 0.12 | 0.23 | 2.49E-04 | 0 | Ptprk |
| 1.07310601295593e-07 | 2.968314 | 0.102 | 0.214 | 3.22E-04 | 0 | Slit2 |
| 1.12201407563803e-07 | 3.824635 | 0.151 | 0.374 | 3.37E-04 | 0 | Hspb11 |
| 1.16186065216327e-07 | 2.191945 | 0.166 | 0.395 | 3.49E-04 | 0 | Memo1 |
| 1.27822979295808e-07 | 3.534701 | 0.131 | 0.25 | 3.83E-04 | 0 | Mxd1 |
| 1.38703350360733e-07 | 5.115714 | 0.208 | 0.283 | 4.16E-04 | 0 | Gngt2 |
| 1.46416418571408e-07 | 2.384124 | 0.106 | 0.217 | 4.39E-04 | 0 | Casp4 |
| 1.58053380200714e-07 | 2.431646 | 0.12 | 0.26 | 4.74E-04 | 0 | Pradc1 |
| 1.82559593907487e-07 | 3.194801 | 0.145 | 0.236 | 5.48E-04 | 0 | Adgrl3 |
| 1.97052537987402e-07 | 2.256991 | 0.208 | 0.296 | 5.91E-04 | 0 | Cpeb4 |
| 2.1223440557326e-07 | 2.670004 | 0.239 | 0.323 | 6.37E-04 | 0 | Slc9a6 |
| 2.16635877693759e-07 | 2.460386 | 0.108 | 0.223 | 6.50E-04 | 0 | Ldb2 |
| 2.19488233733646e-07 | 2.305440 | 0.147 | 0.266 | 6.58E-04 | 0 | Ephb4 |
| 2.20377952033346e-07 | 2.348060 | 0.203 | 0.399 | 6.61E-04 | 0 | Pfkl |
| 2.23340889095683e-07 | 4.333486 | 0.178 | 0.278 | 6.70E-04 | 0 | Dgka |
| 2.33052565911355e-07 | 3.053727 | 0.143 | 0.241 | 6.99E-04 | 0 | Hint2 |
| 2.35648552867723e-07 | 3.647213 | 0.127 | 0.247 | 7.07E-04 | 0 | Xaf1 |
| 2.47587486946041e-07 | 2.237819 | 0.197 | 0.292 | 7.43E-04 | 0 | Tmem140 |
| 3.31026815260495e-07 | 2.353585 | 0.153 | 0.267 | 9.93E-04 | 0 | Pdgfrb |
| 3.37255502014334e-07 | 2.917418 | 0.075 | 0.224 | 1.01E-03 | 0 | Ctif |
| 3.69348902350967e-07 | 2.393778 | 0.162 | 0.385 | 1.11E-03 | 0 | Sh3bgrl2 |
| 4.32159229622532e-07 | 2.019412 | 0.091 | 0.219 | 1.30E-03 | 0 | Lpin1 |
| 4.48085834655436e-07 | 4.335791 | 0.247 | 0.402 | 1.34E-03 | 0 | Plekho2 |
| 4.79410523129019e-07 | 3.326103 | 0.118 | 0.244 | 1.44E-03 | 0 | Rffl |
| 5.05814365422327e-07 | 4.298005 | 0.116 | 0.254 | 1.52E-03 | 0 | Tnfsf13 |
| 5.19062597250594e-07 | 2.499199 | 0.104 | 0.234 | 1.56E-03 | 0 | Akap7 |
| 5.56368457866696e-07 | 4.019285 | 0.176 | 0.277 | 1.67E-03 | 0 | Stx3 |
| 5.72242931910012e-07 | 2.580326 | 0.112 | 0.264 | 1.72E-03 | 0 | Ppp1r37 |
| 6.05476907167224e-07 | 2.173662 | 0.193 | 0.284 | 1.82E-03 | 0 | Tgfb1i1 |
| 7.44191452958625e-07 | 2.694872 | 0.151 | 0.261 | 2.23E-03 | 0 | Map3k5 |
| 7.8479800248838e-07 | 2.026766 | 0.207 | 0.275 | 2.35E-03 | 0 | Cryab |
| 1.00878707906553e-06 | 2.841903 | 0.17 | 0.28 | 3.03E-03 | 0 | Nfam1 |
| 1.34405478930996e-06 | 2.351966 | 0.18 | 0.273 | 4.03E-03 | 0 | Ttyh3 |
| 1.37726668566123e-06 | 3.863530 | 0.102 | 0.238 | 4.13E-03 | 0 | Ifi207 |
| 1.38480780579837e-06 | 3.232909 | 0.154 | 0.248 | 4.15E-03 | 0 | Mcc |
| 2.45443344769499e-06 | 4.518885 | 0.154 | 0.265 | 7.36E-03 | 0 | Il2rg |
| 3.04144501863392e-06 | 3.414501 | 0.124 | 0.251 | 9.12E-03 | 0 | Lrch1 |
| 3.42336851506773e-06 | 2.138779 | 0.18 | 0.293 | 1.03E-02 | 0 | Slc15a3 |
| 3.94758737267902e-06 | 2.142686 | 0.143 | 0.277 | 1.18E-02 | 0 | Tnfrsf1b |
| 3.95292556300231e-06 | 2.334653 | 0.224 | 0.288 | 1.19E-02 | 0 | Cd302 |
| 4.02431053394633e-06 | 2.319252 | 0.114 | 0.252 | 1.21E-02 | 0 | Nav2 |
| 4.09071591461289e-06 | 3.772356 | 0.11 | 0.229 | 1.23E-02 | 0 | Sox18 |
| 4.70665463857028e-06 | 2.683202 | 0.421 | 0.341 | 1.41E-02 | 0 | Cd93 |
| 5.97231053979037e-06 | 3.469410 | 0.195 | 0.288 | 1.79E-02 | 0 | H2-DMa |
| 6.17887898697376e-06 | 2.186627 | 0.168 | 0.369 | 1.85E-02 | 0 | Vps13b |
| 6.73119262783692e-06 | 2.219392 | 0.156 | 0.266 | 2.02E-02 | 0 | Notch4 |
| 7.52844080203587e-06 | 4.096302 | 0.114 | 0.245 | 2.26E-02 | 0 | Bin2 |
| 9.52514153460354e-06 | 2.061461 | 0.093 | 0.244 | 2.86E-02 | 0 | Myo6 |
| 9.85420566826142e-06 | 2.938869 | 0.104 | 0.257 | 2.96E-02 | 0 | Ephb3 |
| 9.89910732603557e-06 | 2.445503 | 0.185 | 0.281 | 2.97E-02 | 0 | Arhgap27 |
| 1.21288024608305e-05 | 2.270869 | 0.172 | 0.283 | 3.64E-02 | 0 | Slc30a1 |
| 1.27830757355741e-05 | 3.324605 | 0.095 | 0.228 | 3.83E-02 | 0 | Ppp1r13b |
| 1.32803063683951e-05 | 4.292687 | 0.338 | 0.328 | 3.98E-02 | 0 | Epb41 |
| 1.44754809587926e-05 | 4.365255 | 0.23 | 0.296 | 4.34E-02 | 0 | Flt1 |
| 1.54089243286205e-236 | 3.417635 | 0.99 | 0.247 | 4.62E-233 | 1 | Epas1 |
| 6.02792291772395e-210 | 2.642143 | 0.99 | 0.319 | 1.81E-206 | 1 | Hba-a2 |
| 8.35526755064101e-210 | 2.747507 | 0.978 | 0.298 | 2.51E-206 | 1 | Hbb-bs |
| 8.02287415885123e-206 | 2.467986 | 0.996 | 0.287 | 2.41E-202 | 1 | Sftpc |
| 1.14592097629602e-189 | 2.070231 | 0.96 | 0.246 | 3.44E-186 | 1 | Inmt |
| 6.45596951174287e-189 | 2.818004 | 0.929 | 0.225 | 1.94E-185 | 1 | Ace |
| 5.16939527413551e-176 | 3.153479 | 0.923 | 0.248 | 1.55E-172 | 1 | Tns1 |
| 6.6578116483841e-172 | 3.079906 | 0.883 | 0.239 | 2.00E-168 | 1 | S100a9 |
| 7.24590531406415e-170 | 2.085894 | 0.921 | 0.262 | 2.17E-166 | 1 | Gpx3 |
| 4.02874077702401e-169 | 4.076889 | 0.855 | 0.213 | 1.21E-165 | 1 | Slco2a1 |
| 1.44957337400471e-168 | 3.742802 | 0.885 | 0.243 | 4.35E-165 | 1 | Calcrl |
| 6.58927843174113e-144 | 3.039950 | 0.804 | 0.207 | 1.98E-140 | 1 | S100a8 |
| 2.15538673006476e-139 | 2.953989 | 0.812 | 0.23 | 6.47E-136 | 1 | Ramp2 |
| 1.77748871209574e-132 | 2.165304 | 0.776 | 0.214 | 5.33E-129 | 1 | Clec14a |
| 7.60605846346132e-125 | 2.886747 | 0.8 | 0.23 | 2.28E-121 | 1 | Ptprb |
| 6.16066576397297e-124 | 3.525642 | 0.744 | 0.171 | 1.85E-120 | 1 | Tmem100 |
| 2.18471670462445e-123 | 2.464102 | 0.792 | 0.246 | 6.55E-120 | 1 | Egfl7 |
| 4.2853987354854e-118 | 2.493494 | 0.819 | 0.281 | 1.29E-114 | 1 | App |
| 5.14973219450444e-118 | 3.410676 | 0.817 | 0.28 | 1.54E-114 | 1 | Cd36 |
| 1.96928645833927e-117 | 3.850482 | 0.792 | 0.269 | 5.91E-114 | 1 | Cdh5 |
| 1.32176217713722e-110 | 2.366502 | 0.732 | 0.197 | 3.97E-107 | 1 | Clic5 |
| 3.45037422309591e-110 | 3.629651 | 0.679 | 0.158 | 1.04E-106 | 1 | Hpgd |
| 7.81134277213792e-107 | 2.755019 | 0.796 | 0.29 | 2.34E-103 | 1 | Sptbn1 |
| 1.02868284925142e-106 | 3.209901 | 0.784 | 0.274 | 3.09E-103 | 1 | Ucp2 |
| 2.84674538834762e-98 | 2.726906 | 0.667 | 0.188 | 8.54E-95 | 1 | Cdo1 |
| 2.68324078443853e-92 | 2.326876 | 0.75 | 0.286 | 8.05E-89 | 1 | Hbb-bt |
| 1.35087162256651e-89 | 2.757021 | 0.641 | 0.183 | 4.05E-86 | 1 | Krt80 |
| 3.87599103873899e-87 | 2.801580 | 0.659 | 0.172 | 1.16E-83 | 1 | Sema3c |
| 9.10775145218412e-86 | 2.676803 | 0.667 | 0.198 | 2.73E-82 | 1 | Plpp3 |
| 9.96263496927863e-86 | 3.702110 | 0.708 | 0.233 | 2.99E-82 | 1 | Adgre5 |
| 1.56000242490361e-80 | 3.737578 | 0.671 | 0.244 | 4.68E-77 | 1 | Jup |
| 5.97818611453238e-77 | 2.875169 | 0.696 | 0.287 | 1.79E-73 | 1 | Xdh |
| 3.51230619709815e-70 | 2.915153 | 0.562 | 0.136 | 1.05E-66 | 1 | Car4 |
| 1.16232223937412e-68 | 2.472201 | 0.599 | 0.176 | 3.49E-65 | 1 | Fmo1 |
| 1.20584127384896e-68 | 2.349252 | 0.639 | 0.225 | 3.62E-65 | 1 | Lpl |
| 4.46427266429686e-68 | 2.780121 | 0.653 | 0.264 | 1.34E-64 | 1 | Pde4b |
| 6.19486064519959e-68 | 2.104569 | 0.669 | 0.241 | 1.86E-64 | 1 | Mfap4 |
| 1.49788574708504e-67 | 2.073760 | 0.583 | 0.163 | 4.49E-64 | 1 | Dpep1 |
| 2.91400058541965e-65 | 3.775740 | 0.575 | 0.171 | 8.74E-62 | 1 | Lims2 |
| 8.50588608174057e-65 | 3.721866 | 0.633 | 0.23 | 2.55E-61 | 1 | Acer2 |
| 1.16184020688291e-64 | 3.381744 | 0.694 | 0.302 | 3.49E-61 | 1 | Klf2 |
| 1.57922324464784e-64 | 3.095526 | 0.569 | 0.178 | 4.74E-61 | 1 | Atp2a3 |
| 2.31801396232046e-62 | 3.271347 | 0.651 | 0.264 | 6.95E-59 | 1 | Slc9a3r2 |
| 9.90848895643037e-62 | 3.770076 | 0.645 | 0.26 | 2.97E-58 | 1 | Sema7a |
| 9.88307571595715e-61 | 2.292309 | 0.583 | 0.2 | 2.96E-57 | 1 | Spon1 |
| 3.21340526486802e-60 | 3.776268 | 0.613 | 0.223 | 9.64E-57 | 1 | Podxl |
| 8.00529902335272e-59 | 3.302516 | 0.599 | 0.222 | 2.40E-55 | 1 | Foxf1 |
| 3.54672967075707e-57 | 3.907347 | 0.595 | 0.23 | 1.06E-53 | 1 | BC028528 |
| 1.38272771327632e-53 | 2.022464 | 0.671 | 0.318 | 4.15E-50 | 1 | Vamp8 |
| 1.5909140289156e-53 | 5.080217 | 0.575 | 0.228 | 4.77E-50 | 1 | Galnt18 |
| 2.07607554718502e-53 | 2.257856 | 0.601 | 0.23 | 6.23E-50 | 1 | Bcam |
| 1.67459228103934e-51 | 2.330364 | 0.581 | 0.205 | 5.02E-48 | 1 | C7 |
| 6.69414225905505e-51 | 2.801836 | 0.603 | 0.269 | 2.01E-47 | 1 | Bmpr2 |
| 1.1564490932379e-50 | 2.630982 | 0.544 | 0.199 | 3.47E-47 | 1 | Csf3r |
| 1.27737952188594e-50 | 2.901248 | 0.605 | 0.257 | 3.83E-47 | 1 | S1pr1 |
| 1.60241774626235e-50 | 3.379846 | 0.552 | 0.194 | 4.81E-47 | 1 | Fas |
| 2.6162647113862e-50 | 3.172525 | 0.534 | 0.157 | 7.85E-47 | 1 | Cox4i2 |
| 3.20158779565083e-50 | 2.719327 | 0.645 | 0.31 | 9.60E-47 | 1 | Tacc1 |
| 5.624571453243e-50 | 2.186751 | 0.581 | 0.253 | 1.69E-46 | 1 | Esam |
| 6.98542904633673e-50 | 3.520009 | 0.575 | 0.237 | 2.10E-46 | 1 | Adgrl4 |
| 7.93287132095969e-50 | 3.322025 | 0.552 | 0.198 | 2.38E-46 | 1 | Sema3g |
| 1.68257208061582e-48 | 3.641547 | 0.637 | 0.255 | 5.05E-45 | 1 | Cldn5 |
| 4.97623782787834e-48 | 3.061184 | 0.512 | 0.137 | 1.49E-44 | 1 | Fgfr3 |
| 6.58230445060932e-48 | 3.473182 | 0.579 | 0.219 | 1.97E-44 | 1 | Cyyr1 |
| 1.28241834270966e-47 | 3.130478 | 0.581 | 0.213 | 3.85E-44 | 1 | Thbd |
| 1.48408389863842e-47 | 2.163024 | 0.623 | 0.291 | 4.45E-44 | 1 | Rnase4 |
| 1.18095460977773e-46 | 2.391153 | 0.587 | 0.222 | 3.54E-43 | 1 | Ppp1r14a |
| 1.47757123531913e-46 | 2.483591 | 0.532 | 0.226 | 4.43E-43 | 1 | H2-Q6 |
| 4.76280635222505e-45 | 2.708234 | 0.53 | 0.225 | 1.43E-41 | 1 | Fgd5 |
| 9.9381420750969e-44 | 3.505815 | 0.538 | 0.236 | 2.98E-40 | 1 | Sh2d3c |
| 1.51295499636841e-43 | 2.751766 | 0.569 | 0.22 | 4.54E-40 | 1 | Fhl1 |
| 1.53455725522564e-43 | 2.070746 | 0.639 | 0.315 | 4.60E-40 | 1 | Cd93 |
| 1.6628796206851e-43 | 2.463510 | 0.506 | 0.2 | 4.99E-40 | 1 | Nkd1 |
| 3.73222286379458e-43 | 2.600747 | 0.577 | 0.255 | 1.12E-39 | 1 | Tcn2 |
| 1.05551522498017e-42 | 2.896176 | 0.599 | 0.274 | 3.17E-39 | 1 | Ets2 |
| 9.03017449175663e-42 | 3.814947 | 0.593 | 0.265 | 2.71E-38 | 1 | Gpihbp1 |
| 1.53513362931013e-41 | 3.373770 | 0.589 | 0.258 | 4.61E-38 | 1 | Pecam1 |
| 5.84903269452217e-41 | 2.134205 | 0.651 | 0.33 | 1.75E-37 | 1 | Aldh2 |
| 1.68915004192366e-40 | 2.594986 | 0.589 | 0.276 | 5.07E-37 | 1 | Macf1 |
| 2.84884250522571e-40 | 2.516037 | 0.532 | 0.198 | 8.55E-37 | 1 | Icam2 |
| 1.01235123481368e-39 | 4.539236 | 0.548 | 0.195 | 3.04E-36 | 1 | Tspan18 |
| 3.42602073386408e-39 | 3.361197 | 0.565 | 0.255 | 1.03E-35 | 1 | Rasip1 |
| 1.10227109449676e-38 | 3.082050 | 0.544 | 0.25 | 3.31E-35 | 1 | Itga1 |
| 1.34651508643854e-38 | 2.946374 | 0.534 | 0.223 | 4.04E-35 | 1 | Nr1d1 |
| 3.38462678285312e-38 | 3.961029 | 0.577 | 0.293 | 1.02E-34 | 1 | Kank3 |
| 5.43049569855865e-38 | 2.230412 | 0.488 | 0.134 | 1.63E-34 | 1 | Ntrk2 |
| 4.16363217348811e-37 | 2.386274 | 0.498 | 0.168 | 1.25E-33 | 1 | Efnb1 |
| 5.07266791303894e-37 | 3.468145 | 0.504 | 0.182 | 1.52E-33 | 1 | Nipal3 |
| 4.17056579370376e-36 | 2.225275 | 0.607 | 0.298 | 1.25E-32 | 1 | Vegfa |
| 4.35921236246738e-36 | 2.917180 | 0.454 | 0.08 | 1.31E-32 | 1 | Fibin |
| 5.51270338446961e-36 | 2.333042 | 0.46 | 0.145 | 1.65E-32 | 1 | Tmtc1 |
| 1.21430429962304e-35 | 4.335976 | 0.522 | 0.196 | 3.64E-32 | 1 | Prx |
| 1.7345576712168e-35 | 2.500393 | 0.528 | 0.246 | 5.20E-32 | 1 | Krt7 |
| 4.61242232129533e-35 | 2.393412 | 0.534 | 0.237 | 1.38E-31 | 1 | Add3 |
| 8.93437658637628e-35 | 2.168563 | 0.516 | 0.221 | 2.68E-31 | 1 | Spock2 |
| 1.43259082708284e-34 | 2.284970 | 0.522 | 0.208 | 4.30E-31 | 1 | Tspan2 |
| 3.75113329167968e-34 | 2.936897 | 0.585 | 0.292 | 1.13E-30 | 1 | Marcks |
| 4.56911190471237e-34 | 2.299277 | 0.554 | 0.284 | 1.37E-30 | 1 | St5 |
| 1.24058412963937e-32 | 3.227812 | 0.488 | 0.189 | 3.72E-29 | 1 | Rasgrp2 |
| 2.11580759691659e-32 | 2.814694 | 0.492 | 0.16 | 6.35E-29 | 1 | Bmp6 |
| 2.64506660902176e-32 | 2.880911 | 0.569 | 0.306 | 7.94E-29 | 1 | Klhl5 |
| 2.91548359584496e-32 | 4.291784 | 0.528 | 0.257 | 8.75E-29 | 1 | Kit |
| 1.19767699595559e-31 | 2.310350 | 0.506 | 0.222 | 3.59E-28 | 1 | Gimap6 |
| 2.91626923225123e-31 | 2.871837 | 0.494 | 0.203 | 8.75E-28 | 1 | Higd1b |
| 6.67473285182582e-31 | 2.679542 | 0.532 | 0.272 | 2.00E-27 | 1 | Rgs12 |
| 7.62404130575764e-31 | 3.486386 | 0.502 | 0.247 | 2.29E-27 | 1 | Gata2 |
| 1.48465669111574e-30 | 3.344343 | 0.474 | 0.129 | 4.45E-27 | 1 | Itga8 |
| 2.24827412227341e-30 | 3.854155 | 0.536 | 0.257 | 6.74E-27 | 1 | Edn1 |
| 2.4250928778411e-30 | 2.720857 | 0.528 | 0.251 | 7.28E-27 | 1 | Dram1 |
| 2.44165304582744e-30 | 3.561382 | 0.573 | 0.294 | 7.32E-27 | 1 | Lpcat1 |
| 2.95986534423252e-30 | 3.197005 | 0.613 | 0.37 | 8.88E-27 | 1 | Tjp1 |
| 4.90440102030939e-30 | 2.027449 | 0.526 | 0.259 | 1.47E-26 | 1 | Arhgap31 |
| 1.10218360465473e-28 | 2.286168 | 0.504 | 0.225 | 3.31E-25 | 1 | Tbx2 |
| 2.73200160094373e-28 | 3.621873 | 0.502 | 0.259 | 8.20E-25 | 1 | Lpin2 |
| 1.85067573996039e-27 | 2.825730 | 0.504 | 0.23 | 5.55E-24 | 1 | Jcad |
| 1.93210078235686e-27 | 2.707691 | 0.496 | 0.218 | 5.80E-24 | 1 | Ednrb |
| 1.11100766261273e-26 | 4.083667 | 0.448 | 0.178 | 3.33E-23 | 1 | Als2cl |
| 1.27932192118708e-26 | 3.059697 | 0.484 | 0.187 | 3.84E-23 | 1 | Gucy1a1 |
| 2.20826411653763e-26 | 2.464573 | 0.472 | 0.18 | 6.62E-23 | 1 | Nebl |
| 9.78809492633626e-26 | 3.440011 | 0.478 | 0.236 | 2.94E-22 | 1 | Rtkn2 |
| 1.60306160061758e-25 | 2.817357 | 0.464 | 0.225 | 4.81E-22 | 1 | H2-Q7 |
| 1.75570070039156e-25 | 3.229615 | 0.45 | 0.179 | 5.27E-22 | 1 | Fam189a2 |
| 2.13097597476309e-25 | 2.811244 | 0.53 | 0.276 | 6.39E-22 | 1 | Tinagl1 |
| 3.61139062887997e-25 | 2.212477 | 0.565 | 0.308 | 1.08E-21 | 1 | Ptgfrn |
| 9.34513678857441e-25 | 2.587224 | 0.468 | 0.233 | 2.80E-21 | 1 | Rab27a |
| 1.96802110349604e-24 | 2.840468 | 0.433 | 0.102 | 5.90E-21 | 1 | Zbtb16 |
| 5.20155317324301e-24 | 3.253308 | 0.484 | 0.235 | 1.56E-20 | 1 | Kdr |
| 6.39695195865287e-24 | 2.243389 | 0.492 | 0.294 | 1.92E-20 | 1 | Psmb8 |
| 6.58722653430076e-24 | 2.528115 | 0.488 | 0.244 | 1.98E-20 | 1 | Gpr146 |
| 6.81775331707594e-24 | 3.760905 | 0.468 | 0.176 | 2.05E-20 | 1 | Stmn2 |
| 8.78998135148994e-24 | 4.004057 | 0.51 | 0.264 | 2.64E-20 | 1 | Smpdl3a |
| 1.16607255772394e-23 | 2.652212 | 0.484 | 0.245 | 3.50E-20 | 1 | Foxo1 |
| 1.45971196047103e-23 | 2.526477 | 0.538 | 0.314 | 4.38E-20 | 1 | Coro1a |
| 9.03803686941614e-23 | 2.703915 | 0.516 | 0.276 | 2.71E-19 | 1 | Snrk |
| 1.1367405665395e-22 | 2.410779 | 0.48 | 0.211 | 3.41E-19 | 1 | She |
| 1.37255193211349e-22 | 2.450476 | 0.486 | 0.245 | 4.12E-19 | 1 | Mid1ip1 |
| 3.07409662198338e-22 | 2.409410 | 0.565 | 0.306 | 9.22E-19 | 1 | Plpp1 |
| 1.08366431443167e-21 | 2.609426 | 0.452 | 0.171 | 3.25E-18 | 1 | Wfdc21 |
| 2.7321239671139e-21 | 3.173608 | 0.452 | 0.194 | 8.20E-18 | 1 | Tspan8 |
| 3.40246549708515e-21 | 2.188724 | 0.431 | 0.175 | 1.02E-17 | 1 | Lgi3 |
| 4.19985603861645e-21 | 2.634930 | 0.502 | 0.261 | 1.26E-17 | 1 | Pltp |
| 1.35949939833494e-20 | 3.364031 | 0.462 | 0.21 | 4.08E-17 | 1 | Rtl8b |
| 1.4397448507126e-20 | 2.812788 | 0.421 | 0.139 | 4.32E-17 | 1 | Prelp |
| 4.84151490848464e-20 | 2.054493 | 0.47 | 0.288 | 1.45E-16 | 1 | Sash1 |
| 6.7379731038583e-20 | 2.568739 | 0.49 | 0.27 | 2.02E-16 | 1 | Inpp5k |
| 9.41646858868753e-20 | 2.715447 | 0.421 | 0.131 | 2.82E-16 | 1 | Atp1a2 |
| 1.02202441246882e-19 | 3.462224 | 0.446 | 0.187 | 3.07E-16 | 1 | Tmem204 |
| 1.11960768560276e-19 | 2.886584 | 0.478 | 0.23 | 3.36E-16 | 1 | Icam1 |
| 1.54759755948117e-19 | 4.011834 | 0.458 | 0.245 | 4.64E-16 | 1 | Cxcr4 |
| 1.61614952691617e-19 | 2.503828 | 0.452 | 0.231 | 4.85E-16 | 1 | Dennd3 |
| 2.61017346044671e-19 | 4.404876 | 0.391 | 0.115 | 7.83E-16 | 1 | Myzap |
| 3.46927417248566e-19 | 2.208326 | 0.411 | 0.192 | 1.04E-15 | 1 | Itgal |
| 3.68772291676844e-19 | 2.053609 | 0.423 | 0.147 | 1.11E-15 | 1 | Tbx5 |
| 6.49202681439117e-19 | 3.165077 | 0.435 | 0.184 | 1.95E-15 | 1 | Postn |
| 1.0002143108309e-18 | 2.011919 | 0.452 | 0.177 | 3.00E-15 | 1 | Fzd4 |
| 1.00021447364821e-18 | 2.663340 | 0.51 | 0.293 | 3.00E-15 | 1 | Sorbs3 |
| 1.87204363016287e-18 | 2.200575 | 0.433 | 0.134 | 5.62E-15 | 1 | Tspan12 |
| 3.17368584122214e-18 | 2.495749 | 0.536 | 0.335 | 9.52E-15 | 1 | Ppp1r9b |
| 4.65240012668783e-18 | 2.931318 | 0.431 | 0.204 | 1.40E-14 | 1 | Gimap4 |
| 6.84359918554545e-18 | 2.189196 | 0.522 | 0.334 | 2.05E-14 | 1 | Ypel3 |
| 9.53786328453534e-18 | 2.004659 | 0.431 | 0.168 | 2.86E-14 | 1 | Prr15l |
| 1.05790627187297e-17 | 2.306830 | 0.536 | 0.336 | 3.17E-14 | 1 | Eng |
| 1.19952133839033e-17 | 2.621995 | 0.419 | 0.214 | 3.60E-14 | 1 | Rab11fip1 |
| 1.32861282645824e-17 | 2.101071 | 0.442 | 0.246 | 3.99E-14 | 1 | Nedd9 |
| 2.21167950688561e-17 | 2.183269 | 0.52 | 0.316 | 6.64E-14 | 1 | Pcolce2 |
| 3.30847589930838e-17 | 2.138080 | 0.427 | 0.148 | 9.93E-14 | 1 | Cd79b |
| 4.57111429639129e-17 | 2.048128 | 0.5 | 0.291 | 1.37E-13 | 1 | Pdgfb |
| 6.71488042782012e-17 | 2.839230 | 0.462 | 0.252 | 2.01E-13 | 1 | Tmcc2 |
| 9.10208188322115e-17 | 3.343201 | 0.456 | 0.274 | 2.73E-13 | 1 | Numb |
| 1.21732206193034e-16 | 4.986366 | 0.45 | 0.269 | 3.65E-13 | 1 | Peli1 |
| 1.26464163220725e-16 | 3.020269 | 0.435 | 0.209 | 3.79E-13 | 1 | Atp8a1 |
| 1.79822598579024e-16 | 2.459062 | 0.448 | 0.221 | 5.39E-13 | 1 | Pde5a |
| 1.82364735259991e-16 | 2.598733 | 0.393 | 0.103 | 5.47E-13 | 1 | Pde9a |
| 1.9728734919112e-16 | 2.654092 | 0.48 | 0.273 | 5.92E-13 | 1 | Caskin2 |
| 7.65616529890474e-16 | 3.311217 | 0.454 | 0.208 | 2.30E-12 | 1 | Lipe |
| 8.53810590797976e-16 | 3.386342 | 0.488 | 0.317 | 2.56E-12 | 1 | Morf4l1 |
| 9.30178027039535e-16 | 3.115820 | 0.427 | 0.222 | 2.79E-12 | 1 | Erg |
| 1.31902395862595e-15 | 3.102835 | 0.438 | 0.237 | 3.96E-12 | 1 | Csrnp1 |
| 1.69456694459804e-15 | 2.334055 | 0.427 | 0.187 | 5.08E-12 | 1 | Rarres2 |
| 1.71898796907624e-15 | 3.313393 | 0.438 | 0.205 | 5.16E-12 | 1 | Arhgef15 |
| 1.73521003610811e-15 | 3.446012 | 0.456 | 0.24 | 5.21E-12 | 1 | Cdc42bpg |
| 2.09382504264037e-15 | 3.111771 | 0.48 | 0.279 | 6.28E-12 | 1 | Ndrg2 |
| 2.3081929204932e-15 | 3.280636 | 0.458 | 0.283 | 6.92E-12 | 1 | Cybb |
| 2.55990430990091e-15 | 2.006429 | 0.492 | 0.315 | 7.68E-12 | 1 | Foxp1 |
| 2.62694261627157e-15 | 3.502969 | 0.508 | 0.319 | 7.88E-12 | 1 | Hpcal1 |
| 3.67436259250333e-15 | 3.851359 | 0.494 | 0.309 | 1.10E-11 | 1 | Orai1 |
| 4.51463584727146e-15 | 2.682898 | 0.411 | 0.116 | 1.35E-11 | 1 | Ackr2 |
| 6.09000710906055e-15 | 2.304517 | 0.442 | 0.253 | 1.83E-11 | 1 | Car2 |
| 6.40413405925361e-15 | 3.087580 | 0.474 | 0.289 | 1.92E-11 | 1 | Amotl2 |
| 6.63486550228924e-15 | 2.318337 | 0.421 | 0.201 | 1.99E-11 | 1 | Clic3 |
| 1.07947197917806e-14 | 2.571012 | 0.411 | 0.137 | 3.24E-11 | 1 | Tmtc2 |
| 1.27770913953393e-14 | 2.078777 | 0.361 | 0.132 | 3.83E-11 | 1 | Nox4 |
| 2.54579038149896e-14 | 2.099127 | 0.319 | 0.107 | 7.64E-11 | 1 | Chia1 |
| 3.5260846296953e-14 | 2.176350 | 0.391 | 0.135 | 1.06E-10 | 1 | Ighd |
| 4.07421372949041e-14 | 2.762780 | 0.431 | 0.186 | 1.22E-10 | 1 | Gimap8 |
| 4.72660905734058e-14 | 3.669141 | 0.438 | 0.226 | 1.42E-10 | 1 | Akap12 |
| 4.82546556376689e-14 | 2.178938 | 0.486 | 0.318 | 1.45E-10 | 1 | Pink1 |
| 1.02612045399693e-13 | 2.939100 | 0.405 | 0.161 | 3.08E-10 | 1 | Akap5 |
| 1.32690857028094e-13 | 2.275660 | 0.379 | 0.153 | 3.98E-10 | 1 | Slc16a9 |
| 1.96116813391553e-13 | 3.780240 | 0.421 | 0.219 | 5.88E-10 | 1 | Lyz1 |
| 2.48036984252068e-13 | 3.379146 | 0.45 | 0.292 | 7.44E-10 | 1 | Plekhf2 |
| 2.50628043810904e-13 | 2.248901 | 0.395 | 0.147 | 7.52E-10 | 1 | Ccdc85a |
| 3.48553610671893e-13 | 2.537589 | 0.468 | 0.283 | 1.05E-09 | 1 | S100a16 |
| 4.23605127682301e-13 | 2.449121 | 0.387 | 0.158 | 1.27E-09 | 1 | Limch1 |
| 5.08344180567917e-13 | 4.016253 | 0.417 | 0.213 | 1.53E-09 | 1 | Ccdc141 |
| 6.12632157315976e-13 | 2.431865 | 0.393 | 0.19 | 1.84E-09 | 1 | Slco3a1 |
| 6.35613402474517e-13 | 2.699719 | 0.397 | 0.156 | 1.91E-09 | 1 | Cish |
| 6.97152585294205e-13 | 2.633857 | 0.435 | 0.258 | 2.09E-09 | 1 | Pwwp2b |
| 7.76601317065389e-13 | 2.993214 | 0.474 | 0.291 | 2.33E-09 | 1 | Efnb2 |
| 1.06216729291455e-12 | 3.658854 | 0.423 | 0.144 | 3.19E-09 | 1 | B4galt4 |
| 1.13715313399128e-12 | 4.286286 | 0.407 | 0.167 | 3.41E-09 | 1 | Gucy1b1 |
| 2.44239307614839e-12 | 2.187636 | 0.286 | 0.18 | 7.33E-09 | 1 | Abcc3 |
| 2.56840218269111e-12 | 3.507223 | 0.454 | 0.257 | 7.71E-09 | 1 | Cxcl12 |
| 2.86600448083924e-12 | 2.172017 | 0.417 | 0.244 | 8.60E-09 | 1 | Ttyh3 |
| 3.02184715043538e-12 | 2.124364 | 0.407 | 0.174 | 9.07E-09 | 1 | Sox7 |
| 3.11629743274755e-12 | 2.670301 | 0.397 | 0.125 | 9.35E-09 | 1 | Cdkn1c |
| 3.25307729168799e-12 | 2.604068 | 0.435 | 0.292 | 9.76E-09 | 1 | Fam117b |
| 7.0908845301989e-12 | 2.048450 | 0.387 | 0.121 | 2.13E-08 | 1 | Slc24a4 |
| 8.05159561514309e-12 | 2.289032 | 0.5 | 0.342 | 2.42E-08 | 1 | Alox5ap |
| 1.00490259852866e-11 | 2.586774 | 0.419 | 0.285 | 3.01E-08 | 1 | Irak2 |
| 1.17399669815719e-11 | 2.745157 | 0.389 | 0.248 | 3.52E-08 | 1 | Foxn3 |
| 1.85592598610706e-11 | 3.571407 | 0.476 | 0.289 | 5.57E-08 | 1 | Id1 |
| 1.86642665795157e-11 | 3.233242 | 0.442 | 0.194 | 5.60E-08 | 1 | Nsg1 |
| 2.00662591534e-11 | 2.070825 | 0.448 | 0.29 | 6.02E-08 | 1 | Rusc2 |
| 2.02414729893599e-11 | 2.446581 | 0.423 | 0.191 | 6.07E-08 | 1 | Snca |
| 2.26602149206627e-11 | 2.297618 | 0.423 | 0.226 | 6.80E-08 | 1 | Klf4 |
| 2.36668676563294e-11 | 3.249335 | 0.492 | 0.336 | 7.10E-08 | 1 | Cltb |
| 5.65014069664372e-11 | 2.624502 | 0.464 | 0.286 | 1.70E-07 | 1 | Akap13 |
| 8.67353491042031e-11 | 4.372579 | 0.423 | 0.221 | 2.60E-07 | 1 | C130074G19Rik |
| 1.25346007078189e-10 | 2.047881 | 0.391 | 0.216 | 3.76E-07 | 1 | Muc1 |
| 1.80047656688904e-10 | 2.727758 | 0.421 | 0.249 | 5.40E-07 | 1 | Dock6 |
| 2.14595780167439e-10 | 3.092422 | 0.403 | 0.272 | 6.44E-07 | 1 | Pbxip1 |
| 2.40397332890257e-10 | 3.212482 | 0.482 | 0.303 | 7.21E-07 | 1 | Ece1 |
| 2.47063546974057e-10 | 3.481590 | 0.427 | 0.24 | 7.41E-07 | 1 | Dgkh |
| 2.86553807646766e-10 | 2.050522 | 0.391 | 0.136 | 8.60E-07 | 1 | Npr3 |
| 2.90015084017712e-10 | 2.303086 | 0.494 | 0.344 | 8.70E-07 | 1 | Dock9 |
| 4.02431955336956e-10 | 2.145704 | 0.496 | 0.331 | 1.21E-06 | 1 | Ehd4 |
| 4.52334705465592e-10 | 2.419552 | 0.355 | 0.143 | 1.36E-06 | 1 | Dtna |
| 4.92299878820514e-10 | 2.110655 | 0.452 | 0.282 | 1.48E-06 | 1 | Notch1 |
| 6.37386862867023e-10 | 2.743347 | 0.399 | 0.237 | 1.91E-06 | 1 | Ptprg |
| 7.77902261228142e-10 | 3.449491 | 0.363 | 0.197 | 2.33E-06 | 1 | Mctp1 |
| 8.81817839445142e-10 | 4.815118 | 0.444 | 0.302 | 2.65E-06 | 1 | Adgra3 |
| 9.07546239651201e-10 | 2.612796 | 0.47 | 0.285 | 2.72E-06 | 1 | Nfib |
| 9.54129105406614e-10 | 4.029379 | 0.435 | 0.278 | 2.86E-06 | 1 | Pcnx |
| 1.29544827840857e-09 | 3.066936 | 0.438 | 0.29 | 3.89E-06 | 1 | Tubb2a |
| 1.74008832682864e-09 | 3.125712 | 0.405 | 0.19 | 5.22E-06 | 1 | Tspan13 |
| 2.00565114969536e-09 | 3.249296 | 0.425 | 0.226 | 6.02E-06 | 1 | Iqsec2 |
| 2.07159070798393e-09 | 2.906479 | 0.325 | 0.267 | 6.21E-06 | 1 | Itgb3 |
| 2.67615684748258e-09 | 2.658030 | 0.399 | 0.196 | 8.03E-06 | 1 | Heyl |
| 2.85885842634508e-09 | 3.772610 | 0.369 | 0.113 | 8.58E-06 | 1 | Col13a1 |
| 2.91234546189687e-09 | 2.610555 | 0.411 | 0.237 | 8.74E-06 | 1 | Ednra |
| 3.24162365474302e-09 | 3.865751 | 0.421 | 0.223 | 9.72E-06 | 1 | Elmo1 |
| 4.25295875266717e-09 | 2.649328 | 0.429 | 0.283 | 1.28E-05 | 1 | Tmem159 |
| 5.85504453459007e-09 | 4.770787 | 0.387 | 0.148 | 1.76E-05 | 1 | Ly6a |
| 6.02465432164936e-09 | 2.125700 | 0.369 | 0.145 | 1.81E-05 | 1 | Npr2 |
| 6.64647633193067e-09 | 2.973937 | 0.371 | 0.153 | 1.99E-05 | 1 | Scnn1g |
| 6.88438806242522e-09 | 2.407091 | 0.5 | 0.363 | 2.07E-05 | 1 | Arl6ip1 |
| 7.2375811009296e-09 | 2.970329 | 0.415 | 0.222 | 2.17E-05 | 1 | Notch3 |
| 7.29554875422673e-09 | 3.887700 | 0.46 | 0.336 | 2.19E-05 | 1 | Trim25 |
| 7.48250351334658e-09 | 3.704366 | 0.498 | 0.354 | 2.24E-05 | 1 | Ankrd13a |
| 8.37861656645568e-09 | 2.244104 | 0.395 | 0.231 | 2.51E-05 | 1 | Map4k2 |
| 1.05732379962417e-08 | 2.311819 | 0.403 | 0.25 | 3.17E-05 | 1 | Arrb1 |
| 1.30044701529297e-08 | 3.064474 | 0.369 | 0.143 | 3.90E-05 | 1 | Ccm2l |
| 1.73673301998533e-08 | 2.227536 | 0.421 | 0.239 | 5.21E-05 | 1 | Sphk1 |
| 1.882015451504e-08 | 2.053427 | 0.393 | 0.245 | 5.65E-05 | 1 | Slc44a1 |
| 1.97886600534201e-08 | 4.077327 | 0.435 | 0.312 | 5.94E-05 | 1 | Ascc2 |
| 2.52482279763537e-08 | 2.715946 | 0.446 | 0.314 | 7.57E-05 | 1 | Irf3 |
| 2.64763388730476e-08 | 5.151349 | 0.327 | 0.178 | 7.94E-05 | 1 | Shroom2 |
| 3.39103661898916e-08 | 2.493319 | 0.357 | 0.124 | 1.02E-04 | 1 | Patj |
| 4.13871952156094e-08 | 4.015280 | 0.317 | 0.123 | 1.24E-04 | 1 | Hmcn1 |
| 4.21388750242873e-08 | 2.384164 | 0.395 | 0.267 | 1.26E-04 | 1 | Cd302 |
| 4.2155916119157e-08 | 2.213146 | 0.409 | 0.267 | 1.26E-04 | 1 | Serpine2 |
| 4.45640450891039e-08 | 3.999332 | 0.403 | 0.258 | 1.34E-04 | 1 | Irf9 |
| 4.92785908986139e-08 | 2.520878 | 0.512 | 0.376 | 1.48E-04 | 1 | Cds2 |
| 5.15540933321946e-08 | 3.533867 | 0.375 | 0.232 | 1.55E-04 | 1 | Parp14 |
| 7.10750604243537e-08 | 2.831679 | 0.411 | 0.269 | 2.13E-04 | 1 | Psme2b |
| 7.23803327344546e-08 | 4.306379 | 0.391 | 0.224 | 2.17E-04 | 1 | Cep85l |
| 7.60812391058856e-08 | 3.044117 | 0.369 | 0.112 | 2.28E-04 | 1 | Hecw2 |
| 8.40989316641262e-08 | 2.252634 | 0.391 | 0.275 | 2.52E-04 | 1 | Eif4e3 |
| 8.52633156435546e-08 | 2.409852 | 0.478 | 0.33 | 2.56E-04 | 1 | Cdc42ep4 |
| 9.81176690544299e-08 | 2.293533 | 0.371 | 0.221 | 2.94E-04 | 1 | Sorbs1 |
| 1.35280543270072e-07 | 2.375306 | 0.363 | 0.178 | 4.06E-04 | 1 | Abcb1a |
| 1.71465465568669e-07 | 4.834739 | 0.407 | 0.221 | 5.14E-04 | 1 | Adgrl2 |
| 1.99803744649278e-07 | 4.809328 | 0.393 | 0.234 | 5.99E-04 | 1 | Pcdh1 |
| 2.07855932245298e-07 | 2.421396 | 0.399 | 0.257 | 6.24E-04 | 1 | Dock2 |
| 2.45370186495381e-07 | 2.421093 | 0.371 | 0.222 | 7.36E-04 | 1 | Ifi27l2a |
| 2.7248090914268e-07 | 2.804927 | 0.377 | 0.218 | 8.17E-04 | 1 | Abi3 |
| 3.31511978717247e-07 | 4.991123 | 0.385 | 0.276 | 9.95E-04 | 1 | Ddhd1 |
| 4.29610609323854e-07 | 3.725565 | 0.429 | 0.279 | 1.29E-03 | 1 | Fli1 |
| 4.93392568712312e-07 | 2.557935 | 0.369 | 0.145 | 1.48E-03 | 1 | Cxcr2 |
| 6.38150834010355e-07 | 2.244981 | 0.391 | 0.272 | 1.91E-03 | 1 | Ccr2 |
| 7.49909461199995e-07 | 3.554137 | 0.417 | 0.265 | 2.25E-03 | 1 | Klf9 |
| 7.85261738959683e-07 | 2.405864 | 0.417 | 0.287 | 2.36E-03 | 1 | Ptprm |
| 8.0088879609537e-07 | 2.301436 | 0.341 | 0.139 | 2.40E-03 | 1 | Pdzk1ip1 |
| 8.5270024609427e-07 | 2.432497 | 0.377 | 0.143 | 2.56E-03 | 1 | Ttll7 |
| 8.74555584195612e-07 | 2.073971 | 0.381 | 0.208 | 2.62E-03 | 1 | Itpkb |
| 9.58905367538039e-07 | 2.101860 | 0.321 | 0.134 | 2.88E-03 | 1 | Klhdc8b |
| 9.8417957369943e-07 | 4.243108 | 0.474 | 0.336 | 2.95E-03 | 1 | Shank3 |
| 1.03290899171329e-06 | 2.923405 | 0.415 | 0.297 | 3.10E-03 | 1 | Klhl24 |
| 1.08582378035692e-06 | 3.893520 | 0.429 | 0.303 | 3.26E-03 | 1 | Klf3 |
| 1.08927152360239e-06 | 2.521165 | 0.292 | 0.133 | 3.27E-03 | 1 | Fpr2 |
| 1.40793700649726e-06 | 2.937657 | 0.403 | 0.278 | 4.22E-03 | 1 | Plek |
| 1.42431904201561e-06 | 2.875152 | 0.458 | 0.344 | 4.27E-03 | 1 | Arap3 |
| 1.52625432211556e-06 | 3.684079 | 0.349 | 0.14 | 4.58E-03 | 1 | H2-T24 |
| 1.60598108897025e-06 | 4.714633 | 0.363 | 0.223 | 4.82E-03 | 1 | 4930523C07Rik |
| 1.67128930264147e-06 | 2.430126 | 0.383 | 0.254 | 5.01E-03 | 1 | Nfam1 |
| 1.7705145828155e-06 | 2.005176 | 0.3 | 0.205 | 5.31E-03 | 1 | Fabp4 |
| 1.79890700808228e-06 | 2.060521 | 0.423 | 0.304 | 5.40E-03 | 1 | Sdc3 |
| 2.34203583131748e-06 | 2.253410 | 0.383 | 0.236 | 7.03E-03 | 1 | Pcdh18 |
| 2.97351243700087e-06 | 2.396832 | 0.391 | 0.193 | 8.92E-03 | 1 | Gimap1 |
| 3.15716558453376e-06 | 2.680417 | 0.468 | 0.336 | 9.47E-03 | 1 | Bcl2l1 |
| 4.21350500997433e-06 | 2.748921 | 0.387 | 0.278 | 1.26E-02 | 1 | Hcls1 |
| 4.45615995349495e-06 | 2.567138 | 0.244 | 0.167 | 1.34E-02 | 1 | Ltbp2 |
| 4.86259291127891e-06 | 4.403368 | 0.419 | 0.333 | 1.46E-02 | 1 | Prpf18 |
| 6.23296721500706e-06 | 3.129229 | 0.399 | 0.287 | 1.87E-02 | 1 | Osbpl8 |
| 6.9274329335298e-06 | 2.238772 | 0.403 | 0.294 | 2.08E-02 | 1 | Akap9 |
| 6.9612812766537e-06 | 2.193650 | 0.458 | 0.345 | 2.09E-02 | 1 | Pnrc2 |
| 7.11855305503446e-06 | 2.239548 | 0.427 | 0.299 | 2.14E-02 | 1 | Irf1 |
| 7.43026532529514e-06 | 2.970080 | 0.369 | 0.135 | 2.23E-02 | 1 | Pde3a |
| 1.00753862258885e-05 | 2.416646 | 0.381 | 0.254 | 3.02E-02 | 1 | Pik3ap1 |
| 1.01850625760784e-05 | 3.713116 | 0.341 | 0.228 | 3.06E-02 | 1 | Tcaf1 |
| 1.04190198117894e-05 | 3.868961 | 0.389 | 0.265 | 3.13E-02 | 1 | Tef |
| 1.21893824862323e-05 | 2.294456 | 0.397 | 0.192 | 3.66E-02 | 1 | S1pr3 |
| 1.22745807850999e-05 | 2.996310 | 0.383 | 0.238 | 3.68E-02 | 1 | Notch4 |
| 1.23694618489366e-05 | 2.883074 | 0.417 | 0.294 | 3.71E-02 | 1 | Rnf167 |
| 1.48319681454969e-05 | 2.187343 | 0.357 | 0.136 | 4.45E-02 | 1 | Slc6a14 |
| 1.49465864461319e-62 | 2.597521 | 0.861 | 0.415 | 4.48E-59 | 10 | Col3a1 |
| 7.6348232761711e-57 | 4.429211 | 0.876 | 0.421 | 2.29E-53 | 10 | Fn1 |
| 6.04819609715659e-54 | 2.624158 | 0.861 | 0.404 | 1.81E-50 | 10 | Wls |
| 3.60628589738775e-34 | 2.995830 | 0.722 | 0.197 | 1.08E-30 | 10 | Cilp |
| 2.08859865847136e-29 | 4.232538 | 0.646 | 0.206 | 6.27E-26 | 10 | Retnla |
| 6.5190398919909e-24 | 2.213022 | 0.818 | 0.486 | 1.96E-20 | 10 | Ctsl |
| 5.78363162978389e-15 | 2.043595 | 0.694 | 0.449 | 1.74E-11 | 10 | Spp1 |
| 7.31605240357195e-13 | 2.565482 | 0.622 | 0.372 | 2.19E-09 | 10 | Dpt |
| 2.34955174992505e-10 | 3.800945 | 0.498 | 0.122 | 7.05E-07 | 10 | Pllp |
| 8.48257465119892e-09 | 2.025706 | 0.584 | 0.339 | 2.54E-05 | 10 | Trp53i11 |
| 3.9248992854909e-06 | 2.204028 | 0.536 | 0.305 | 1.18E-02 | 10 | Syt13 |
| 2.37521663908492e-94 | 3.650404 | 0.989 | 0.332 | 7.13E-91 | 11 | Sftpa1 |
| 1.55199593736835e-93 | 3.619076 | 0.989 | 0.337 | 4.66E-90 | 11 | Sftpc |
| 4.20921807132383e-93 | 3.222341 | 0.973 | 0.295 | 1.26E-89 | 11 | Inmt |
| 1.37036327179552e-90 | 4.797336 | 0.968 | 0.255 | 4.11E-87 | 11 | C3 |
| 6.97710287036573e-88 | 4.519293 | 0.941 | 0.299 | 2.09E-84 | 11 | Ager |
| 4.15453121879743e-85 | 3.408017 | 0.952 | 0.295 | 1.25E-81 | 11 | Mgp |
| 1.92563558079974e-81 | 3.922121 | 0.957 | 0.321 | 5.78E-78 | 11 | Slc34a2 |
| 2.02003881873608e-76 | 2.869253 | 0.952 | 0.28 | 6.06E-73 | 11 | Fmo2 |
| 6.98796788447038e-75 | 2.382245 | 0.941 | 0.294 | 2.10E-71 | 11 | Tns1 |
| 8.16019834862794e-75 | 3.414905 | 0.925 | 0.278 | 2.45E-71 | 11 | Bgn |
| 1.06017272776709e-72 | 2.293404 | 0.952 | 0.307 | 3.18E-69 | 11 | Gpx3 |
| 1.26237324568974e-70 | 3.250082 | 0.914 | 0.271 | 3.79E-67 | 11 | Timp3 |
| 3.39525290153444e-69 | 3.639435 | 0.887 | 0.219 | 1.02E-65 | 11 | C7 |
| 3.8601252780916e-68 | 2.413541 | 0.919 | 0.314 | 1.16E-64 | 11 | App |
| 3.15710452182178e-65 | 2.103099 | 0.925 | 0.369 | 9.47E-62 | 11 | Hba-a2 |
| 6.90187693536002e-65 | 3.895104 | 0.855 | 0.255 | 2.07E-61 | 11 | Gas6 |
| 2.38213239377475e-63 | 4.496543 | 0.903 | 0.291 | 7.15E-60 | 11 | Eln |
| 1.38688992837857e-61 | 2.472863 | 0.909 | 0.287 | 4.16E-58 | 11 | Sparcl1 |
| 2.13427937003195e-61 | 4.530723 | 0.806 | 0.187 | 6.40E-58 | 11 | Saa3 |
| 7.81267004243188e-61 | 3.969410 | 0.828 | 0.195 | 2.34E-57 | 11 | Slc39a8 |
| 1.46526481921924e-60 | 3.490304 | 0.839 | 0.219 | 4.40E-57 | 11 | Serpina3n |
| 7.91459627322582e-60 | 4.916194 | 0.86 | 0.295 | 2.37E-56 | 11 | Rgcc |
| 9.15083975402877e-58 | 2.106907 | 0.849 | 0.29 | 2.75E-54 | 11 | Chil1 |
| 3.44806439180197e-57 | 2.637260 | 0.866 | 0.229 | 1.03E-53 | 11 | Clic5 |
| 2.3906207233429e-55 | 2.355272 | 0.882 | 0.322 | 7.17E-52 | 11 | Sptbn1 |
| 1.53749740179818e-54 | 2.464203 | 0.844 | 0.309 | 4.61E-51 | 11 | Cxcl15 |
| 4.6206008947311e-53 | 2.588538 | 0.828 | 0.253 | 1.39E-49 | 11 | Krt7 |
| 2.54638724145409e-52 | 3.572376 | 0.833 | 0.297 | 7.64E-49 | 11 | Dusp1 |
| 5.01513073145273e-52 | 2.838142 | 0.839 | 0.278 | 1.50E-48 | 11 | Zfp36 |
| 1.23918252421715e-50 | 2.371473 | 0.823 | 0.274 | 3.72E-47 | 11 | Cfh |
| 1.29056355873362e-50 | 2.839823 | 0.806 | 0.24 | 3.87E-47 | 11 | Scnn1a |
| 1.62855753039409e-50 | 3.609966 | 0.817 | 0.229 | 4.89E-47 | 11 | Nbl1 |
| 3.68777532780998e-50 | 3.778688 | 0.758 | 0.218 | 1.11E-46 | 11 | Fosb |
| 4.50590677490075e-49 | 2.564163 | 0.785 | 0.224 | 1.35E-45 | 11 | Ptgis |
| 6.2080238669911e-49 | 3.250214 | 0.79 | 0.204 | 1.86E-45 | 11 | S100g |
| 6.38873636499485e-49 | 3.808484 | 0.801 | 0.252 | 1.92E-45 | 11 | Cldn18 |
| 5.7737182063666e-48 | 4.942308 | 0.758 | 0.206 | 1.73E-44 | 11 | Gpc3 |
| 6.89119097720663e-48 | 4.194834 | 0.79 | 0.222 | 2.07E-44 | 11 | Npnt |
| 7.49418744217747e-48 | 2.324152 | 0.823 | 0.251 | 2.25E-44 | 11 | Lamp3 |
| 4.19924024485779e-45 | 4.208523 | 0.79 | 0.261 | 1.26E-41 | 11 | Fos |
| 1.89651899341621e-44 | 3.345340 | 0.801 | 0.254 | 5.69E-41 | 11 | Hopx |
| 2.80568543874229e-43 | 2.443052 | 0.855 | 0.367 | 8.42E-40 | 11 | Jun |
| 9.07434097880831e-43 | 3.274454 | 0.737 | 0.168 | 2.72E-39 | 11 | Adamts15 |
| 9.15217834649258e-43 | 2.723217 | 0.812 | 0.325 | 2.75E-39 | 11 | Klf2 |
| 4.23581172848116e-42 | 2.244401 | 0.769 | 0.249 | 1.27E-38 | 11 | Lpl |
| 9.26178413208544e-42 | 3.335899 | 0.78 | 0.234 | 2.78E-38 | 11 | C4b |
| 1.2016282729221e-40 | 3.083722 | 0.79 | 0.312 | 3.60E-37 | 11 | Egr1 |
| 1.28912294698863e-40 | 2.722184 | 0.79 | 0.28 | 3.87E-37 | 11 | Slc43a3 |
| 2.53281326806471e-40 | 2.852389 | 0.758 | 0.22 | 7.60E-37 | 11 | Spon1 |
| 6.46422614845388e-39 | 3.691383 | 0.823 | 0.371 | 1.94E-35 | 11 | Junb |
| 1.85605187440055e-38 | 3.969683 | 0.753 | 0.261 | 5.57E-35 | 11 | Btg2 |
| 4.49677499565605e-37 | 3.257962 | 0.715 | 0.216 | 1.35E-33 | 11 | Rab11fip1 |
| 2.60149118545905e-36 | 3.003998 | 0.742 | 0.237 | 7.80E-33 | 11 | Icam1 |
| 2.89093258204524e-36 | 3.181935 | 0.758 | 0.257 | 8.67E-33 | 11 | Cmklr1 |
| 1.74601913233147e-35 | 2.073262 | 0.763 | 0.373 | 5.24E-32 | 11 | Cst3 |
| 1.96256461401841e-35 | 3.615348 | 0.79 | 0.321 | 5.89E-32 | 11 | H6pd |
| 1.71439906810983e-34 | 3.360627 | 0.731 | 0.261 | 5.14E-31 | 11 | Slpi |
| 6.1558004327658e-34 | 2.491581 | 0.753 | 0.231 | 1.85E-30 | 11 | Thbd |
| 6.90791547002427e-34 | 2.735236 | 0.742 | 0.349 | 2.07E-30 | 11 | Txnip |
| 2.54338257623412e-33 | 2.303506 | 0.737 | 0.258 | 7.63E-30 | 11 | Sftpd |
| 3.59120314662818e-33 | 2.472751 | 0.737 | 0.247 | 1.08E-29 | 11 | Cryab |
| 2.04045289055272e-31 | 3.024892 | 0.694 | 0.244 | 6.12E-28 | 11 | Cxcl14 |
| 4.06702099683652e-31 | 2.787016 | 0.72 | 0.235 | 1.22E-27 | 11 | Tbx2 |
| 7.63606108889058e-31 | 2.103260 | 0.672 | 0.188 | 2.29E-27 | 11 | Crispld2 |
| 3.15775847632876e-30 | 2.096777 | 0.699 | 0.206 | 9.47E-27 | 11 | Myh14 |
| 8.86103236320547e-30 | 5.432333 | 0.661 | 0.09 | 2.66E-26 | 11 | Gpm6a |
| 1.2498717858904e-29 | 6.650894 | 0.683 | 0.142 | 3.75E-26 | 11 | Cfb |
| 1.31062551260178e-28 | 4.768510 | 0.699 | 0.178 | 3.93E-25 | 11 | Hspb1 |
| 4.04157296899939e-28 | 2.046605 | 0.683 | 0.18 | 1.21E-24 | 11 | Adh1 |
| 5.76204294258995e-28 | 3.654223 | 0.656 | 0.206 | 1.73E-24 | 11 | Nkx2-1 |
| 1.38354081017341e-26 | 2.461208 | 0.699 | 0.213 | 4.15E-23 | 11 | Krt80 |
| 2.86552253020117e-26 | 3.756705 | 0.667 | 0.159 | 8.60E-23 | 11 | Cxadr |
| 6.00593313342075e-26 | 3.914087 | 0.694 | 0.238 | 1.80E-22 | 11 | Ptprf |
| 8.80063113731596e-26 | 4.747540 | 0.597 | 0.109 | 2.64E-22 | 11 | Podn |
| 2.83583326941361e-25 | 2.761658 | 0.538 | 0.167 | 8.51E-22 | 11 | Ccdc80 |
| 8.21402671619734e-25 | 2.219481 | 0.656 | 0.194 | 2.46E-21 | 11 | Rarres2 |
| 1.65905719289798e-24 | 3.220499 | 0.624 | 0.196 | 4.98E-21 | 11 | Fgfr2 |
| 1.72337335503859e-24 | 2.395561 | 0.64 | 0.214 | 5.17E-21 | 11 | Aqp5 |
| 5.5419385260737e-24 | 2.262001 | 0.726 | 0.33 | 1.66E-20 | 11 | Tacc1 |
| 8.66979720476543e-24 | 5.078293 | 0.586 | 0.115 | 2.60E-20 | 11 | Lox |
| 1.06978185511893e-23 | 3.369251 | 0.597 | 0.161 | 3.21E-20 | 11 | Bicc1 |
| 4.1218867354861e-23 | 2.166564 | 0.683 | 0.239 | 1.24E-19 | 11 | Dipk2a |
| 1.75364401302499e-22 | 3.026630 | 0.575 | 0.111 | 5.26E-19 | 11 | Chia1 |
| 2.0687510720574e-22 | 2.141777 | 0.651 | 0.136 | 6.21E-19 | 11 | Klf15 |
| 8.5713749651407e-22 | 3.094021 | 0.624 | 0.205 | 2.57E-18 | 11 | Tspan8 |
| 1.39961239184165e-21 | 2.804606 | 0.624 | 0.14 | 4.20E-18 | 11 | Slc6a14 |
| 3.02269164276623e-21 | 5.010524 | 0.661 | 0.214 | 9.07E-18 | 11 | Sox17 |
| 1.23192007099399e-20 | 2.323174 | 0.704 | 0.311 | 3.70E-17 | 11 | Plk2 |
| 3.72080510804735e-20 | 2.324662 | 0.613 | 0.192 | 1.12E-16 | 11 | Ism1 |
| 1.14986875739059e-19 | 5.541776 | 0.618 | 0.185 | 3.45E-16 | 11 | Msln |
| 2.72175749882368e-19 | 4.510148 | 0.667 | 0.252 | 8.17E-16 | 11 | Atf3 |
| 2.06260195721708e-17 | 2.179691 | 0.677 | 0.306 | 6.19E-14 | 11 | Tmem176b |
| 2.20571306095696e-17 | 3.333341 | 0.667 | 0.342 | 6.62E-14 | 11 | Id3 |
| 2.53111510674577e-17 | 4.101912 | 0.602 | 0.229 | 7.59E-14 | 11 | Nr4a1 |
| 2.81413904623539e-17 | 4.406084 | 0.602 | 0.16 | 8.44E-14 | 11 | Plxdc2 |
| 3.12457273580491e-17 | 3.713912 | 0.645 | 0.268 | 9.37E-14 | 11 | Pdpn |
| 5.04265926873229e-17 | 4.552075 | 0.543 | 0.121 | 1.51E-13 | 11 | Btla |
| 5.67783104927401e-17 | 3.363867 | 0.586 | 0.177 | 1.70E-13 | 11 | Cped1 |
| 5.99138550386772e-17 | 2.473221 | 0.661 | 0.288 | 1.80E-13 | 11 | Tinagl1 |
| 1.38983488196326e-16 | 2.248123 | 0.613 | 0.144 | 4.17E-13 | 11 | Col4a4 |
| 2.51843150231296e-16 | 4.332638 | 0.559 | 0.12 | 7.56E-13 | 11 | Tmod1 |
| 2.73202631974587e-16 | 2.724799 | 0.613 | 0.194 | 8.20E-13 | 11 | Postn |
| 6.00810562112052e-16 | 5.210797 | 0.586 | 0.149 | 1.80E-12 | 11 | Rgs4 |
| 6.8123388899973e-16 | 2.975467 | 0.489 | 0.151 | 2.04E-12 | 11 | Rtn4rl2 |
| 9.11961429384372e-16 | 2.437343 | 0.591 | 0.244 | 2.74E-12 | 11 | Rab27a |
| 1.35695467263067e-15 | 3.955642 | 0.57 | 0.189 | 4.07E-12 | 11 | Cdh11 |
| 1.91878515695081e-15 | 2.427335 | 0.586 | 0.179 | 5.76E-12 | 11 | Sec14l4 |
| 3.17621964056279e-15 | 3.036597 | 0.602 | 0.186 | 9.53E-12 | 11 | Lgi3 |
| 5.78123970957035e-15 | 2.394993 | 0.634 | 0.303 | 1.73E-11 | 11 | Plvap |
| 2.06783765912151e-14 | 3.193859 | 0.597 | 0.171 | 6.20E-11 | 11 | Tbx4 |
| 3.56439875691516e-14 | 3.370208 | 0.57 | 0.132 | 1.07E-10 | 11 | Mapt |
| 3.79240135996406e-14 | 2.651241 | 0.575 | 0.223 | 1.14E-10 | 11 | Slc2a3 |
| 5.3011577063383e-14 | 2.744098 | 0.538 | 0.153 | 1.59E-10 | 11 | Prickle2 |
| 5.73462358263212e-14 | 2.176622 | 0.559 | 0.169 | 1.72E-10 | 11 | Scnn1b |
| 5.92015177051779e-14 | 2.239857 | 0.548 | 0.153 | 1.78E-10 | 11 | Npr2 |
| 7.47273556980637e-14 | 2.360639 | 0.634 | 0.323 | 2.24E-10 | 11 | Ptgfrn |
| 9.21696000022737e-14 | 3.783167 | 0.532 | 0.159 | 2.77E-10 | 11 | Ndnf |
| 9.86185775047417e-14 | 2.189867 | 0.57 | 0.168 | 2.96E-10 | 11 | Pdzd2 |
| 1.09027361568876e-13 | 2.561563 | 0.667 | 0.357 | 3.27E-10 | 11 | Socs3 |
| 1.91404778132988e-13 | 4.071122 | 0.554 | 0.121 | 5.74E-10 | 11 | Zbtb16 |
| 6.62327850044237e-13 | 2.953409 | 0.591 | 0.253 | 1.99E-09 | 11 | Nedd9 |
| 6.83543768807456e-13 | 3.352943 | 0.591 | 0.226 | 2.05E-09 | 11 | Loxl1 |
| 1.03005002594345e-12 | 4.962270 | 0.532 | 0.188 | 3.09E-09 | 11 | Pdk2 |
| 2.37114868579734e-12 | 2.419711 | 0.554 | 0.143 | 7.11E-09 | 11 | Epb41l4b |
| 7.57231903106299e-12 | 2.299419 | 0.591 | 0.28 | 2.27E-08 | 11 | Ankrd37 |
| 1.18690218948682e-11 | 2.314769 | 0.559 | 0.178 | 3.56E-08 | 11 | Pard6b |
| 1.20180090383015e-11 | 2.594559 | 0.527 | 0.158 | 3.61E-08 | 11 | Col4a3 |
| 1.31415443860094e-11 | 4.012928 | 0.57 | 0.2 | 3.94E-08 | 11 | Tacstd2 |
| 2.13330605691121e-11 | 5.201872 | 0.484 | 0.172 | 6.40E-08 | 11 | Cacna1d |
| 2.1427760561203e-11 | 2.264360 | 0.57 | 0.202 | 6.43E-08 | 11 | Slit3 |
| 2.8754411028828e-11 | 3.080255 | 0.581 | 0.223 | 8.63E-08 | 11 | Depp1 |
| 6.7639831422259e-11 | 3.217201 | 0.602 | 0.26 | 2.03E-07 | 11 | Car2 |
| 7.74216013465642e-11 | 3.768046 | 0.5 | 0.185 | 2.32E-07 | 11 | Spon2 |
| 1.59325271593669e-10 | 3.180351 | 0.532 | 0.182 | 4.78E-07 | 11 | Smagp |
| 1.6738774171242e-10 | 2.713852 | 0.543 | 0.167 | 5.02E-07 | 11 | Limch1 |
| 1.7280254031222e-10 | 2.965849 | 0.473 | 0.122 | 5.18E-07 | 11 | Glb1l2 |
| 1.86704667278271e-10 | 4.126208 | 0.538 | 0.154 | 5.60E-07 | 11 | Dpp4 |
| 1.97452911964062e-10 | 3.749616 | 0.538 | 0.139 | 5.92E-07 | 11 | Cdkn1c |
| 2.19544954280874e-10 | 3.100653 | 0.613 | 0.292 | 6.59E-07 | 11 | Nfib |
| 3.08738595697236e-10 | 2.731376 | 0.591 | 0.282 | 9.26E-07 | 11 | Caskin2 |
| 5.76460828936608e-10 | 4.790898 | 0.511 | 0.104 | 1.73E-06 | 11 | Myrf |
| 6.3166364879939e-10 | 2.035119 | 0.28 | 0.416 | 1.89E-06 | 11 | Dab2 |
| 6.4337606874551e-10 | 2.883641 | 0.527 | 0.146 | 1.93E-06 | 11 | Vstm4 |
| 8.76741395385395e-10 | 2.099210 | 0.527 | 0.159 | 2.63E-06 | 11 | Ccdc85a |
| 9.03351481072506e-10 | 2.219683 | 0.629 | 0.366 | 2.71E-06 | 11 | Col1a1 |
| 9.4299799074543e-10 | 2.412761 | 0.516 | 0.155 | 2.83E-06 | 11 | Prelp |
| 1.06786976234568e-09 | 2.255317 | 0.409 | 0.17 | 3.20E-06 | 11 | Cpxm1 |
| 1.10691659380876e-09 | 2.345154 | 0.618 | 0.322 | 3.32E-06 | 11 | Plpp1 |
| 1.4170327590613e-09 | 2.591474 | 0.565 | 0.258 | 4.25E-06 | 11 | Foxo1 |
| 1.87365346938834e-09 | 3.935504 | 0.527 | 0.266 | 5.62E-06 | 11 | Lnx2 |
| 2.30004176711025e-09 | 2.303658 | 0.554 | 0.186 | 6.90E-06 | 11 | Wfdc21 |
| 2.46525749951587e-09 | 3.181987 | 0.538 | 0.147 | 7.40E-06 | 11 | Dkk3 |
| 3.59745253591647e-09 | 2.360281 | 0.602 | 0.345 | 1.08E-05 | 11 | Mfge8 |
| 4.74906978664435e-09 | 2.072075 | 0.511 | 0.239 | 1.42E-05 | 11 | H2-Q7 |
| 5.83133161985801e-09 | 4.088077 | 0.527 | 0.129 | 1.75E-05 | 11 | Cdkn2b |
| 6.00071761629389e-09 | 3.078718 | 0.554 | 0.276 | 1.80E-05 | 11 | Trim56 |
| 6.28596321364193e-09 | 2.356921 | 0.516 | 0.16 | 1.89E-05 | 11 | Prss8 |
| 6.85043963129992e-09 | 2.332320 | 0.511 | 0.173 | 2.06E-05 | 11 | Enpep |
| 9.15875995869095e-09 | 2.116122 | 0.532 | 0.178 | 2.75E-05 | 11 | Epn3 |
| 1.15988351523203e-08 | 2.070941 | 0.511 | 0.128 | 3.48E-05 | 11 | Gramd2 |
| 2.77931152546032e-08 | 2.054175 | 0.532 | 0.157 | 8.34E-05 | 11 | Ntrk2 |
| 3.05864755215987e-08 | 2.666574 | 0.452 | 0.102 | 9.18E-05 | 11 | Ptpru |
| 3.25841152341231e-08 | 2.846425 | 0.532 | 0.202 | 9.78E-05 | 11 | Tmem204 |
| 3.43101592119142e-08 | 2.932536 | 0.543 | 0.271 | 1.03E-04 | 11 | Serpine2 |
| 3.96560879161389e-08 | 5.648040 | 0.522 | 0.14 | 1.19E-04 | 11 | Slc10a6 |
| 4.87370154106811e-08 | 3.003713 | 0.532 | 0.183 | 1.46E-04 | 11 | Myo1d |
| 6.70201584772743e-08 | 2.548671 | 0.532 | 0.247 | 2.01E-04 | 11 | Sphk1 |
| 1.05025351823446e-07 | 2.023873 | 0.489 | 0.088 | 3.15E-04 | 11 | Cobl |
| 1.14286611750122e-07 | 3.744095 | 0.473 | 0.131 | 3.43E-04 | 11 | Tnxb |
| 1.26205044047991e-07 | 3.503260 | 0.425 | 0.149 | 3.79E-04 | 11 | Ltb |
| 1.3274929340104e-07 | 3.362153 | 0.575 | 0.325 | 3.98E-04 | 11 | Cttnbp2nl |
| 2.38058413622159e-07 | 4.079667 | 0.575 | 0.352 | 7.14E-04 | 11 | Fuca1 |
| 2.43971312784322e-07 | 2.480382 | 0.522 | 0.252 | 7.32E-04 | 11 | Ttyh3 |
| 2.80057763403439e-07 | 2.697409 | 0.489 | 0.168 | 8.40E-04 | 11 | Glp1r |
| 3.00857975683398e-07 | 2.504969 | 0.489 | 0.14 | 9.03E-04 | 11 | Fam174b |
| 5.55879865045149e-07 | 2.013870 | 0.516 | 0.195 | 1.67E-03 | 11 | Lrrc32 |
| 6.58179193904064e-07 | 2.580905 | 0.522 | 0.142 | 1.97E-03 | 11 | Prickle1 |
| 1.10454265734438e-06 | 2.042525 | 0.478 | 0.171 | 3.31E-03 | 11 | Kcnj15 |
| 1.15555574605076e-06 | 3.915876 | 0.484 | 0.132 | 3.47E-03 | 11 | Arl4d |
| 1.16364062755206e-06 | 2.220627 | 0.532 | 0.214 | 3.49E-03 | 11 | Map6 |
| 1.43698221863729e-06 | 2.956562 | 0.522 | 0.244 | 4.31E-03 | 11 | Ednra |
| 1.57988617160787e-06 | 3.608776 | 0.548 | 0.327 | 4.74E-03 | 11 | Hsd17b11 |
| 2.11961975053559e-06 | 2.568829 | 0.495 | 0.356 | 6.36E-03 | 11 | Metap1 |
| 2.22488197870796e-06 | 3.526496 | 0.5 | 0.142 | 6.67E-03 | 11 | Cd79a |
| 2.36737840388874e-06 | 3.642335 | 0.446 | 0.127 | 7.10E-03 | 11 | Prkd1 |
| 2.8058611162061e-06 | 3.971057 | 0.565 | 0.269 | 8.42E-03 | 11 | Tmem140 |
| 5.42274781011247e-06 | 4.687817 | 0.473 | 0.106 | 1.63E-02 | 11 | Rasgef1a |
| 6.55800118556754e-06 | 2.646562 | 0.5 | 0.152 | 1.97E-02 | 11 | Tmtc2 |
| 9.06065813351964e-06 | 2.926072 | 0.505 | 0.242 | 2.72E-02 | 11 | Ephb4 |
| 9.56667031338763e-06 | 3.509678 | 0.328 | 0.091 | 2.87E-02 | 11 | Tubb1 |
| 1.23794369049861e-05 | 2.326742 | 0.543 | 0.294 | 3.71E-02 | 11 | Myh10 |
| 1.26113797810684e-05 | 3.528029 | 0.425 | 0.236 | 3.78E-02 | 11 | Vav1 |
| 1.43844391916084e-05 | 2.198871 | 0.478 | 0.283 | 4.32E-02 | 11 | Kctd12 |
| 8.54696245838594e-52 | 2.146825 | 0.994 | 0.547 | 2.56E-48 | 12 | Eef1a1 |
| 1.20854204165024e-47 | 2.221949 | 0.878 | 0.344 | 3.63E-44 | 12 | Ctss |
| 4.31786265457856e-46 | 2.233897 | 0.977 | 0.516 | 1.30E-42 | 12 | Ahnak |
| 2.97425633252723e-39 | 2.337704 | 0.843 | 0.379 | 8.92E-36 | 12 | Col6a2 |
| 1.10256191396116e-36 | 2.647405 | 0.831 | 0.419 | 3.31E-33 | 12 | B2m |
| 1.2105674791289e-34 | 2.179618 | 0.843 | 0.392 | 3.63E-31 | 12 | Galnt1 |
| 7.34773524248486e-27 | 2.113355 | 0.797 | 0.413 | 2.20E-23 | 12 | Axl |
| 5.13443910747622e-21 | 4.482514 | 0.68 | 0.238 | 1.54E-17 | 12 | Gbp2 |
| 2.19230573683712e-13 | 5.124946 | 0.64 | 0.243 | 6.58E-10 | 12 | Igtp |
| 2.53996654154704e-11 | 4.632691 | 0.61 | 0.212 | 7.62E-08 | 12 | Serpina3g |
| 2.38674892944024e-09 | 2.337430 | 0.622 | 0.327 | 7.16E-06 | 12 | Aif1 |
| 3.5653607852333e-09 | 2.089308 | 0.605 | 0.252 | 1.07E-05 | 12 | Irgm1 |
| 1.04614582114152e-07 | 2.506597 | 0.541 | 0.186 | 3.14E-04 | 12 | Gbp4 |
| 1.93328247498239e-06 | 2.489652 | 0.564 | 0.253 | 5.80E-03 | 12 | Gbp7 |
| 2.57375032909091e-97 | 8.324312 | 0.994 | 0.351 | 7.72E-94 | 13 | Arg1 |
| 6.00433554394674e-82 | 4.241936 | 0.988 | 0.394 | 1.80E-78 | 13 | Thbs1 |
| 2.85369386224887e-80 | 4.366211 | 0.988 | 0.354 | 8.56E-77 | 13 | Hmox1 |
| 5.51306233895467e-76 | 6.632618 | 0.959 | 0.385 | 1.65E-72 | 13 | Pf4 |
| 1.62308739587285e-71 | 2.962955 | 0.988 | 0.414 | 4.87E-68 | 13 | Pgam1 |
| 2.77817377916034e-70 | 6.440185 | 0.948 | 0.386 | 8.33E-67 | 13 | Ccl6 |
| 2.5578223595871e-66 | 6.490232 | 0.936 | 0.323 | 7.67E-63 | 13 | Wfdc17 |
| 1.39263588726929e-55 | 3.843550 | 0.907 | 0.339 | 4.18E-52 | 13 | Srgn |
| 3.72153160825698e-54 | 2.188358 | 0.89 | 0.257 | 1.12E-50 | 13 | Slpi |
| 3.16360514079443e-53 | 3.101871 | 0.878 | 0.189 | 9.49E-50 | 13 | Ms4a8a |
| 1.09139204806826e-50 | 4.395852 | 0.826 | 0.242 | 3.27E-47 | 13 | Ppbp |
| 1.16110005171409e-46 | 2.332360 | 0.86 | 0.333 | 3.48E-43 | 13 | Me2 |
| 1.05307282460038e-45 | 3.943509 | 0.826 | 0.344 | 3.16E-42 | 13 | Adam8 |
| 8.44597259779882e-45 | 2.599684 | 0.89 | 0.336 | 2.53E-41 | 13 | Serpine1 |
| 1.4454440105015e-44 | 2.097257 | 0.942 | 0.509 | 4.34E-41 | 13 | Ncl |
| 8.47505095398699e-40 | 2.028088 | 0.913 | 0.495 | 2.54E-36 | 13 | Anxa2 |
| 1.40244044930019e-38 | 2.740192 | 0.837 | 0.324 | 4.21E-35 | 13 | Ndrg1 |
| 4.12112075438946e-38 | 2.094604 | 0.855 | 0.34 | 1.24E-34 | 13 | Pdk1 |
| 3.15328306442522e-36 | 5.013436 | 0.831 | 0.356 | 9.46E-33 | 13 | Ccl9 |
| 9.92945079631609e-32 | 3.854816 | 0.762 | 0.195 | 2.98E-28 | 13 | Crabp1 |
| 1.09313499948166e-30 | 2.055855 | 0.808 | 0.357 | 3.28E-27 | 13 | Tk1 |
| 1.44566461752437e-30 | 2.032323 | 0.802 | 0.384 | 4.34E-27 | 13 | Nsun2 |
| 1.3516011029522e-27 | 2.018860 | 0.767 | 0.4 | 4.05E-24 | 13 | Eif4ebp1 |
| 6.2730040285387e-19 | 2.166416 | 0.669 | 0.322 | 1.88E-15 | 13 | Cfp |
| 2.03024612121824e-18 | 4.428434 | 0.698 | 0.175 | 6.09E-15 | 13 | Cxcl3 |
| 8.72157051699923e-17 | 3.391481 | 0.576 | 0.164 | 2.62E-13 | 13 | Cd163 |
| 3.09479167715464e-13 | 3.911160 | 0.698 | 0.353 | 9.28E-10 | 13 | Itgb2 |
| 7.549098050008e-65 | 3.414820 | 0.994 | 0.485 | 2.26E-61 | 14 | S100a4 |
| 4.61245250179643e-48 | 2.134866 | 0.964 | 0.458 | 1.38E-44 | 14 | Fgfr1 |
| 1.13174947387707e-39 | 3.528103 | 0.849 | 0.385 | 3.40E-36 | 14 | Apcdd1 |
| 3.63605010930267e-35 | 2.058473 | 0.867 | 0.45 | 1.09E-31 | 14 | Hist1h1e |
| 2.28542365233241e-33 | 2.498587 | 0.777 | 0.332 | 6.86E-30 | 14 | Hic1 |
| 2.34964222139517e-31 | 3.589500 | 0.795 | 0.343 | 7.05E-28 | 14 | Loxl3 |
| 3.93575219875977e-30 | 2.382964 | 0.837 | 0.401 | 1.18E-26 | 14 | Atp5c1 |
| 1.51679370024244e-25 | 2.279255 | 0.795 | 0.441 | 4.55E-22 | 14 | Atp5g3 |
| 2.27908091292886e-24 | 2.085125 | 0.777 | 0.395 | 6.84E-21 | 14 | Cdk4 |
| 1.4118643515882e-23 | 2.150544 | 0.753 | 0.365 | 4.24E-20 | 14 | Hist1h1a |
| 1.94401926500833e-23 | 2.081272 | 0.765 | 0.361 | 5.83E-20 | 14 | Rhou |
| 1.68127958475125e-13 | 2.034860 | 0.741 | 0.389 | 5.04E-10 | 14 | Cdk1 |
| 5.05282028884415e-12 | 2.292872 | 0.627 | 0.335 | 1.52E-08 | 14 | Gcnt1 |
| 2.88080294795159e-10 | 2.239835 | 0.657 | 0.349 | 8.64E-07 | 14 | Cse1l |
| 1.01344211960837e-46 | 6.290330 | 0.946 | 0.309 | 3.04E-43 | 15 | Col6a3 |
| 6.47555819673158e-45 | 7.264046 | 0.925 | 0.285 | 1.94E-41 | 15 | Lum |
| 1.9266276518958e-35 | 6.162159 | 0.849 | 0.208 | 5.78E-32 | 15 | Apod |
| 1.69584514075688e-34 | 7.116395 | 0.892 | 0.307 | 5.09E-31 | 15 | Lcn2 |
| 9.4668043037997e-33 | 5.355093 | 0.903 | 0.352 | 2.84E-29 | 15 | Apoe |
| 5.51008437292771e-31 | 2.852968 | 0.892 | 0.374 | 1.65E-27 | 15 | Prrx1 |
| 3.73186098993859e-29 | 5.695759 | 0.839 | 0.37 | 1.12E-25 | 15 | Npc2 |
| 4.03408283222287e-29 | 5.737128 | 0.839 | 0.32 | 1.21E-25 | 15 | Cxcl15 |
| 1.06822375463316e-26 | 4.088319 | 0.817 | 0.302 | 3.20E-23 | 15 | Chil1 |
| 3.71062341994179e-26 | 3.314587 | 0.839 | 0.348 | 1.11E-22 | 15 | Sftpa1 |
| 5.26909113516273e-26 | 2.547048 | 0.86 | 0.336 | 1.58E-22 | 15 | Slc34a2 |
| 1.64388483298212e-24 | 5.785652 | 0.806 | 0.289 | 4.93E-21 | 15 | Lrg1 |
| 3.58991926960992e-23 | 3.513154 | 0.817 | 0.36 | 1.08E-19 | 15 | Fbn1 |
| 1.25960267710953e-22 | 3.617368 | 0.796 | 0.378 | 3.78E-19 | 15 | Jun |
| 1.61524937716582e-22 | 4.662617 | 0.742 | 0.267 | 4.85E-19 | 15 | Sftpd |
| 4.64454634262555e-21 | 3.714288 | 0.839 | 0.387 | 1.39E-17 | 15 | Mmp14 |
| 1.50110869813773e-20 | 3.319802 | 0.796 | 0.272 | 4.50E-17 | 15 | Scd1 |
| 8.22055875434158e-20 | 2.377300 | 0.828 | 0.418 | 2.47E-16 | 15 | Lamb1 |
| 2.65689378353897e-19 | 2.009347 | 0.828 | 0.467 | 7.97E-16 | 15 | Itm2b |
| 2.9815102857418e-19 | 5.485142 | 0.71 | 0.264 | 8.94E-16 | 15 | Gpnmb |
| 1.00200931321179e-17 | 3.678368 | 0.731 | 0.217 | 3.01E-14 | 15 | S100g |
| 1.25877450868867e-16 | 3.038328 | 0.742 | 0.278 | 3.78E-13 | 15 | Mfap4 |
| 4.30843673381557e-16 | 2.297347 | 0.763 | 0.355 | 1.29E-12 | 15 | Ctss |
| 2.58636281383086e-15 | 5.400918 | 0.731 | 0.326 | 7.76E-12 | 15 | Tnc |
| 1.9965198900864e-14 | 2.203535 | 0.731 | 0.339 | 5.99E-11 | 15 | Prpf39 |
| 1.87704470164845e-13 | 3.176923 | 0.731 | 0.342 | 5.63E-10 | 15 | Rdh10 |
| 2.19435120511396e-13 | 2.042699 | 0.72 | 0.312 | 6.58E-10 | 15 | Elovl1 |
| 6.5282677145847e-13 | 3.909090 | 0.645 | 0.231 | 1.96E-09 | 15 | Fosb |
| 8.96926704792939e-13 | 2.124928 | 0.688 | 0.276 | 2.69E-09 | 15 | Osr2 |
| 5.39261518518295e-12 | 2.495817 | 0.742 | 0.382 | 1.62E-08 | 15 | Junb |
| 9.49101742415653e-11 | 2.917267 | 0.667 | 0.389 | 2.85E-07 | 15 | Sdc1 |
| 1.70301041313754e-10 | 2.762224 | 0.634 | 0.239 | 5.11E-07 | 15 | Irx1 |
| 2.87093101915777e-10 | 2.217439 | 0.688 | 0.344 | 8.61E-07 | 15 | Fasn |
| 5.62196538946911e-09 | 2.011807 | 0.699 | 0.371 | 1.69E-05 | 15 | Arl4c |
| 1.36620654933121e-08 | 2.344386 | 0.591 | 0.34 | 4.10E-05 | 15 | Per1 |
| 1.82022124169159e-08 | 3.586408 | 0.645 | 0.325 | 5.46E-05 | 15 | Egr1 |
| 4.08068807060092e-07 | 2.713034 | 0.602 | 0.261 | 1.22E-03 | 15 | Cd300a |
| 8.83654604867753e-07 | 2.782155 | 0.634 | 0.346 | 2.65E-03 | 15 | Kifap3 |
| 1.76943788425407e-06 | 2.149500 | 0.591 | 0.233 | 5.31E-03 | 15 | Igfbp2 |
| 3.81379949803005e-06 | 2.289848 | 0.194 | 0.175 | 1.14E-02 | 15 | Rftn2 |
| 4.73144206659184e-06 | 3.039314 | 0.14 | 0.133 | 1.42E-02 | 15 | Pdgfd |
| 1.35694486949091e-05 | 4.246891 | 0.591 | 0.301 | 4.07E-02 | 15 | Mmp12 |
| 1.43897171135207e-05 | 2.093402 | 0.613 | 0.405 | 4.32E-02 | 15 | Vat1 |
| 6.38744258613668e-68 | 2.160833 | 0.016 | 0.203 | 1.92E-64 | 2 | Sema6a |
| 8.54163227997244e-50 | 2.546970 | 0.031 | 0.241 | 2.56E-46 | 2 | Trim14 |
| 5.06327311035993e-49 | 3.352141 | 0.024 | 0.243 | 1.52E-45 | 2 | Gab2 |
| 5.35441927360033e-47 | 2.715887 | 0.147 | 0.543 | 1.61E-43 | 2 | Eef2 |
| 1.90787921665101e-44 | 3.171962 | 0.02 | 0.253 | 5.72E-41 | 2 | Sp4 |
| 2.73145284924385e-43 | 3.314213 | 0.013 | 0.255 | 8.19E-40 | 2 | Arrdc4 |
| 1.60887119256486e-42 | 2.504208 | 0.009 | 0.243 | 4.83E-39 | 2 | Ngef |
| 7.16598247087608e-41 | 2.848247 | 0.016 | 0.26 | 2.15E-37 | 2 | Dram2 |
| 5.10198809556373e-39 | 3.704648 | 0.027 | 0.267 | 1.53E-35 | 2 | Nxpe4 |
| 3.13720429345441e-37 | 2.098716 | 0.024 | 0.267 | 9.41E-34 | 2 | C2cd2 |
| 1.49039828438729e-36 | 3.435837 | 0.16 | 0.545 | 4.47E-33 | 2 | Hnrnpa0 |
| 1.15405663056466e-31 | 3.640728 | 0.252 | 0.271 | 3.46E-28 | 2 | Acsbg1 |
| 2.03719821864203e-30 | 2.567504 | 0.042 | 0.292 | 6.11E-27 | 2 | Acp5 |
| 7.97656565537606e-28 | 2.412758 | 0.04 | 0.303 | 2.39E-24 | 2 | Gch1 |
| 1.35964741705408e-26 | 2.610013 | 0.165 | 0.531 | 4.08E-23 | 2 | H3f3b |
| 5.24057887975695e-26 | 2.271669 | 0.038 | 0.305 | 1.57E-22 | 2 | Lama4 |
| 5.3288354201481e-26 | 3.324584 | 0.045 | 0.312 | 1.60E-22 | 2 | Tk2 |
| 1.76224928639301e-23 | 3.521845 | 0.414 | 0.315 | 5.29E-20 | 2 | Cd300c2 |
| 1.14957474045663e-22 | 3.611210 | 0.024 | 0.313 | 3.45E-19 | 2 | Lst1 |
| 2.97731825211156e-22 | 2.214539 | 0.042 | 0.312 | 8.93E-19 | 2 | Tanc2 |
| 1.03662983252165e-21 | 4.039204 | 0.036 | 0.317 | 3.11E-18 | 2 | Gstz1 |
| 3.10700979279588e-20 | 4.241724 | 0.321 | 0.323 | 9.32E-17 | 2 | Nfkbil1 |
| 1.173728138956e-19 | 2.174650 | 0.047 | 0.315 | 3.52E-16 | 2 | Birc3 |
| 1.78496762811859e-19 | 4.860553 | 0.428 | 0.336 | 5.35E-16 | 2 | Dagla |
| 4.81547490412542e-19 | 4.074373 | 0.04 | 0.327 | 1.44E-15 | 2 | Dyrk2 |
| 4.85204334937278e-18 | 2.361943 | 0.185 | 0.534 | 1.46E-14 | 2 | Tcp1 |
| 5.27128981953389e-18 | 3.487900 | 0.2 | 0.327 | 1.58E-14 | 2 | Olfm1 |
| 8.10453634301044e-18 | 4.417629 | 0.062 | 0.332 | 2.43E-14 | 2 | Rit1 |
| 1.18490439718761e-17 | 3.412557 | 0.238 | 0.507 | 3.55E-14 | 2 | Cnbp |
| 1.23906341198739e-17 | 3.022039 | 0.125 | 0.326 | 3.72E-14 | 2 | Gpatch2l |
| 2.78334252449017e-17 | 2.036165 | 0.036 | 0.33 | 8.35E-14 | 2 | Grb10 |
| 4.70759303234752e-17 | 2.858859 | 0.283 | 0.341 | 1.41E-13 | 2 | Ptpn14 |
| 5.91594291836348e-17 | 4.120951 | 0.096 | 0.332 | 1.77E-13 | 2 | Pvr |
| 6.37634704357491e-17 | 2.095068 | 0.252 | 0.524 | 1.91E-13 | 2 | Pkm |
| 6.4719608832247e-17 | 3.532057 | 0.058 | 0.329 | 1.94E-13 | 2 | Nectin1 |
| 1.11753271048764e-16 | 2.265302 | 0.154 | 0.343 | 3.35E-13 | 2 | Rtkn |
| 2.41255698518309e-16 | 2.463604 | 0.261 | 0.525 | 7.24E-13 | 2 | Eif4g2 |
| 4.38942760885188e-16 | 2.141171 | 0.027 | 0.33 | 1.32E-12 | 2 | Zfp395 |
| 8.78874939544844e-16 | 3.324891 | 0.031 | 0.333 | 2.64E-12 | 2 | Gsap |
| 9.03199510743032e-16 | 3.916015 | 0.12 | 0.347 | 2.71E-12 | 2 | Baz2b |
| 1.03670672177007e-15 | 2.914233 | 0.169 | 0.347 | 3.11E-12 | 2 | Man2b1 |
| 1.29828615641023e-15 | 4.711981 | 0.229 | 0.531 | 3.89E-12 | 2 | Hspa5 |
| 1.41565168449745e-15 | 2.942237 | 0.194 | 0.344 | 4.25E-12 | 2 | Spsb1 |
| 1.87862277438288e-15 | 4.567780 | 0.073 | 0.335 | 5.64E-12 | 2 | March2 |
| 2.17915109580937e-15 | 3.819893 | 0.045 | 0.331 | 6.54E-12 | 2 | Cblb |
| 2.29350556478603e-15 | 4.605418 | 0.109 | 0.485 | 6.88E-12 | 2 | Sqstm1 |
| 2.58407128635162e-15 | 2.898123 | 0.145 | 0.346 | 7.75E-12 | 2 | Trim16 |
| 6.40212960619051e-15 | 4.279290 | 0.1 | 0.35 | 1.92E-11 | 2 | Rdh14 |
| 7.47046913110056e-15 | 4.085337 | 0.171 | 0.345 | 2.24E-11 | 2 | Parp10 |
| 7.82251307772984e-15 | 2.948456 | 0.343 | 0.348 | 2.35E-11 | 2 | Mov10 |
| 1.36954183449757e-14 | 4.449141 | 0.069 | 0.346 | 4.11E-11 | 2 | Rap1gap |
| 2.0323508734897e-14 | 4.476456 | 0.194 | 0.518 | 6.10E-11 | 2 | Atp5a1 |
| 3.63302368247416e-14 | 5.794397 | 0.354 | 0.352 | 1.09E-10 | 2 | Prkar2b |
| 3.87038451689707e-14 | 3.103648 | 0.06 | 0.338 | 1.16E-10 | 2 | Taok3 |
| 3.94452049029781e-14 | 2.269778 | 0.058 | 0.351 | 1.18E-10 | 2 | Washc4 |
| 4.74255628855332e-14 | 3.563026 | 0.361 | 0.343 | 1.42E-10 | 2 | Vdac3 |
| 5.16353408775693e-14 | 3.719768 | 0.318 | 0.35 | 1.55E-10 | 2 | Gjc1 |
| 5.24617947039531e-14 | 3.268865 | 0.038 | 0.343 | 1.57E-10 | 2 | Atxn7l1 |
| 7.08691858731286e-14 | 5.007044 | 0.125 | 0.35 | 2.13E-10 | 2 | Slc14a1 |
| 9.3051425502567e-14 | 2.174751 | 0.049 | 0.329 | 2.79E-10 | 2 | Abca3 |
| 1.89340246884076e-13 | 3.185801 | 0.053 | 0.341 | 5.68E-10 | 2 | Helz2 |
| 2.45851938324805e-13 | 4.314224 | 0.143 | 0.351 | 7.38E-10 | 2 | Adarb1 |
| 3.56846646568278e-13 | 3.358981 | 0.089 | 0.347 | 1.07E-09 | 2 | Ifngr2 |
| 5.56524113167378e-13 | 2.010481 | 0.183 | 0.352 | 1.67E-09 | 2 | Pea15a |
| 6.02931699079569e-13 | 2.623539 | 0.051 | 0.343 | 1.81E-09 | 2 | Kdm3a |
| 7.09754449592697e-13 | 4.316970 | 0.051 | 0.341 | 2.13E-09 | 2 | 4932438A13Rik |
| 1.76999671721596e-12 | 2.320027 | 0.151 | 0.485 | 5.31E-09 | 2 | Sparc |
| 1.77213717837383e-12 | 4.231889 | 0.116 | 0.348 | 5.32E-09 | 2 | Tcta |
| 2.91853653742712e-12 | 4.404949 | 0.065 | 0.344 | 8.76E-09 | 2 | Tmem9b |
| 4.39474794297256e-12 | 4.845045 | 0.405 | 0.365 | 1.32E-08 | 2 | Kif11 |
| 5.26753067163396e-12 | 4.730909 | 0.281 | 0.362 | 1.58E-08 | 2 | C3ar1 |
| 7.16641094512222e-12 | 2.805533 | 0.067 | 0.351 | 2.15E-08 | 2 | Thoc2 |
| 1.59379256264168e-11 | 3.444565 | 0.234 | 0.349 | 4.78E-08 | 2 | Dennd5a |
| 1.78177141850449e-11 | 2.183536 | 0.08 | 0.361 | 5.35E-08 | 2 | Efemp1 |
| 2.82471123977301e-11 | 2.562746 | 0.18 | 0.359 | 8.47E-08 | 2 | Clk4 |
| 2.90255036967528e-11 | 2.690963 | 0.091 | 0.352 | 8.71E-08 | 2 | Cd82 |
| 3.26592162056605e-11 | 2.945205 | 0.129 | 0.358 | 9.80E-08 | 2 | Exoc4 |
| 3.38936259779768e-11 | 2.432020 | 0.149 | 0.373 | 1.02E-07 | 2 | Dusp9 |
| 3.68113035572335e-11 | 4.621404 | 0.125 | 0.366 | 1.10E-07 | 2 | Atp6ap2 |
| 5.92019624973810e-11 | 2.544378 | 0.229 | 0.504 | 1.78E-07 | 2 | Naca |
| 7.8167274988316e-11 | 3.785025 | 0.147 | 0.36 | 2.35E-07 | 2 | Carhsp1 |
| 9.78812084045597e-11 | 2.950540 | 0.076 | 0.364 | 2.94E-07 | 2 | Mtmr9 |
| 1.40180208558007e-10 | 4.317625 | 0.114 | 0.36 | 4.21E-07 | 2 | Srsf7 |
| 1.4997536169372e-10 | 3.124014 | 0.056 | 0.342 | 4.50E-07 | 2 | Lsp1 |
| 1.61738432589809e-10 | 2.043375 | 0.045 | 0.344 | 4.85E-07 | 2 | Map4k3 |
| 1.62055156151141e-10 | 2.560327 | 0.241 | 0.37 | 4.86E-07 | 2 | Slc20a1 |
| 2.23528892688454e-10 | 5.171037 | 0.158 | 0.348 | 6.71E-07 | 2 | Trim11 |
| 2.87472756352205e-10 | 3.045458 | 0.134 | 0.362 | 8.62E-07 | 2 | Sat1 |
| 3.26278587974953e-10 | 2.191094 | 0.107 | 0.361 | 9.79E-07 | 2 | Stk3 |
| 3.46158819896554e-10 | 2.727998 | 0.085 | 0.36 | 1.04E-06 | 2 | Eml2 |
| 3.86184793711297e-10 | 5.096741 | 0.143 | 0.483 | 1.16E-06 | 2 | Sem1 |
| 4.50985670924207e-10 | 2.039612 | 0.073 | 0.357 | 1.35E-06 | 2 | Plscr3 |
| 4.87048178650229e-10 | 3.263889 | 0.071 | 0.361 | 1.46E-06 | 2 | Ncoa4 |
| 7.64458866415123e-10 | 4.176518 | 0.127 | 0.365 | 2.29E-06 | 2 | Lmbrd1 |
| 9.24218464312973e-10 | 2.412374 | 0.111 | 0.37 | 2.77E-06 | 2 | Mri1 |
| 1.36207692290594e-09 | 3.287353 | 0.343 | 0.58 | 4.09E-06 | 2 | Rack1 |
| 1.57308293987444e-09 | 3.653882 | 0.08 | 0.365 | 4.72E-06 | 2 | Bloc1s6 |
| 1.75202165287366e-09 | 4.952284 | 0.109 | 0.374 | 5.26E-06 | 2 | Dock7 |
| 1.75911410623071e-09 | 3.290250 | 0.098 | 0.369 | 5.28E-06 | 2 | Pclaf |
| 2.65036388913709e-09 | 2.043640 | 0.111 | 0.366 | 7.95E-06 | 2 | Fcgrt |
| 2.99359351784262e-09 | 4.837551 | 0.205 | 0.359 | 8.98E-06 | 2 | Rbm33 |
| 3.29360184452323e-09 | 2.834158 | 0.14 | 0.382 | 9.88E-06 | 2 | Clint1 |
| 5.05662164477935e-09 | 4.999818 | 0.085 | 0.37 | 1.52E-05 | 2 | Rprd2 |
| 5.17779093312612e-09 | 4.123447 | 0.076 | 0.36 | 1.55E-05 | 2 | Marf1 |
| 5.94042564533201e-09 | 4.898702 | 0.134 | 0.37 | 1.78E-05 | 2 | Mapk12 |
| 7.47477719303832e-09 | 3.315604 | 0.203 | 0.492 | 2.24E-05 | 2 | Pcolce |
| 8.13505734765618e-09 | 4.851419 | 0.08 | 0.364 | 2.44E-05 | 2 | Bahcc1 |
| 9.32984033339142e-09 | 3.481783 | 0.067 | 0.363 | 2.80E-05 | 2 | Samhd1 |
| 1.34170889035346e-08 | 3.290185 | 0.078 | 0.371 | 4.03E-05 | 2 | Cdc42se1 |
| 2.14265400700099e-08 | 3.619843 | 0.078 | 0.353 | 6.43E-05 | 2 | Scarb2 |
| 2.17992141463218e-08 | 3.426016 | 0.058 | 0.364 | 6.54E-05 | 2 | Mtmr6 |
| 2.25101677503334e-08 | 2.028500 | 0.205 | 0.496 | 6.75E-05 | 2 | Gja1 |
| 2.33337571171218e-08 | 4.084665 | 0.096 | 0.361 | 7.00E-05 | 2 | Snx18 |
| 2.38178027324491e-08 | 2.812096 | 0.069 | 0.367 | 7.15E-05 | 2 | Zmym2 |
| 2.47169399393773e-08 | 2.600773 | 0.107 | 0.369 | 7.42E-05 | 2 | Tmem106b |
| 2.51693215215026e-08 | 3.921480 | 0.078 | 0.368 | 7.55E-05 | 2 | Sppl2b |
| 2.57012889907191e-08 | 4.524433 | 0.067 | 0.364 | 7.71E-05 | 2 | Cabin1 |
| 3.44932372265054e-08 | 3.446003 | 0.071 | 0.369 | 1.03E-04 | 2 | R3hdm4 |
| 3.48284355893727e-08 | 5.167530 | 0.089 | 0.373 | 1.04E-04 | 2 | Ppp2r5e |
| 3.81077997464792e-08 | 2.936143 | 0.165 | 0.399 | 1.14E-04 | 2 | Bax |
| 4.11255472263102e-08 | 3.778183 | 0.107 | 0.385 | 1.23E-04 | 2 | Socs6 |
| 4.25534641419707e-08 | 3.109822 | 0.08 | 0.367 | 1.28E-04 | 2 | Gigyf1 |
| 4.41503401152758e-08 | 2.712741 | 0.131 | 0.387 | 1.32E-04 | 2 | Pafah1b3 |
| 6.60022770772082e-08 | 4.989784 | 0.094 | 0.369 | 1.98E-04 | 2 | Scara3 |
| 1.24383760331658e-07 | 2.382173 | 0.1 | 0.376 | 3.73E-04 | 2 | Tiam2 |
| 1.40136035623706e-07 | 3.591515 | 0.281 | 0.492 | 4.20E-04 | 2 | Eif3f |
| 1.45625548375939e-07 | 2.201297 | 0.138 | 0.376 | 4.37E-04 | 2 | Acaa2 |
| 2.64623972101331e-07 | 3.016230 | 0.111 | 0.382 | 7.94E-04 | 2 | Gli3 |
| 2.95402055764426e-07 | 3.100892 | 0.136 | 0.399 | 8.86E-04 | 2 | Mmadhc |
| 4.00053525055791e-07 | 2.072057 | 0.089 | 0.468 | 1.20E-03 | 2 | Pa2g4 |
| 6.03212777442503e-07 | 3.993649 | 0.218 | 0.494 | 1.81E-03 | 2 | Clic1 |
| 6.76715101505724e-07 | 4.202533 | 0.06 | 0.369 | 2.03E-03 | 2 | Pfkp |
| 6.909392822948e-07 | 4.640306 | 0.212 | 0.386 | 2.07E-03 | 2 | Prc1 |
| 7.05179917373505e-07 | 2.988955 | 0.085 | 0.375 | 2.12E-03 | 2 | Sdc2 |
| 7.23587383022538e-07 | 4.128714 | 0.105 | 0.367 | 2.17E-03 | 2 | Hmgb1 |
| 8.49158652744858e-07 | 2.641054 | 0.091 | 0.374 | 2.55E-03 | 2 | Eapp |
| 9.12674112537468e-07 | 2.030004 | 0.1 | 0.385 | 2.74E-03 | 2 | Sntb2 |
| 1.28044822594363e-06 | 2.701241 | 0.102 | 0.374 | 3.84E-03 | 2 | Spry2 |
| 1.48405923090648e-06 | 3.263796 | 0.085 | 0.377 | 4.45E-03 | 2 | Tpp1 |
| 1.72662480323786e-06 | 2.439258 | 0.134 | 0.397 | 5.18E-03 | 2 | Kcnab2 |
| 1.74713474599509e-06 | 3.281838 | 0.118 | 0.472 | 5.24E-03 | 2 | Col6a1 |
| 1.94616704694297e-06 | 4.724808 | 0.129 | 0.387 | 5.84E-03 | 2 | Fhl2 |
| 1.97045202511564e-06 | 5.039406 | 0.078 | 0.375 | 5.91E-03 | 2 | Wnt7b |
| 2.10892115545826e-06 | 4.927612 | 0.111 | 0.377 | 6.33E-03 | 2 | Krt8 |
| 2.25504954470984e-06 | 3.555504 | 0.091 | 0.376 | 6.77E-03 | 2 | Mogs |
| 2.84554115243265e-06 | 2.099914 | 0.069 | 0.367 | 8.54E-03 | 2 | Coro1a |
| 3.87005230117423e-06 | 2.689887 | 0.156 | 0.489 | 1.16E-02 | 2 | Hspa9 |
| 4.23964700394639e-06 | 4.510393 | 0.094 | 0.375 | 1.27E-02 | 2 | Xbp1 |
| 4.90752079234628e-06 | 5.370012 | 0.127 | 0.395 | 1.47E-02 | 2 | Itpr3 |
| 5.00244592557459e-06 | 3.624925 | 0.278 | 0.388 | 1.50E-02 | 2 | Tfrc |
| 5.1123055137781e-06 | 2.230794 | 0.1 | 0.378 | 1.53E-02 | 2 | Arhgef1 |
| 7.47163167767486e-06 | 2.778734 | 0.127 | 0.394 | 2.24E-02 | 2 | Ttc3 |
| 8.30523351542146e-06 | 4.396804 | 0.12 | 0.389 | 2.49E-02 | 2 | Ndufb6 |
| 8.52879311390449e-06 | 4.257840 | 0.042 | 0.367 | 2.56E-02 | 2 | Tgfbi |
| 9.00344443544187e-06 | 2.008921 | 0.089 | 0.388 | 2.70E-02 | 2 | Ankrd10 |
| 9.00813655650275e-06 | 4.687541 | 0.158 | 0.403 | 2.70E-02 | 2 | Aebp2 |
| 9.03241395338005e-06 | 2.547958 | 0.056 | 0.371 | 2.71E-02 | 2 | Wars |
| 9.70784940996435e-06 | 5.138558 | 0.151 | 0.402 | 2.91E-02 | 2 | Rgmb |
| 1.10464094077959e-05 | 2.467291 | 0.134 | 0.393 | 3.31E-02 | 2 | Mapkapk2 |
| 1.34975889958091e-05 | 4.504350 | 0.102 | 0.384 | 4.05E-02 | 2 | Gnl2 |
| 1.63916938225641e-05 | 3.302922 | 0.149 | 0.408 | 4.92E-02 | 2 | Gusb |
| 2.21305555687744e-86 | 2.569945 | 0.834 | 0.387 | 6.64E-83 | 3 | Ank |
| 6.3090634866023e-66 | 2.448124 | 0.742 | 0.324 | 1.89E-62 | 3 | Dcn |
| 1.85355955211535e-48 | 2.187793 | 0.754 | 0.393 | 5.56E-45 | 3 | Col18a1 |
| 6.54896953908495e-26 | 2.822186 | 0.635 | 0.317 | 1.96E-22 | 3 | Cd248 |
| 6.86975807173049e-25 | 2.154641 | 0.635 | 0.316 | 2.06E-21 | 3 | Vasn |
| 1.10540348225211e-15 | 2.870330 | 0.213 | 0.239 | 3.32E-12 | 3 | Lfng |
| 7.46306033288112e-10 | 4.063039 | 0.261 | 0.247 | 2.24E-06 | 3 | Btbd3 |
| 5.41824284259607e-08 | 2.009530 | 0.561 | 0.33 | 1.63E-04 | 3 | Nsa2 |
| 8.23864489317841e-129 | 3.119745 | 0.965 | 0.425 | 2.47E-125 | 4 | Igfbp4 |
| 5.34816350173e-128 | 2.424247 | 1 | 0.529 | 1.60E-124 | 4 | Eef1a1 |
| 2.75252797898153e-109 | 2.384516 | 0.956 | 0.394 | 8.26E-106 | 4 | Col3a1 |
| 1.89059939646453e-107 | 3.075726 | 0.897 | 0.342 | 5.67E-104 | 4 | Dpt |
| 2.05663836135191e-99 | 2.150911 | 0.962 | 0.477 | 6.17E-96 | 4 | Nedd4 |
| 7.95828471082748e-79 | 2.176988 | 0.865 | 0.385 | 2.39E-75 | 4 | Zcchc24 |
| 9.34978977762715e-62 | 2.562156 | 0.812 | 0.395 | 2.80E-58 | 4 | Ank |
| 1.26379842206242e-60 | 2.603575 | 0.829 | 0.431 | 3.79E-57 | 4 | Spp1 |
| 2.9918112309123e-59 | 2.316929 | 0.803 | 0.354 | 8.98E-56 | 4 | Il1r1 |
| 1.07680762196228e-58 | 2.854821 | 0.794 | 0.325 | 3.23E-55 | 4 | F13a1 |
| 1.08511001313836e-57 | 2.510597 | 0.785 | 0.321 | 3.26E-54 | 4 | Nabp1 |
| 6.53721742760606e-56 | 2.242816 | 0.75 | 0.315 | 1.96E-52 | 4 | Spry2 |
| 2.13182592342329e-46 | 2.437667 | 0.724 | 0.331 | 6.40E-43 | 4 | Stab1 |
| 1.74795606858838e-44 | 2.040718 | 0.729 | 0.347 | 5.24E-41 | 4 | Bmp1 |
| 6.44940153408362e-44 | 2.031479 | 0.738 | 0.332 | 1.93E-40 | 4 | Idh1 |
| 4.62546276311602e-42 | 2.432288 | 0.724 | 0.318 | 1.39E-38 | 4 | Hic1 |
| 2.30769887611807e-39 | 2.038193 | 0.671 | 0.338 | 6.92E-36 | 4 | Dkk2 |
| 6.16507627347805e-37 | 2.908269 | 0.8 | 0.477 | 1.85E-33 | 4 | Ctsl |
| 9.2872518631396e-37 | 2.238267 | 0.703 | 0.341 | 2.79E-33 | 4 | Lrig1 |
| 8.30487596048253e-35 | 6.125551 | 0.629 | 0.197 | 2.49E-31 | 4 | Ccl11 |
| 1.52166211732998e-22 | 2.909598 | 0.609 | 0.258 | 4.56E-19 | 4 | Osr2 |
| 6.06477495852171e-17 | 2.234138 | 0.597 | 0.298 | 1.82E-13 | 4 | Adamts1 |
| 1.54222205865693e-10 | 2.645154 | 0.262 | 0.195 | 4.63E-07 | 4 | Slc40a1 |
| 3.26891413344956e-112 | 2.896384 | 0.99 | 0.463 | 9.81E-109 | 5 | Pkm |
| 1.04561333339667e-106 | 4.096100 | 0.953 | 0.399 | 3.14E-103 | 5 | Mif |
| 5.00175361180699e-102 | 4.361618 | 0.927 | 0.383 | 1.50E-98 | 5 | Myc |
| 4.9922120661958e-101 | 3.359025 | 0.92 | 0.381 | 1.50E-97 | 5 | Thbs1 |
| 5.42432365573946e-95 | 2.384652 | 0.99 | 0.528 | 1.63E-91 | 5 | Vim |
| 4.07889099728596e-91 | 3.607719 | 0.92 | 0.402 | 1.22E-87 | 5 | Pgam1 |
| 6.179322657306e-90 | 2.695630 | 0.953 | 0.495 | 1.85E-86 | 5 | Ncl |
| 1.52128385647091e-85 | 3.958019 | 0.867 | 0.433 | 4.56E-82 | 5 | Hist1h1d |
| 1.7242037720269e-84 | 3.912537 | 0.904 | 0.406 | 5.17E-81 | 5 | Mt2 |
| 3.48774777797467e-84 | 4.293860 | 0.857 | 0.321 | 1.05E-80 | 5 | Serpine1 |
| 2.27061228551514e-83 | 2.679901 | 0.93 | 0.512 | 6.81E-80 | 5 | Cfl1 |
| 2.79646189902662e-83 | 2.525127 | 0.957 | 0.504 | 8.39E-80 | 5 | Txn1 |
| 9.09509677573653e-83 | 3.517193 | 0.93 | 0.443 | 2.73E-79 | 5 | Gpi1 |
| 1.85204881071354e-81 | 4.406990 | 0.9 | 0.476 | 5.56E-78 | 5 | Tagln2 |
| 1.78920314301911e-75 | 3.436658 | 0.88 | 0.438 | 5.37E-72 | 5 | Gja1 |
| 8.32270892464689e-75 | 3.546112 | 0.86 | 0.443 | 2.50E-71 | 5 | Hist2h2bb |
| 2.64283486479951e-72 | 2.035765 | 0.95 | 0.522 | 7.93E-69 | 5 | Pabpc1 |
| 1.08168842902605e-70 | 4.111613 | 0.831 | 0.383 | 3.25E-67 | 5 | Mt1 |
| 2.29218403098479e-70 | 3.538090 | 0.86 | 0.428 | 6.88E-67 | 5 | Hist1h3c |
| 7.06409891126727e-70 | 2.213444 | 0.847 | 0.38 | 2.12E-66 | 5 | Tubb6 |
| 1.18300334563359e-69 | 3.595353 | 0.85 | 0.345 | 3.55E-66 | 5 | Hmox1 |
| 1.17666826774631e-68 | 3.813351 | 0.89 | 0.415 | 3.53E-65 | 5 | Hist1h2bb |
| 1.38200471081804e-66 | 2.654564 | 0.87 | 0.463 | 4.15E-63 | 5 | Lmna |
| 4.76923546894847e-65 | 2.602975 | 0.847 | 0.393 | 1.43E-61 | 5 | Nop56 |
| 1.76453716064659e-64 | 2.010942 | 0.88 | 0.473 | 5.29E-61 | 5 | Tcp1 |
| 7.90714918075197e-64 | 2.137149 | 0.854 | 0.445 | 2.37E-60 | 5 | Anp32b |
| 2.29193251745674e-62 | 2.323498 | 0.841 | 0.438 | 6.88E-59 | 5 | Hist1h1e |
| 3.80847087395367e-62 | 2.174095 | 0.867 | 0.452 | 1.14E-58 | 5 | Npm1 |
| 4.08251063734528e-61 | 3.595475 | 0.824 | 0.371 | 1.22E-57 | 5 | Pgk1 |
| 9.72258219826029e-61 | 2.276621 | 0.854 | 0.478 | 2.92E-57 | 5 | Lgals1 |
| 1.95074251462346e-60 | 2.389218 | 0.874 | 0.471 | 5.85E-57 | 5 | Ybx3 |
| 4.27736801038682e-60 | 2.423660 | 0.864 | 0.451 | 1.28E-56 | 5 | Aldoa |
| 5.58484806412603e-60 | 2.332942 | 0.854 | 0.442 | 1.68E-56 | 5 | Rhox5 |
| 7.88557598274718e-58 | 2.081857 | 0.87 | 0.5 | 2.37E-54 | 5 | Hist1h1c |
| 4.62744199092397e-57 | 3.425649 | 0.827 | 0.403 | 1.39E-53 | 5 | Hist1h3b |
| 4.7287207077043e-54 | 4.971254 | 0.784 | 0.345 | 1.42E-50 | 5 | Egln3 |
| 1.54365550125367e-53 | 2.479757 | 0.807 | 0.403 | 4.63E-50 | 5 | Hspd1 |
| 2.39808347395588e-53 | 3.024469 | 0.821 | 0.372 | 7.19E-50 | 5 | Cdk1 |
| 1.99300110567158e-51 | 3.068587 | 0.774 | 0.363 | 5.98E-48 | 5 | Psat1 |
| 1.40421123349595e-50 | 2.412284 | 0.794 | 0.459 | 4.21E-47 | 5 | Hnrnpab |
| 3.84823550430582e-49 | 2.489127 | 0.797 | 0.367 | 1.15E-45 | 5 | Strap |
| 4.58516689333639e-49 | 2.365881 | 0.791 | 0.372 | 1.38E-45 | 5 | Nsun2 |
| 7.10341981407185e-49 | 2.292927 | 0.817 | 0.442 | 2.13E-45 | 5 | Hdgf |
| 1.23629697520829e-48 | 2.341841 | 0.794 | 0.345 | 3.71E-45 | 5 | Tk1 |
| 2.14512466361539e-48 | 5.129211 | 0.757 | 0.331 | 6.44E-45 | 5 | Slc2a1 |
| 1.11049077471164e-47 | 2.450458 | 0.794 | 0.41 | 3.33E-44 | 5 | Hist1h3d |
| 2.59503632383318e-47 | 2.795646 | 0.767 | 0.366 | 7.79E-44 | 5 | Esd |
| 8.31360930896094e-44 | 2.663387 | 0.794 | 0.382 | 2.49E-40 | 5 | Hist2h2ac |
| 2.60676822755943e-43 | 2.342665 | 0.767 | 0.347 | 7.82E-40 | 5 | H2afx |
| 2.47955195232789e-42 | 2.233058 | 0.761 | 0.403 | 7.44E-39 | 5 | Cct5 |
| 4.17347211426122e-42 | 4.229354 | 0.781 | 0.352 | 1.25E-38 | 5 | Ccn1 |
| 5.22210432282969e-41 | 4.425984 | 0.734 | 0.316 | 1.57E-37 | 5 | Ndrg1 |
| 2.0414533595366e-40 | 2.379549 | 0.734 | 0.373 | 6.12E-37 | 5 | Hist1h2bj |
| 6.77529064772572e-40 | 2.504687 | 0.741 | 0.376 | 2.03E-36 | 5 | Gpc1 |
| 8.72797002358432e-40 | 2.652600 | 0.774 | 0.406 | 2.62E-36 | 5 | Sdc4 |
| 2.26992329908313e-39 | 2.069549 | 0.731 | 0.371 | 6.81E-36 | 5 | Mki67 |
| 3.20774339644705e-39 | 2.018923 | 0.764 | 0.406 | 9.62E-36 | 5 | Vars |
| 2.04403131522319e-38 | 2.171688 | 0.754 | 0.387 | 6.13E-35 | 5 | Uck2 |
| 4.48970061315464e-36 | 2.407348 | 0.714 | 0.343 | 1.35E-32 | 5 | Mthfd1l |
| 6.32985653274086e-36 | 2.164105 | 0.731 | 0.362 | 1.90E-32 | 5 | Hmga2 |
| 3.45609836101487e-35 | 2.465745 | 0.734 | 0.346 | 1.04E-31 | 5 | Mcm5 |
| 7.75593329817162e-34 | 2.253916 | 0.728 | 0.334 | 2.33E-30 | 5 | Pdk1 |
| 1.14882157728043e-32 | 2.291843 | 0.724 | 0.363 | 3.45E-29 | 5 | Ddx39 |
| 3.62767074116411e-31 | 2.337971 | 0.698 | 0.293 | 1.09E-27 | 5 | Hist1h2bg |
| 6.52358091366013e-31 | 5.358574 | 0.658 | 0.251 | 1.96E-27 | 5 | Angpt2 |
| 7.21316441697335e-30 | 4.546611 | 0.691 | 0.292 | 2.16E-26 | 5 | Ero1l |
| 1.72439176081745e-28 | 3.992035 | 0.684 | 0.321 | 5.17E-25 | 5 | Igfbp3 |
| 6.66344446988444e-28 | 2.812608 | 0.555 | 0.169 | 2.00E-24 | 5 | Cxcl3 |
| 5.96611805635208e-27 | 4.661872 | 0.661 | 0.357 | 1.79E-23 | 5 | Ier3 |
| 2.23511765482987e-26 | 2.114047 | 0.678 | 0.341 | 6.71E-23 | 5 | Cdt1 |
| 3.21829784899429e-25 | 3.529717 | 0.664 | 0.326 | 9.65E-22 | 5 | Egln1 |
| 4.15771907514473e-25 | 2.087009 | 0.671 | 0.346 | 1.25E-21 | 5 | Ccnb2 |
| 1.0634208056603e-24 | 2.170908 | 0.698 | 0.394 | 3.19E-21 | 5 | Eif4ebp1 |
| 2.47555102853858e-23 | 2.867406 | 0.688 | 0.348 | 7.43E-20 | 5 | Plau |
| 4.08450515786715e-23 | 2.538428 | 0.694 | 0.386 | 1.23E-19 | 5 | Ccna2 |
| 9.65078741808442e-23 | 4.121932 | 0.645 | 0.286 | 2.90E-19 | 5 | Adm |
| 2.09534912698713e-22 | 4.530529 | 0.641 | 0.318 | 6.29E-19 | 5 | Dusp4 |
| 4.55048106677421e-22 | 3.805292 | 0.645 | 0.269 | 1.37E-18 | 5 | Ptgs2 |
| 7.21034392258444e-22 | 3.026149 | 0.648 | 0.325 | 2.16E-18 | 5 | Sox9 |
| 8.00349053696735e-21 | 3.443830 | 0.625 | 0.27 | 2.40E-17 | 5 | Rbp4 |
| 7.60997085832018e-20 | 2.975748 | 0.641 | 0.313 | 2.28E-16 | 5 | Areg |
| 7.19286384334796e-18 | 2.050814 | 0.671 | 0.383 | 2.16E-14 | 5 | Pdap1 |
| 4.33088652777112e-17 | 2.278840 | 0.625 | 0.338 | 1.30E-13 | 5 | Ccbe1 |
| 5.19467593466826e-17 | 2.719415 | 0.635 | 0.346 | 1.56E-13 | 5 | Srsf2 |
| 1.06370878590933e-16 | 2.332673 | 0.635 | 0.362 | 3.19E-13 | 5 | Plaur |
| 6.99425391037643e-16 | 4.128833 | 0.229 | 0.237 | 2.10E-12 | 5 | Gbp3 |
| 9.86529890020858e-16 | 2.592002 | 0.621 | 0.361 | 2.96E-12 | 5 | Ncs1 |
| 2.30632409262884e-15 | 5.023279 | 0.588 | 0.211 | 6.92E-12 | 5 | Prss22 |
| 4.47581119195616e-15 | 4.043799 | 0.578 | 0.216 | 1.34E-11 | 5 | Rgs16 |
| 3.0328561565746e-14 | 5.218965 | 0.598 | 0.266 | 9.10E-11 | 5 | Hilpda |
| 1.14237465331501e-13 | 2.025042 | 0.628 | 0.356 | 3.43E-10 | 5 | Il1rl1 |
| 2.44881766916852e-13 | 2.066011 | 0.598 | 0.347 | 7.35E-10 | 5 | Tcim |
| 1.04050072365703e-11 | 4.903777 | 0.585 | 0.292 | 3.12E-08 | 5 | Dusp5 |
| 8.59629489428207e-10 | 2.529441 | 0.601 | 0.373 | 2.58E-06 | 5 | Abcc1 |
| 1.04972706337645e-09 | 4.843577 | 0.568 | 0.274 | 3.15E-06 | 5 | Hbegf |
| 4.51417353726107e-08 | 2.688593 | 0.551 | 0.34 | 1.35E-04 | 5 | Dnajb1 |
| 1.87457918050573e-07 | 5.124011 | 0.528 | 0.246 | 5.62E-04 | 5 | Hspa1b |
| 1.90105011323187e-07 | 3.097162 | 0.276 | 0.254 | 5.70E-04 | 5 | Gbp2 |
| 1.94213889807273e-07 | 2.538894 | 0.538 | 0.326 | 5.83E-04 | 5 | Hsph1 |
| 2.32791126341473e-06 | 2.470137 | 0.561 | 0.346 | 6.98E-03 | 5 | Hk2 |
| 4.26443411660498e-66 | 3.616167 | 0.136 | 0.054 | 1.28E-62 | 6 | Hspb7 |
| 6.39000682564091e-58 | 4.728976 | 0.146 | 0.068 | 1.92E-54 | 6 | Tcap |
| 2.84078401488288e-56 | 5.292765 | 0.129 | 0.08 | 8.52E-53 | 6 | Csrp3 |
| 6.83470104406177e-51 | 5.166962 | 0.213 | 0.119 | 2.05E-47 | 6 | Myh6 |
| 9.30688349893698e-50 | 4.825943 | 0.209 | 0.102 | 2.79E-46 | 6 | Tnnt2 |
| 7.04470599583694e-48 | 2.922845 | 0.111 | 0.097 | 2.11E-44 | 6 | Iglc1 |
| 2.12060517204754e-47 | 6.068911 | 0.16 | 0.126 | 6.36E-44 | 6 | Myl4 |
| 2.29327692536091e-47 | 6.065462 | 0.146 | 0.141 | 6.88E-44 | 6 | Cox6a2 |
| 2.29786212988276e-47 | 6.192985 | 0.153 | 0.078 | 6.89E-44 | 6 | Myl7 |
| 1.24784860685098e-40 | 2.357489 | 0.564 | 0.278 | 3.74E-37 | 6 | Igkc |
| 1.04323996020826e-34 | 3.602892 | 0.045 | 0.13 | 3.13E-31 | 6 | Dsp |
| 1.9285111100004e-33 | 2.257252 | 0.195 | 0.179 | 5.79E-30 | 6 | Cpxm1 |
| 8.86474603786298e-32 | 3.533417 | 0.08 | 0.133 | 2.66E-28 | 6 | Cd3d |
| 1.62250131924266e-31 | 3.066759 | 0.08 | 0.148 | 4.87E-28 | 6 | Ccr7 |
| 2.63092003292159e-31 | 3.717819 | 0.084 | 0.148 | 7.89E-28 | 6 | Tmem163 |
| 5.84308393729453e-29 | 5.026364 | 0.084 | 0.162 | 1.75E-25 | 6 | Trbc2 |
| 4.20677748845206e-28 | 2.627776 | 0.084 | 0.149 | 1.26E-24 | 6 | Tnxb |
| 1.46919073224734e-27 | 2.460787 | 0.279 | 0.242 | 4.41E-24 | 6 | Serpina3n |
| 3.08683125193646e-27 | 2.824265 | 0.056 | 0.154 | 9.26E-24 | 6 | Satb1 |
| 4.2193989723598e-27 | 2.410655 | 0.045 | 0.146 | 1.27E-23 | 6 | AA986860 |
| 2.16451119101763e-26 | 3.515836 | 0.066 | 0.158 | 6.49E-23 | 6 | Trac |
| 2.45893943160368e-26 | 4.503942 | 0.094 | 0.162 | 7.38E-23 | 6 | S1pr4 |
| 1.28132743929757e-25 | 2.541983 | 0.045 | 0.146 | 3.84E-22 | 6 | Angpt1 |
| 6.19029207462183e-25 | 2.438986 | 0.105 | 0.181 | 1.86E-21 | 6 | Tbx5 |
| 1.63417750407525e-24 | 2.451020 | 0.338 | 0.244 | 4.90E-21 | 6 | Ighm |
| 3.71125242536944e-24 | 2.194620 | 0.091 | 0.175 | 1.11E-20 | 6 | Dpp4 |
| 5.42187221158516e-24 | 3.669278 | 0.188 | 0.204 | 1.63E-20 | 6 | Igha |
| 1.12369435528692e-23 | 2.699453 | 0.042 | 0.153 | 3.37E-20 | 6 | Arvcf |
| 4.80639354302141e-22 | 4.482302 | 0.122 | 0.198 | 1.44E-18 | 6 | Icosl |
| 7.13874576419882e-21 | 2.813117 | 0.153 | 0.216 | 2.14E-17 | 6 | Gimap8 |
| 1.03994691933936e-20 | 2.047737 | 0.052 | 0.17 | 3.12E-17 | 6 | Egflam |
| 1.32328861897166e-20 | 3.155562 | 0.07 | 0.18 | 3.97E-17 | 6 | C6 |
| 2.57921658182779e-20 | 2.874835 | 0.052 | 0.151 | 7.74E-17 | 6 | Smad6 |
| 7.96953053137324e-20 | 2.462608 | 0.038 | 0.171 | 2.39E-16 | 6 | Col4a4 |
| 2.54183394466935e-19 | 2.673411 | 0.063 | 0.18 | 7.63E-16 | 6 | Col4a3 |
| 2.92087925912518e-19 | 2.815891 | 0.087 | 0.202 | 8.76E-16 | 6 | Arhgef6 |
| 3.18369820078263e-19 | 2.058873 | 0.098 | 0.196 | 9.55E-16 | 6 | Angptl7 |
| 5.57090571080334e-19 | 3.374940 | 0.056 | 0.182 | 1.67E-15 | 6 | Ndnf |
| 4.76499868934492e-18 | 3.507972 | 0.049 | 0.172 | 1.43E-14 | 6 | Rtn4rl2 |
| 1.10241428881086e-17 | 3.566329 | 0.031 | 0.174 | 3.31E-14 | 6 | Slc7a2 |
| 1.44541559084482e-17 | 2.073035 | 0.063 | 0.196 | 4.34E-14 | 6 | Nup210 |
| 1.53304176320785e-17 | 4.172511 | 0.077 | 0.202 | 4.60E-14 | 6 | Pmaip1 |
| 2.17551685953881e-17 | 3.466390 | 0.544 | 0.382 | 6.53E-14 | 6 | Cd74 |
| 3.24288003696166e-17 | 3.058891 | 0.077 | 0.202 | 9.73E-14 | 6 | Stim1 |
| 6.19989269641361e-17 | 3.248278 | 0.432 | 0.337 | 1.86E-13 | 6 | H2-Aa |
| 1.04939573536542e-16 | 3.347775 | 0.091 | 0.215 | 3.15E-13 | 6 | Trim30a |
| 1.33130410904659e-16 | 3.063370 | 0.157 | 0.239 | 3.99E-13 | 6 | Igfbp5 |
| 1.43496146561471e-16 | 2.579587 | 0.108 | 0.222 | 4.30E-13 | 6 | Zhx3 |
| 1.5795604565323e-16 | 3.393457 | 0.087 | 0.22 | 4.74E-13 | 6 | Cers4 |
| 2.43620438438833e-16 | 2.524441 | 0.087 | 0.221 | 7.31E-13 | 6 | Kctd6 |
| 3.94339638398504e-16 | 2.820704 | 0.063 | 0.209 | 1.18E-12 | 6 | Dmpk |
| 1.14904342231749e-15 | 3.236530 | 0.146 | 0.234 | 3.45E-12 | 6 | Itih4 |
| 1.30760879117516e-15 | 2.149633 | 0.077 | 0.189 | 3.92E-12 | 6 | Ccdc80 |
| 2.59538075148886e-15 | 4.197033 | 0.181 | 0.253 | 7.79E-12 | 6 | Ccl8 |
| 2.98925329847248e-15 | 2.749095 | 0.084 | 0.202 | 8.97E-12 | 6 | Phactr2 |
| 2.99035373167443e-15 | 2.088542 | 0.321 | 0.517 | 8.97E-12 | 6 | Ywhaz |
| 4.6334728894852e-15 | 3.929394 | 0.07 | 0.217 | 1.39E-11 | 6 | Hdc |
| 1.94960970138121e-14 | 2.483340 | 0.101 | 0.223 | 5.85E-11 | 6 | Fabp4 |
| 3.42263226586288e-14 | 2.377931 | 0.066 | 0.222 | 1.03E-10 | 6 | Sema4a |
| 1.15614162709688e-13 | 3.320342 | 0.105 | 0.228 | 3.47E-10 | 6 | Trim14 |
| 1.22410053318021e-13 | 2.094520 | 0.07 | 0.217 | 3.67E-10 | 6 | Tmem44 |
| 2.29812228625578e-13 | 3.738779 | 0.066 | 0.218 | 6.89E-10 | 6 | Lrrc32 |
| 5.2793538530558e-13 | 2.435060 | 0.105 | 0.242 | 1.58E-09 | 6 | Tmem98 |
| 8.50064028108275e-13 | 2.434402 | 0.568 | 0.357 | 2.55E-09 | 6 | Tmsb4x |
| 1.55157755518784e-12 | 2.891294 | 0.084 | 0.213 | 4.65E-09 | 6 | Gstt1 |
| 1.95926367871586e-12 | 2.102942 | 0.139 | 0.247 | 5.88E-09 | 6 | Igfbp2 |
| 2.59762134288433e-12 | 2.035105 | 0.066 | 0.225 | 7.79E-09 | 6 | Tacstd2 |
| 3.85000178749075e-12 | 2.502367 | 0.063 | 0.217 | 1.16E-08 | 6 | Rnf128 |
| 9.67798756095958e-12 | 3.453682 | 0.111 | 0.246 | 2.90E-08 | 6 | Htra3 |
| 2.6399233330105e-11 | 2.726406 | 0.08 | 0.235 | 7.92E-08 | 6 | Bcar3 |
| 4.89826146666822e-11 | 2.892485 | 0.272 | 0.297 | 1.47E-07 | 6 | H2-Eb1 |
| 5.75772259454846e-11 | 3.452244 | 0.101 | 0.243 | 1.73E-07 | 6 | Hvcn1 |
| 1.33414721957111e-10 | 3.307765 | 0.125 | 0.254 | 4.00E-07 | 6 | Ccl5 |
| 1.37268143302819e-10 | 3.746236 | 0.328 | 0.313 | 4.12E-07 | 6 | H2-Ab1 |
| 1.75894502887735e-10 | 2.954918 | 0.066 | 0.23 | 5.28E-07 | 6 | Akap7 |
| 1.83945292773501e-10 | 2.379774 | 0.063 | 0.239 | 5.52E-07 | 6 | Il11ra1 |
| 5.79718231037593e-10 | 2.404762 | 0.258 | 0.442 | 1.74E-06 | 6 | Col4a2 |
| 5.91998135318513e-10 | 4.448700 | 0.101 | 0.255 | 1.78E-06 | 6 | Fyco1 |
| 9.51945252641674e-10 | 4.532204 | 0.084 | 0.244 | 2.86E-06 | 6 | Cd48 |
| 2.02025885591625e-09 | 2.820055 | 0.146 | 0.255 | 6.06E-06 | 6 | Rcsd1 |
| 3.43593739832403e-09 | 3.197802 | 0.084 | 0.237 | 1.03E-05 | 6 | Itpkb |
| 7.00407178558117e-09 | 3.679761 | 0.105 | 0.259 | 2.10E-05 | 6 | Traf1 |
| 7.27589071989564e-09 | 3.446238 | 0.129 | 0.267 | 2.18E-05 | 6 | Ikzf1 |
| 7.66057822620417e-09 | 3.400049 | 0.118 | 0.261 | 2.30E-05 | 6 | Col23a1 |
| 9.19145943946307e-09 | 2.593605 | 0.077 | 0.248 | 2.76E-05 | 6 | Lfng |
| 9.98125547482787e-09 | 4.093310 | 0.118 | 0.27 | 2.99E-05 | 6 | Akna |
| 1.10836709360037e-08 | 3.585421 | 0.157 | 0.269 | 3.33E-05 | 6 | Ctsk |
| 1.27072413491239e-08 | 2.574335 | 0.07 | 0.233 | 3.81E-05 | 6 | Lysmd2 |
| 1.89121897199982e-08 | 2.151397 | 0.08 | 0.24 | 5.67E-05 | 6 | Cbfa2t3 |
| 2.93587864281148e-08 | 2.682496 | 0.098 | 0.251 | 8.81E-05 | 6 | Acox3 |
| 4.73780946400897e-08 | 3.530426 | 0.101 | 0.266 | 1.42E-04 | 6 | Plcg2 |
| 1.1121043815672e-07 | 2.533443 | 0.122 | 0.274 | 3.34E-04 | 6 | Phkg2 |
| 1.15222826025252e-07 | 4.726665 | 0.111 | 0.277 | 3.46E-04 | 6 | Ccdc88c |
| 1.79260929851669e-07 | 2.387085 | 0.279 | 0.291 | 5.38E-04 | 6 | Vwf |
| 2.23853603817864e-07 | 4.346494 | 0.293 | 0.465 | 6.72E-04 | 6 | Psmb4 |
| 3.78796349952862e-07 | 2.537314 | 0.066 | 0.236 | 1.14E-03 | 6 | Cbr3 |
| 4.08778187102663e-07 | 3.105068 | 0.098 | 0.282 | 1.23E-03 | 6 | Nbeal2 |
| 4.3745728402546e-07 | 2.350305 | 0.115 | 0.27 | 1.31E-03 | 6 | Fmnl1 |
| 5.69499356251778e-07 | 3.538151 | 0.206 | 0.314 | 1.71E-03 | 6 | Ptpn18 |
| 1.08326448267459e-06 | 4.032492 | 0.331 | 0.338 | 3.25E-03 | 6 | Clec2d |
| 1.17407933357689e-06 | 2.466605 | 0.066 | 0.256 | 3.52E-03 | 6 | Rbm47 |
| 1.36585438295101e-06 | 4.491135 | 0.16 | 0.297 | 4.10E-03 | 6 | Ptn |
| 1.76676598891892e-06 | 2.175430 | 0.341 | 0.47 | 5.30E-03 | 6 | Mtch1 |
| 1.97291475371125e-06 | 3.558833 | 0.084 | 0.269 | 5.92E-03 | 6 | Slc36a4 |
| 2.329172563826e-06 | 4.592004 | 0.105 | 0.286 | 6.99E-03 | 6 | Iffo1 |
| 3.21845005935607e-06 | 2.212954 | 0.111 | 0.277 | 9.66E-03 | 6 | Ctdspl |
| 3.30738780018514e-06 | 2.857341 | 0.052 | 0.253 | 9.92E-03 | 6 | Cnst |
| 3.59812367014107e-06 | 2.840571 | 0.153 | 0.298 | 1.08E-02 | 6 | Cd37 |
| 3.85790777728992e-06 | 3.848624 | 0.31 | 0.329 | 1.16E-02 | 6 | Ctsh |
| 4.44363880648269e-06 | 2.159041 | 0.105 | 0.284 | 1.33E-02 | 6 | Clock |
| 4.8314124417939e-06 | 3.922412 | 0.401 | 0.52 | 1.45E-02 | 6 | Serbp1 |
| 1.12219705302834e-05 | 4.504118 | 0.08 | 0.275 | 3.37E-02 | 6 | 1600014C10Rik |
| 1.16437189881442e-05 | 2.075647 | 0.118 | 0.292 | 3.49E-02 | 6 | Rogdi |
| 1.44585266481502e-05 | 2.299836 | 0.091 | 0.267 | 4.34E-02 | 6 | Endog |
| 1.64650461838698e-05 | 2.596225 | 0.118 | 0.281 | 4.94E-02 | 6 | Mid1ip1 |
| 1.03428888671204e-96 | 2.724919 | 0.87 | 0.234 | 3.10E-93 | 7 | Gpnmb |
| 5.7418465668481e-90 | 3.447270 | 0.888 | 0.329 | 1.72E-86 | 7 | Ctss |
| 2.67545669312894e-78 | 4.045417 | 0.884 | 0.39 | 8.03E-75 | 7 | Psap |
| 4.77854923158204e-65 | 2.258697 | 0.891 | 0.487 | 1.43E-61 | 7 | Ctsb |
| 3.83687264995177e-52 | 2.928627 | 0.849 | 0.454 | 1.15E-48 | 7 | Lgals3 |
| 2.30256070469519e-48 | 2.704282 | 0.775 | 0.369 | 6.91E-45 | 7 | Thbs2 |
| 1.34343030818799e-38 | 2.418442 | 0.737 | 0.374 | 4.03E-35 | 7 | Plin2 |
| 9.3316151720395e-14 | 3.479053 | 0.586 | 0.302 | 2.80E-10 | 7 | Nptx1 |
| 5.04698766994327e-13 | 2.007297 | 0.593 | 0.295 | 1.51E-09 | 7 | Cxcl16 |
| 6.38194391277523e-11 | 2.390111 | 0.179 | 0.166 | 1.91E-07 | 7 | Cygb |
| 5.37999361501607e-08 | 2.152426 | 0.593 | 0.367 | 1.61E-04 | 7 | Rnf149 |
| 7.54415043570361e-07 | 2.470545 | 0.182 | 0.132 | 2.26E-03 | 7 | Bmp3 |
| 1.55751828032236e-06 | 3.038649 | 0.2 | 0.136 | 4.67E-03 | 7 | Prkd1 |
| 9.60141893139688e-06 | 4.203467 | 0.235 | 0.212 | 2.88E-02 | 7 | Map3k8 |
| 2.22521626386073e-121 | 2.553892 | 0.989 | 0.403 | 6.68E-118 | 8 | Mt2 |
| 1.66361280754072e-118 | 2.477086 | 0.971 | 0.377 | 4.99E-115 | 8 | Mt1 |
| 3.97735431661695e-109 | 2.546609 | 0.996 | 0.531 | 1.19E-105 | 8 | Vim |
| 2.41725097795591e-76 | 2.021934 | 0.921 | 0.404 | 7.25E-73 | 8 | Mif |
| 6.70312183073764e-67 | 2.174273 | 0.848 | 0.347 | 2.01E-63 | 8 | Ier3 |
| 1.40160289449574e-65 | 2.223748 | 0.827 | 0.276 | 4.20E-62 | 8 | Adm |
| 7.68433137848645e-62 | 2.457509 | 0.765 | 0.193 | 2.31E-58 | 8 | Cxcl1 |
| 2.28227311796949e-54 | 2.692835 | 0.798 | 0.261 | 6.85E-51 | 8 | Ptgs2 |
| 1.50859776877922e-43 | 2.230157 | 0.769 | 0.333 | 4.53E-40 | 8 | Pdk1 |
| 1.62429866439812e-27 | 2.208635 | 0.697 | 0.366 | 4.87E-24 | 8 | Hmga2 |
| 1.37428470803553e-23 | 2.156558 | 0.686 | 0.312 | 4.12E-20 | 8 | Areg |
| 9.93226582176881e-12 | 4.317846 | 0.61 | 0.336 | 2.98E-08 | 8 | Ddit4 |
| 3.40034969149223e-129 | 5.135713 | 0.956 | 0.197 | 1.02E-125 | 9 | Cyp2f2 |
| 1.37110165808643e-106 | 5.643059 | 0.9 | 0.24 | 4.11E-103 | 9 | Cbr2 |
| 3.42996554825431e-98 | 6.072796 | 0.872 | 0.255 | 1.03E-94 | 9 | Sec14l3 |
| 1.64414874061373e-92 | 6.372693 | 0.724 | 0.117 | 4.93E-89 | 9 | Scgb3a2 |
| 1.09579306011193e-85 | 5.191929 | 0.8 | 0.218 | 3.29E-82 | 9 | Aldh1a1 |
| 6.96691990654e-85 | 8.017202 | 0.792 | 0.237 | 2.09E-81 | 9 | Wfdc2 |
| 1.82507037906325e-78 | 7.422334 | 0.604 | 0.08 | 5.48E-75 | 9 | Ccdc153 |
| 2.14779697077356e-76 | 8.775862 | 0.564 | 0.08 | 6.44E-73 | 9 | Fmo3 |
| 2.67445739707447e-75 | 7.782377 | 0.784 | 0.264 | 8.02E-72 | 9 | Tppp3 |
| 8.7226255416981e-73 | 5.640826 | 0.82 | 0.274 | 2.62E-69 | 9 | Atp1b1 |
| 1.23398996315609e-71 | 8.552515 | 0.496 | 0.065 | 3.70E-68 | 9 | Lypd2 |
| 1.11235341917205e-66 | 8.543919 | 0.56 | 0.1 | 3.34E-63 | 9 | Tmem212 |
| 2.03155894437545e-65 | 2.655969 | 0.856 | 0.276 | 6.09E-62 | 9 | Fmo2 |
| 2.32717114955753e-65 | 7.022818 | 0.492 | 0.081 | 6.98E-62 | 9 | Pon1 |
| 1.34928906466077e-63 | 7.465662 | 0.52 | 0.11 | 4.05E-60 | 9 | Cyp2s1 |
| 3.91664646931719e-62 | 6.666968 | 0.448 | 0.082 | 1.17E-58 | 9 | Gabrp |
| 1.06948107435486e-61 | 5.929897 | 0.696 | 0.244 | 3.21E-58 | 9 | Mgst1 |
| 1.43390859196376e-61 | 7.183314 | 0.496 | 0.097 | 4.30E-58 | 9 | Clic6 |
| 1.00831846678574e-60 | 2.338815 | 0.8 | 0.291 | 3.02E-57 | 9 | Gsn |
| 7.48585576566219e-60 | 8.937604 | 0.484 | 0.08 | 2.25E-56 | 9 | Fam183b |
| 1.24470472650135e-57 | 3.839145 | 0.692 | 0.199 | 3.73E-54 | 9 | Retnla |
| 1.61791530970807e-57 | 4.581096 | 0.608 | 0.206 | 4.85E-54 | 9 | Hp |
| 2.65102573795681e-57 | 7.670559 | 0.452 | 0.067 | 7.95E-54 | 9 | Gm19935 |
| 7.64999163225706e-55 | 6.182800 | 0.4 | 0.042 | 2.29E-51 | 9 | Sntn |
| 1.6365474133991e-54 | 8.763854 | 0.368 | 0.052 | 4.91E-51 | 9 | 1700007K13Rik |
| 6.33584512388178e-54 | 3.619615 | 0.6 | 0.196 | 1.90E-50 | 9 | Gsta3 |
| 1.0777821568942e-53 | 2.379876 | 0.736 | 0.27 | 3.23E-50 | 9 | Igkc |
| 1.47459788708439e-53 | 9.141435 | 0.412 | 0.061 | 4.42E-50 | 9 | Ubxn10 |
| 1.11009095367372e-52 | 5.224433 | 0.616 | 0.229 | 3.33E-49 | 9 | Spint2 |
| 1.39128912540429e-52 | 3.541987 | 0.736 | 0.297 | 4.17E-49 | 9 | Mgp |
| 6.9068702044478e-52 | 3.429632 | 0.64 | 0.22 | 2.07E-48 | 9 | Cyp4b1 |
| 4.0066707219488e-51 | 8.214175 | 0.38 | 0.032 | 1.20E-47 | 9 | Cfap44 |
| 4.53455444420349e-51 | 7.808563 | 0.38 | 0.041 | 1.36E-47 | 9 | Cfap100 |
| 1.00151772878724e-50 | 5.144974 | 0.44 | 0.141 | 3.00E-47 | 9 | Aldh1a7 |
| 5.02567788228984e-50 | 8.315417 | 0.42 | 0.062 | 1.51E-46 | 9 | Cfap43 |
| 6.66428568748788e-49 | 7.037014 | 0.376 | 0.061 | 2.00E-45 | 9 | Nme5 |
| 7.84516795229777e-49 | 4.607543 | 0.472 | 0.131 | 2.35E-45 | 9 | Ighg2c |
| 8.20485235602421e-49 | 5.031771 | 0.464 | 0.105 | 2.46E-45 | 9 | Hmgcs2 |
| 9.04300719529737e-48 | 9.362466 | 0.392 | 0.071 | 2.71E-44 | 9 | Dnah12 |
| 4.11822680371234e-47 | 7.176586 | 0.384 | 0.059 | 1.24E-43 | 9 | Slc16a11 |
| 3.6671684817323e-46 | 4.844972 | 0.488 | 0.161 | 1.10E-42 | 9 | Ces1d |
| 4.11115797401323e-46 | 5.160223 | 0.616 | 0.25 | 1.23E-42 | 9 | Selenbp1 |
| 2.09207570454316e-45 | 9.184470 | 0.336 | 0.045 | 6.28E-42 | 9 | Fhad1 |
| 5.83574780498099e-45 | 2.408022 | 0.36 | 0.056 | 1.75E-41 | 9 | Tcap |
| 2.23893371473005e-44 | 7.211091 | 0.356 | 0.056 | 6.72E-41 | 9 | Foxj1 |
| 1.6969058261286e-43 | 9.178292 | 0.328 | 0.059 | 5.09E-40 | 9 | Ttc21a |
| 3.4813014529287e-43 | 7.153806 | 0.436 | 0.131 | 1.04E-39 | 9 | Cxcl17 |
| 4.03389142250944e-43 | 5.416227 | 0.532 | 0.217 | 1.21E-39 | 9 | Cd24a |
| 4.6186891302332e-43 | 6.054014 | 0.516 | 0.203 | 1.39E-39 | 9 | Cldn3 |
| 9.31705232778342e-43 | 7.314045 | 0.372 | 0.063 | 2.80E-39 | 9 | Rsph1 |
| 1.09009685922082e-42 | 5.236319 | 0.396 | 0.092 | 3.27E-39 | 9 | Myh11 |
| 1.68471667875788e-42 | 6.870082 | 0.344 | 0.062 | 5.05E-39 | 9 | Dnah9 |
| 4.25294713016357e-42 | 3.588599 | 0.48 | 0.178 | 1.28E-38 | 9 | Ighj4 |
| 2.05889361009028e-41 | 7.149328 | 0.36 | 0.08 | 6.18E-38 | 9 | Ccdc17 |
| 3.04247489427785e-41 | 4.593176 | 0.36 | 0.099 | 9.13E-38 | 9 | 2610028H24Rik |
| 9.78181932290743e-41 | 3.352558 | 0.536 | 0.216 | 2.93E-37 | 9 | Igfbp5 |
| 1.17041433904382e-40 | 7.894139 | 0.388 | 0.103 | 3.51E-37 | 9 | Cfap126 |
| 1.26887954285607e-40 | 6.967773 | 0.344 | 0.047 | 3.81E-37 | 9 | Cdhr3 |
| 1.872704102145e-40 | 2.915903 | 0.54 | 0.189 | 5.62E-37 | 9 | Dpep1 |
| 3.24636208196513e-40 | 5.454004 | 0.396 | 0.123 | 9.74E-37 | 9 | Aox3 |
| 1.00656183482466e-39 | 2.252336 | 0.76 | 0.342 | 3.02E-36 | 9 | Txnip |
| 2.50587849278313e-39 | 8.326940 | 0.376 | 0.114 | 7.52E-36 | 9 | Ccdc162 |
| 4.75111826966164e-38 | 4.249436 | 0.524 | 0.22 | 1.43E-34 | 9 | Mettl7a1 |
| 5.04637057860787e-38 | 5.050000 | 0.372 | 0.125 | 1.51E-34 | 9 | 5330417C22Rik |
| 6.86282689389248e-38 | 8.071313 | 0.34 | 0.088 | 2.06E-34 | 9 | Tspan1 |
| 2.40361744990066e-37 | 7.754806 | 0.308 | 0.044 | 7.21E-34 | 9 | Spata18 |
| 3.85847613572811e-37 | 8.421933 | 0.376 | 0.063 | 1.16E-33 | 9 | Cfap65 |
| 2.05369839582323e-36 | 9.547011 | 0.34 | 0.066 | 6.16E-33 | 9 | Gm867 |
| 2.71279841872242e-36 | 4.746141 | 0.408 | 0.164 | 8.14E-33 | 9 | Car8 |
| 5.48265289379376e-36 | 3.169591 | 0.468 | 0.183 | 1.64E-32 | 9 | Akr1c14 |
| 1.21454015368165e-35 | 7.759675 | 0.364 | 0.117 | 3.64E-32 | 9 | Map7 |
| 7.17165002454375e-35 | 2.482896 | 0.628 | 0.259 | 2.15E-31 | 9 | Gas6 |
| 2.05102152771571e-34 | 3.868592 | 0.4 | 0.146 | 6.15E-31 | 9 | Epb41l4b |
| 3.4096991802115e-34 | 5.758732 | 0.408 | 0.118 | 1.02E-30 | 9 | Cldn10 |
| 3.8585150231731e-34 | 8.911976 | 0.34 | 0.08 | 1.16E-30 | 9 | Ccdc30 |
| 7.19026847071601e-34 | 4.891217 | 0.676 | 0.332 | 2.16E-30 | 9 | Por |
| 8.07124578244076e-34 | 3.821652 | 0.472 | 0.212 | 2.42E-30 | 9 | Myh14 |
| 1.43706067401883e-33 | 2.469105 | 0.568 | 0.238 | 4.31E-30 | 9 | C4b |
| 9.05978488158113e-33 | 3.696235 | 0.276 | 0.047 | 2.72E-29 | 9 | Hspb7 |
| 1.02276304946046e-32 | 2.353820 | 0.568 | 0.245 | 3.07E-29 | 9 | Scnn1a |
| 6.48552190714057e-32 | 7.144102 | 0.428 | 0.202 | 1.95E-28 | 9 | Entpd5 |
| 6.72268451621499e-32 | 4.888575 | 0.564 | 0.239 | 2.02E-28 | 9 | Sod3 |
| 7.28194559313181e-32 | 6.234796 | 0.588 | 0.291 | 2.18E-28 | 9 | Dcxr |
| 1.54189593303238e-31 | 2.420708 | 0.556 | 0.232 | 4.63E-28 | 9 | Ighm |
| 1.579270497871e-31 | 4.577025 | 0.472 | 0.186 | 4.74E-28 | 9 | Clec3b |
| 1.94938440351576e-31 | 4.584138 | 0.604 | 0.303 | 5.85E-28 | 9 | Appl2 |
| 2.25890095261324e-31 | 4.488563 | 0.376 | 0.141 | 6.78E-28 | 9 | Plekhb1 |
| 2.47193971430632e-31 | 2.836098 | 0.548 | 0.242 | 7.42E-28 | 9 | Serping1 |
| 1.07121292150833e-30 | 2.021716 | 0.636 | 0.285 | 3.21E-27 | 9 | Bgn |
| 2.70625057530965e-30 | 4.215523 | 0.3 | 0.109 | 8.12E-27 | 9 | Atp7b |
| 4.62665080436796e-30 | 8.148928 | 0.308 | 0.061 | 1.39E-26 | 9 | Cfap54 |
| 1.28927951979302e-29 | 7.447475 | 0.316 | 0.055 | 3.87E-26 | 9 | Ppil6 |
| 1.96175293107032e-29 | 6.119840 | 0.292 | 0.091 | 5.89E-26 | 9 | Smim22 |
| 1.28207083236387e-28 | 2.626936 | 0.628 | 0.299 | 3.85E-25 | 9 | Tmem176a |
| 1.77826818259043e-28 | 4.098541 | 0.396 | 0.186 | 5.33E-25 | 9 | Atp6v1c2 |
| 6.23468498808311e-28 | 3.474055 | 0.368 | 0.145 | 1.87E-24 | 9 | Klf15 |
| 9.71140589234936e-28 | 4.299445 | 0.34 | 0.136 | 2.91E-24 | 9 | Mamdc2 |
| 1.7112702738134e-27 | 5.248981 | 0.384 | 0.177 | 5.13E-24 | 9 | Cldn7 |
| 2.37393425743761e-27 | 4.799382 | 0.388 | 0.173 | 7.12E-24 | 9 | Scnn1b |
| 2.5365705237809e-27 | 6.847117 | 0.3 | 0.113 | 7.61E-24 | 9 | Dnaja4 |
| 9.69819818966413e-27 | 4.688288 | 0.276 | 0.091 | 2.91E-23 | 9 | Tcea3 |
| 1.11397848743205e-26 | 6.879042 | 0.288 | 0.085 | 3.34E-23 | 9 | Rsph10b |
| 1.18146315596252e-26 | 4.364503 | 0.332 | 0.179 | 3.54E-23 | 9 | Usp18 |
| 3.54699815667929e-26 | 6.340868 | 0.352 | 0.181 | 1.06E-22 | 9 | Epcam |
| 4.31384995332002e-26 | 6.293700 | 0.636 | 0.343 | 1.29E-22 | 9 | Cyb5a |
| 8.22288535780754e-26 | 6.344969 | 0.32 | 0.096 | 2.47E-22 | 9 | Dynlrb2 |
| 9.34342547284512e-26 | 6.402993 | 0.36 | 0.174 | 2.80E-22 | 9 | Krt19 |
| 1.27699185028562e-25 | 2.721847 | 0.5 | 0.234 | 3.83E-22 | 9 | Plpp3 |
| 1.54010954875301e-25 | 7.601057 | 0.328 | 0.133 | 4.62E-22 | 9 | Calml4 |
| 4.01677775336413e-25 | 5.497473 | 0.56 | 0.302 | 1.21E-21 | 9 | Gstm1 |
| 5.84360250438452e-25 | 8.100012 | 0.316 | 0.107 | 1.75E-21 | 9 | Drc3 |
| 6.77512099290288e-25 | 4.241900 | 0.404 | 0.207 | 2.03E-21 | 9 | Cdh1 |
| 6.83223862650945e-25 | 5.225762 | 0.316 | 0.153 | 2.05E-21 | 9 | 1810010H24Rik |
| 6.94084173537471e-25 | 6.996565 | 0.28 | 0.134 | 2.08E-21 | 9 | Lratd2 |
| 9.6311752829183e-25 | 2.097726 | 0.364 | 0.094 | 2.89E-21 | 9 | Tnnt2 |
| 1.10356230479046e-24 | 6.703855 | 0.3 | 0.099 | 3.31E-21 | 9 | Mlf1 |
| 1.58064907435296e-24 | 4.677512 | 0.612 | 0.317 | 4.74E-21 | 9 | Col1a2 |
| 1.78277878828157e-24 | 7.225403 | 0.252 | 0.096 | 5.35E-21 | 9 | Dmkn |
| 4.15169357028094e-24 | 4.625564 | 0.496 | 0.259 | 1.25E-20 | 9 | Ephx1 |
| 4.30715651776766e-24 | 7.669789 | 0.5 | 0.266 | 1.29E-20 | 9 | Tagln |
| 5.81778196403797e-24 | 5.695549 | 0.284 | 0.11 | 1.75E-20 | 9 | Eppk1 |
| 8.49062081002127e-24 | 2.841601 | 0.408 | 0.211 | 2.55E-20 | 9 | Apod |
| 1.46526070503613e-23 | 4.107508 | 0.5 | 0.27 | 4.40E-20 | 9 | Aldh6a1 |
| 1.56131357285928e-23 | 3.134223 | 0.424 | 0.214 | 4.68E-20 | 9 | Clic3 |
| 2.78720650067195e-23 | 2.245417 | 0.496 | 0.238 | 8.36E-20 | 9 | Nbl1 |
| 3.67279370556553e-23 | 4.679883 | 0.62 | 0.332 | 1.10E-19 | 9 | Nupr1 |
| 4.52406509826288e-23 | 6.005076 | 0.432 | 0.228 | 1.36E-19 | 9 | Gsta4 |
| 1.18080660136845e-22 | 4.484519 | 0.368 | 0.19 | 3.54E-19 | 9 | Adh1 |
| 1.37352682050044e-22 | 4.794036 | 0.312 | 0.128 | 4.12E-19 | 9 | Gipc2 |
| 1.55172845604829e-22 | 3.430497 | 0.26 | 0.111 | 4.66E-19 | 9 | Rassf9 |
| 2.01130236125908e-22 | 5.431859 | 0.392 | 0.204 | 6.03E-19 | 9 | Myl9 |
| 3.50805841755337e-22 | 6.369592 | 0.284 | 0.115 | 1.05E-18 | 9 | 4833427G06Rik |
| 5.98131668653169e-22 | 2.664805 | 0.44 | 0.236 | 1.79E-18 | 9 | Ptgis |
| 6.14147033888266e-22 | 3.432117 | 0.568 | 0.28 | 1.84E-18 | 9 | Cfh |
| 1.06157560978598e-21 | 5.784817 | 0.272 | 0.115 | 3.18E-18 | 9 | Dmd |
| 1.39240277218755e-21 | 4.070825 | 0.392 | 0.202 | 4.18E-18 | 9 | Prxl2a |
| 3.04929265508208e-21 | 2.850511 | 0.268 | 0.107 | 9.15E-18 | 9 | Ptpru |
| 3.1500809042626e-21 | 4.249270 | 0.296 | 0.138 | 9.45E-18 | 9 | Cd55 |
| 3.83857370699727e-21 | 3.658497 | 0.588 | 0.296 | 1.15E-17 | 9 | Sparcl1 |
| 4.77125420684311e-21 | 6.291939 | 0.512 | 0.303 | 1.43E-17 | 9 | Alas1 |
| 6.69111798015364e-21 | 6.811250 | 0.508 | 0.289 | 2.01E-17 | 9 | Acta2 |
| 7.7217265303386e-21 | 2.658046 | 0.34 | 0.168 | 2.32E-17 | 9 | Fgfr3 |
| 8.58737016657565e-21 | 5.522571 | 0.636 | 0.354 | 2.58E-17 | 9 | Cp |
| 1.03361394232851e-20 | 2.344501 | 0.276 | 0.128 | 3.10E-17 | 9 | Glb1l2 |
| 1.37687030082902e-20 | 5.800093 | 0.472 | 0.253 | 4.13E-17 | 9 | Des |
| 1.3915268842674e-20 | 4.555254 | 0.544 | 0.304 | 4.17E-17 | 9 | Cat |
| 2.3473666495953e-20 | 7.529937 | 0.336 | 0.19 | 7.04E-17 | 9 | Traf3ip1 |
| 2.38198410872375e-20 | 6.434450 | 0.244 | 0.119 | 7.15E-17 | 9 | Elf3 |
| 3.82389045478174e-20 | 3.540197 | 0.264 | 0.137 | 1.15E-16 | 9 | Gramd2 |
| 3.92612958174216e-20 | 6.052271 | 0.248 | 0.064 | 1.18E-16 | 9 | Pigr |
| 4.32433635949788e-20 | 4.731685 | 0.312 | 0.179 | 1.30E-16 | 9 | Lbp |
| 4.78191497753781e-20 | 4.128325 | 0.5 | 0.242 | 1.43E-16 | 9 | Ogn |
| 5.61193967228895e-20 | 3.592861 | 0.404 | 0.223 | 1.68E-16 | 9 | Gdpd1 |
| 7.31348955004661e-20 | 3.139136 | 0.256 | 0.132 | 2.19E-16 | 9 | Erbb3 |
| 9.36798935970182e-20 | 5.655136 | 0.268 | 0.117 | 2.81E-16 | 9 | Celsr1 |
| 1.95475557881734e-19 | 2.811893 | 0.228 | 0.111 | 5.86E-16 | 9 | Fam107a |
| 2.3250628485684e-19 | 4.711631 | 0.364 | 0.195 | 6.98E-16 | 9 | Acot1 |
| 2.72668821980366e-19 | 4.587236 | 0.412 | 0.237 | 8.18E-16 | 9 | Sorl1 |
| 3.01677040079683e-19 | 6.052255 | 0.3 | 0.153 | 9.05E-16 | 9 | Hhip |
| 3.56998486683992e-19 | 4.854911 | 0.304 | 0.173 | 1.07E-15 | 9 | Enpp5 |
| 4.04758537746851e-19 | 4.113441 | 0.228 | 0.123 | 1.21E-15 | 9 | Arhgef38 |
| 4.42391988398914e-19 | 3.539882 | 0.368 | 0.197 | 1.33E-15 | 9 | Camk2n1 |
| 4.79543595320958e-19 | 3.580528 | 0.364 | 0.198 | 1.44E-15 | 9 | Crispld2 |
| 2.314125205275e-18 | 7.941206 | 0.28 | 0.095 | 6.94E-15 | 9 | Anxa8 |
| 3.42214037985138e-18 | 5.265571 | 0.212 | 0.113 | 1.03E-14 | 9 | Fgfbp1 |
| 5.76715281339796e-18 | 5.451538 | 0.344 | 0.211 | 1.73E-14 | 9 | Pglyrp1 |
| 6.25561093120543e-18 | 2.994911 | 0.292 | 0.163 | 1.88E-14 | 9 | Atp9a |
| 6.68843875532737e-18 | 7.553118 | 0.324 | 0.177 | 2.01E-14 | 9 | 1700094D03Rik |
| 7.54585894164927e-18 | 2.073854 | 0.232 | 0.102 | 2.26E-14 | 9 | Kcnk3 |
| 7.87802207621203e-18 | 4.620549 | 0.26 | 0.155 | 2.36E-14 | 9 | Tst |
| 1.27760581577411e-17 | 2.763299 | 0.62 | 0.375 | 3.83E-14 | 9 | Cst3 |
| 1.37356025203312e-17 | 6.549339 | 0.428 | 0.251 | 4.12E-14 | 9 | Rgs2 |
| 1.8458282552228e-17 | 3.103263 | 0.308 | 0.182 | 5.54E-14 | 9 | Cpm |
| 2.08922024114464e-17 | 3.702393 | 0.5 | 0.305 | 6.27E-14 | 9 | Aldh3b1 |
| 1.18198052230903e-16 | 4.136386 | 0.36 | 0.206 | 3.55E-13 | 9 | Mmp2 |
| 1.49444780979955e-16 | 2.876637 | 0.24 | 0.172 | 4.48E-13 | 9 | Gpr155 |
| 1.62034485153754e-16 | 4.929959 | 0.288 | 0.168 | 4.86E-13 | 9 | Prss8 |
| 1.68974822383769e-16 | 2.225315 | 0.268 | 0.16 | 5.07E-13 | 9 | Itga8 |
| 3.1330062219222e-16 | 3.953203 | 0.264 | 0.174 | 9.40E-13 | 9 | Cxadr |
| 3.71165319112151e-16 | 6.452149 | 0.248 | 0.105 | 1.11E-12 | 9 | Actg2 |
| 3.88047192725712e-16 | 5.416424 | 0.272 | 0.159 | 1.16E-12 | 9 | Rasl12 |
| 6.69470728978372e-16 | 3.913922 | 0.256 | 0.149 | 2.01E-12 | 9 | Fam174b |
| 7.85021031134237e-16 | 3.856929 | 0.412 | 0.254 | 2.36E-12 | 9 | Dapk1 |
| 8.31888908455811e-16 | 5.299299 | 0.22 | 0.112 | 2.50E-12 | 9 | Myo5b |
| 1.10162089494076e-15 | 2.105498 | 0.368 | 0.216 | 3.30E-12 | 9 | Nkx2-1 |
| 1.28717945489012e-15 | 4.252311 | 0.52 | 0.309 | 3.86E-12 | 9 | Tmem176b |
| 1.33030695818542e-15 | 4.485941 | 0.236 | 0.128 | 3.99E-12 | 9 | Dapk2 |
| 1.60656664737574e-15 | 5.049393 | 0.252 | 0.146 | 4.82E-12 | 9 | Plxnb1 |
| 1.97079316223192e-15 | 3.521103 | 0.264 | 0.128 | 5.91E-12 | 9 | Bmp3 |
| 2.36853626429154e-15 | 5.225486 | 0.236 | 0.123 | 7.11E-12 | 9 | Eya2 |
| 2.94907630934755e-15 | 3.784121 | 0.488 | 0.308 | 8.85E-12 | 9 | Lgals3bp |
| 3.11058388547259e-15 | 2.762836 | 0.244 | 0.161 | 9.33E-12 | 9 | Slc7a2 |
| 4.98334382009778e-15 | 3.379723 | 0.244 | 0.159 | 1.50E-11 | 9 | Sox13 |
| 8.50238551825974e-15 | 2.886616 | 0.356 | 0.23 | 2.55E-11 | 9 | Depp1 |
| 9.50061279588621e-15 | 3.696489 | 0.2 | 0.098 | 2.85E-11 | 9 | Pi16 |
| 1.00595168259327e-14 | 2.826486 | 0.264 | 0.178 | 3.02E-11 | 9 | Kcnj15 |
| 1.00691983992226e-14 | 5.565102 | 0.384 | 0.257 | 3.02E-11 | 9 | Ccdc181 |
| 1.35214489411237e-14 | 2.664353 | 0.196 | 0.126 | 4.06E-11 | 9 | Il18r1 |
| 1.3562937505302e-14 | 7.328881 | 0.588 | 0.359 | 4.07E-11 | 9 | Tpm2 |
| 1.64452329671998e-14 | 4.177508 | 0.316 | 0.228 | 4.93E-11 | 9 | Tmem98 |
| 1.74704577943767e-14 | 2.947063 | 0.208 | 0.131 | 5.24E-11 | 9 | Grk3 |
| 1.90672504715618e-14 | 6.139042 | 0.268 | 0.168 | 5.72E-11 | 9 | Ttc9 |
| 2.38113049441669e-14 | 2.398135 | 0.208 | 0.123 | 7.14E-11 | 9 | Adrb1 |
| 2.38655766274347e-14 | 4.448795 | 0.372 | 0.217 | 7.16E-11 | 9 | Cbr3 |
| 2.49816469832432e-14 | 3.670781 | 0.304 | 0.19 | 7.49E-11 | 9 | Prr15l |
| 4.54963161872503e-14 | 3.898739 | 0.284 | 0.177 | 1.36E-10 | 9 | Gstt2 |
| 6.40804329373749e-14 | 3.016983 | 0.288 | 0.203 | 1.92E-10 | 9 | Rnf128 |
| 6.95215990069666e-14 | 3.773702 | 0.256 | 0.191 | 2.09E-10 | 9 | Stim1 |
| 7.65901471595756e-14 | 2.750511 | 0.256 | 0.158 | 2.30E-10 | 9 | Slc46a3 |
| 1.25444070059835e-13 | 5.643154 | 0.536 | 0.327 | 3.76E-10 | 9 | Tsc22d1 |
| 1.45100275877557e-13 | 3.744834 | 0.232 | 0.15 | 4.35E-10 | 9 | Ptpn13 |
| 1.50787855975146e-13 | 3.333604 | 0.272 | 0.188 | 4.52E-10 | 9 | Epn3 |
| 1.5305056085173e-13 | 6.604117 | 0.308 | 0.212 | 4.59E-10 | 9 | Arhgdig |
| 1.91872937523402e-13 | 6.570632 | 0.28 | 0.183 | 5.76E-10 | 9 | Spint1 |
| 5.22945020447615e-13 | 6.551972 | 0.26 | 0.18 | 1.57E-09 | 9 | Ckb |
| 7.26870431310912e-13 | 2.190278 | 0.252 | 0.174 | 2.18E-09 | 9 | Plxdc2 |
| 7.55886939845476e-13 | 2.645733 | 0.244 | 0.164 | 2.27E-09 | 9 | Rab15 |
| 8.07946712762991e-13 | 3.037959 | 0.316 | 0.193 | 2.42E-09 | 9 | Hspb1 |
| 1.01208715826598e-12 | 3.497091 | 0.296 | 0.19 | 3.04E-09 | 9 | Fxyd1 |
| 1.19409126757142e-12 | 3.943138 | 0.496 | 0.31 | 3.58E-09 | 9 | Trf |
| 1.92173774053309e-12 | 3.137681 | 0.208 | 0.144 | 5.77E-09 | 9 | Nckap5 |
| 1.94038361442476e-12 | 4.135743 | 0.384 | 0.212 | 5.82E-09 | 9 | Galnt15 |
| 2.16021697001958e-12 | 4.198454 | 0.248 | 0.136 | 6.48E-09 | 9 | Smim6 |
| 2.38099994530436e-12 | 5.565812 | 0.296 | 0.227 | 7.14E-09 | 9 | Hes6 |
| 2.53427410523818e-12 | 4.003542 | 0.244 | 0.153 | 7.60E-09 | 9 | AU021092 |
| 2.77740360521718e-12 | 2.960155 | 0.256 | 0.157 | 8.33E-09 | 9 | Dkk3 |
| 3.45569055164742e-12 | 2.684909 | 0.376 | 0.219 | 1.04E-08 | 9 | Gpc3 |
| 4.30978662679965e-12 | 2.547857 | 0.336 | 0.205 | 1.29E-08 | 9 | Sult1a1 |
| 5.44896127093543e-12 | 2.598475 | 0.248 | 0.149 | 1.63E-08 | 9 | Klhdc8b |
| 5.97486710191832e-12 | 2.519099 | 0.552 | 0.316 | 1.79E-08 | 9 | Ucp2 |
| 6.04608031284054e-12 | 3.062250 | 0.356 | 0.251 | 1.81E-08 | 9 | Nr1d1 |
| 7.32537536224742e-12 | 2.891895 | 0.208 | 0.124 | 2.20E-08 | 9 | Podn |
| 7.54401774246663e-12 | 5.500650 | 0.212 | 0.148 | 2.26E-08 | 9 | Kbtbd11 |
| 7.5776198037545e-12 | 6.105466 | 0.244 | 0.138 | 2.27E-08 | 9 | Myo5c |
| 8.6067435940889e-12 | 4.239851 | 0.576 | 0.352 | 2.58E-08 | 9 | Igfbp7 |
| 9.46002943328394e-12 | 2.878929 | 0.3 | 0.173 | 2.84E-08 | 9 | Cpxm1 |
| 1.06226534913979e-11 | 4.345909 | 0.244 | 0.167 | 3.19E-08 | 9 | Lmcd1 |
| 1.16948530909904e-11 | 4.027416 | 0.244 | 0.146 | 3.51E-08 | 9 | Ppl |
| 1.31455509482043e-11 | 3.516538 | 0.248 | 0.172 | 3.94E-08 | 9 | Clip4 |
| 1.7881021488944e-11 | 3.946856 | 0.336 | 0.226 | 5.36E-08 | 9 | Mylk |
| 2.10636323056886e-11 | 2.446513 | 0.436 | 0.283 | 6.32E-08 | 9 | Trp53inp1 |
| 2.39009383481272e-11 | 2.707979 | 0.212 | 0.154 | 7.17E-08 | 9 | Nox4 |
| 2.63413676123125e-11 | 2.639359 | 0.28 | 0.19 | 7.90E-08 | 9 | Kalrn |
| 2.69807546690292e-11 | 2.028308 | 0.284 | 0.209 | 8.09E-08 | 9 | Tspan13 |
| 4.22266489172256e-11 | 3.123310 | 0.288 | 0.18 | 1.27E-07 | 9 | Per2 |
| 8.03461712119183e-11 | 4.529659 | 0.196 | 0.139 | 2.41E-07 | 9 | Mageh1 |
| 9.65493939492888e-11 | 2.874226 | 0.264 | 0.19 | 2.90E-07 | 9 | Smtnl2 |
| 1.07098462208277e-10 | 6.320502 | 0.384 | 0.282 | 3.21E-07 | 9 | Slc23a2 |
| 1.20099853679931e-10 | 3.031732 | 0.304 | 0.206 | 3.60E-07 | 9 | Nebl |
| 1.30504379567372e-10 | 2.475211 | 0.38 | 0.264 | 3.92E-07 | 9 | Cystm1 |
| 1.53875477909238e-10 | 5.135079 | 0.22 | 0.147 | 4.62E-07 | 9 | Tiam1 |
| 1.80973070725624e-10 | 2.564397 | 0.316 | 0.225 | 5.43E-07 | 9 | Ccdc28b |
| 2.01990466190061e-10 | 2.871043 | 0.212 | 0.135 | 6.06E-07 | 9 | Lama2 |
| 2.48569059803018e-10 | 2.827226 | 0.252 | 0.175 | 7.46E-07 | 9 | Bicc1 |
| 2.62281546430081e-10 | 2.477992 | 0.248 | 0.187 | 7.87E-07 | 9 | Rora |
| 3.17868601496005e-10 | 3.373486 | 0.448 | 0.337 | 9.54E-07 | 9 | Elof1 |
| 3.18922228647479e-10 | 3.352083 | 0.272 | 0.214 | 9.57E-07 | 9 | Slit3 |
| 3.47546025158543e-10 | 3.541656 | 0.336 | 0.252 | 1.04E-06 | 9 | Fgl2 |
| 3.50950732084884e-10 | 2.505484 | 0.196 | 0.147 | 1.05E-06 | 9 | Slc24a4 |
| 3.61872820366365e-10 | 2.981678 | 0.236 | 0.17 | 1.09E-06 | 9 | Mtus1 |
| 3.82074952374084e-10 | 2.985613 | 0.144 | 0.129 | 1.15E-06 | 9 | Clca3a1 |
| 5.37652299393767e-10 | 3.545945 | 0.496 | 0.324 | 1.61E-06 | 9 | Gclc |
| 6.42575648015115e-10 | 4.873834 | 0.212 | 0.157 | 1.93E-06 | 9 | E330009J07Rik |
| 7.43024161621122e-10 | 2.763162 | 0.356 | 0.25 | 2.23E-06 | 9 | Ptprf |
| 8.00131048114356e-10 | 3.577388 | 0.312 | 0.221 | 2.40E-06 | 9 | Plet1 |
| 1.01020562750313e-09 | 4.002100 | 0.192 | 0.368 | 3.03E-06 | 9 | Uchl5 |
| 1.44995529901023e-09 | 3.782475 | 0.228 | 0.162 | 4.35E-06 | 9 | Dtna |
| 1.71839795567226e-09 | 2.437085 | 0.232 | 0.184 | 5.16E-06 | 9 | Enpep |
| 2.54702677658574e-09 | 2.729988 | 0.348 | 0.238 | 7.64E-06 | 9 | Serpina3n |
| 2.91230713648801e-09 | 2.038712 | 0.408 | 0.274 | 8.74E-06 | 9 | Dram1 |
| 3.01120272932861e-09 | 4.755842 | 0.392 | 0.299 | 9.03E-06 | 9 | Slc25a10 |
| 4.39872734594007e-09 | 4.040286 | 0.296 | 0.226 | 1.32E-05 | 9 | Ramp1 |
| 4.45291021787623e-09 | 6.905167 | 0.58 | 0.364 | 1.34E-05 | 9 | Csrp1 |
| 5.66434852459721e-09 | 4.688999 | 0.34 | 0.251 | 1.70E-05 | 9 | Endog |
| 5.67431623150272e-09 | 4.551458 | 0.28 | 0.231 | 1.70E-05 | 9 | Lrba |
| 5.7546751372543e-09 | 3.000471 | 0.244 | 0.189 | 1.73E-05 | 9 | Rbms3 |
| 5.755517741731e-09 | 5.494333 | 0.24 | 0.148 | 1.73E-05 | 9 | Abca8a |
| 6.91972604152224e-09 | 4.307802 | 0.296 | 0.259 | 2.08E-05 | 9 | Ift172 |
| 7.03048859060968e-09 | 4.316252 | 0.172 | 0.113 | 2.11E-05 | 9 | Aff3 |
| 8.06821044103898e-09 | 2.745606 | 0.208 | 0.157 | 2.42E-05 | 9 | Slc6a14 |
| 8.92176535045022e-09 | 3.449714 | 0.196 | 0.179 | 2.68E-05 | 9 | Boc |
| 1.09048470458783e-08 | 4.271247 | 0.26 | 0.2 | 3.27E-05 | 9 | Baiap2l1 |
| 1.32509737240293e-08 | 2.681419 | 0.164 | 0.133 | 3.98E-05 | 9 | Pde9a |
| 1.4825754323996e-08 | 3.634176 | 0.228 | 0.163 | 4.45E-05 | 9 | Cygb |
| 1.56152501596742e-08 | 2.939561 | 0.176 | 0.1 | 4.68E-05 | 9 | Synm |
| 1.74568497677839e-08 | 2.660681 | 0.256 | 0.223 | 5.24E-05 | 9 | Chpt1 |
| 1.79288715010138e-08 | 2.130968 | 0.272 | 0.224 | 5.38E-05 | 9 | Pcx |
| 1.86972730976894e-08 | 3.072034 | 0.18 | 0.161 | 5.61E-05 | 9 | Bex2 |
| 1.92099741956249e-08 | 2.332865 | 0.268 | 0.2 | 5.76E-05 | 9 | Efnb1 |
| 2.35366040299295e-08 | 2.578313 | 0.156 | 0.119 | 7.06E-05 | 9 | Rasgef1a |
| 2.55431369553355e-08 | 2.483424 | 0.264 | 0.206 | 7.66E-05 | 9 | Ism1 |
| 2.9135240542842e-08 | 2.197875 | 0.54 | 0.376 | 8.74E-05 | 9 | S100a1 |
| 3.06813362587051e-08 | 2.379059 | 0.264 | 0.199 | 9.20E-05 | 9 | Lgi3 |
| 3.10692823695907e-08 | 4.436167 | 0.252 | 0.186 | 9.32E-05 | 9 | Svep1 |
| 3.56138607542259e-08 | 3.758013 | 0.332 | 0.252 | 1.07E-04 | 9 | Tmem50b |
| 3.90689951618305e-08 | 3.171129 | 0.292 | 0.244 | 1.17E-04 | 9 | Retreg1 |
| 4.03956306322384e-08 | 4.980180 | 0.22 | 0.157 | 1.21E-04 | 9 | Scmh1 |
| 4.06256296389679e-08 | 3.028755 | 0.184 | 0.145 | 1.22E-04 | 9 | Ptpn3 |
| 4.06312554733918e-08 | 3.288383 | 0.248 | 0.197 | 1.22E-04 | 9 | Dmpk |
| 4.93014699893076e-08 | 3.553259 | 0.188 | 0.168 | 1.48E-04 | 9 | Pcdhgb4 |
| 5.73017082554884e-08 | 2.076375 | 0.42 | 0.288 | 1.72E-04 | 9 | H2-Eb1 |
| 6.72287044185962e-08 | 2.438474 | 0.552 | 0.359 | 2.02E-04 | 9 | Tmsb4x |
| 6.85797992506233e-08 | 3.688920 | 0.192 | 0.159 | 2.06E-04 | 9 | Ltb |
| 8.33500963385713e-08 | 3.608900 | 0.304 | 0.254 | 2.50E-04 | 9 | Epb41l5 |
| 8.39503716020844e-08 | 3.240178 | 0.2 | 0.17 | 2.52E-04 | 9 | Slc15a2 |
| 9.06643332413864e-08 | 2.054272 | 0.308 | 0.246 | 2.72E-04 | 9 | Traf1 |
| 9.73444135319772e-08 | 2.771850 | 0.184 | 0.155 | 2.92E-04 | 9 | Lsr |
| 1.16359508468341e-07 | 4.360706 | 0.256 | 0.226 | 3.49E-04 | 9 | Il11ra1 |
| 1.23632711650557e-07 | 2.042999 | 0.172 | 0.362 | 3.71E-04 | 9 | Rnf125 |
| 1.6891620282566e-07 | 3.059901 | 0.24 | 0.192 | 5.07E-04 | 9 | Phactr2 |
| 1.74088394830317e-07 | 3.515296 | 0.252 | 0.203 | 5.22E-04 | 9 | Enpp4 |
| 2.06146661735279e-07 | 5.335576 | 0.272 | 0.217 | 6.18E-04 | 9 | Mgat3 |
| 2.08353316126649e-07 | 3.624782 | 0.4 | 0.335 | 6.25E-04 | 9 | Enah |
| 2.23599901855696e-07 | 4.271213 | 0.352 | 0.282 | 6.71E-04 | 9 | Tanc2 |
| 2.54054517959697e-07 | 2.979120 | 0.288 | 0.242 | 7.62E-04 | 9 | Rbm47 |
| 2.59855300752659e-07 | 2.211109 | 0.356 | 0.262 | 7.80E-04 | 9 | Cryab |
| 2.77601653479094e-07 | 2.703282 | 0.408 | 0.294 | 8.33E-04 | 9 | Herpud1 |
| 2.83514800122956e-07 | 3.620825 | 0.288 | 0.209 | 8.51E-04 | 9 | Rarres2 |
| 3.28660681429308e-07 | 5.420647 | 0.204 | 0.372 | 9.86E-04 | 9 | Mcm2 |
| 3.63641170915397e-07 | 3.497540 | 0.212 | 0.205 | 1.09E-03 | 9 | Grb14 |
| 4.06356802253129e-07 | 2.669475 | 0.316 | 0.259 | 1.22E-03 | 9 | Ctsk |
| 4.20094400651448e-07 | 5.663325 | 0.28 | 0.222 | 1.26E-03 | 9 | Acss2 |
| 5.11705601845914e-07 | 2.012013 | 0.484 | 0.312 | 1.54E-03 | 9 | Tsc22d3 |
| 5.94701801890481e-07 | 2.509221 | 0.208 | 0.166 | 1.78E-03 | 9 | Ttll7 |
| 6.43480798879779e-07 | 3.248066 | 0.192 | 0.158 | 1.93E-03 | 9 | Pde3a |
| 6.95665981471467e-07 | 3.933631 | 0.244 | 0.211 | 2.09E-03 | 9 | Tbc1d2b |
| 8.14269362639029e-07 | 3.062253 | 0.264 | 0.234 | 2.44E-03 | 9 | Muc1 |
| 8.62625593937552e-07 | 4.028386 | 0.172 | 0.153 | 2.59E-03 | 9 | Tmem38b |
| 9.75748647982769e-07 | 3.592620 | 0.112 | 0.115 | 2.93E-03 | 9 | Scel |
| 9.8833214169542e-07 | 3.477421 | 0.308 | 0.253 | 2.96E-03 | 9 | Zfp467 |
| 1.03060426282476e-06 | 2.736825 | 0.292 | 0.26 | 3.09E-03 | 9 | Kif21a |
| 1.1125501953731e-06 | 2.631456 | 0.308 | 0.278 | 3.34E-03 | 9 | Dnajb2 |
| 1.27272032154173e-06 | 2.697448 | 0.2 | 0.194 | 3.82E-03 | 9 | Cped1 |
| 1.41552979973604e-06 | 2.803529 | 0.296 | 0.249 | 4.25E-03 | 9 | Col23a1 |
| 1.48502774482341e-06 | 2.349825 | 0.416 | 0.321 | 4.46E-03 | 9 | Pbx1 |
| 1.57560730324031e-06 | 3.102177 | 0.24 | 0.214 | 4.73E-03 | 9 | Nt5e |
| 1.68088440002692e-06 | 4.830041 | 0.232 | 0.189 | 5.04E-03 | 9 | Sort1 |
| 1.7468364654893e-06 | 3.804814 | 0.272 | 0.201 | 5.24E-03 | 9 | Mmp15 |
| 1.95662924427681e-06 | 4.842402 | 0.288 | 0.247 | 5.87E-03 | 9 | Aox1 |
| 2.17707872074223e-06 | 2.312100 | 0.32 | 0.254 | 6.53E-03 | 9 | Fgd5 |
| 2.57809404461882e-06 | 2.429038 | 0.428 | 0.305 | 7.73E-03 | 9 | Creg1 |
| 2.95263525900217e-06 | 4.862699 | 0.272 | 0.255 | 8.86E-03 | 9 | Il13ra1 |
| 3.38308596783691e-06 | 3.059757 | 0.364 | 0.276 | 1.01E-02 | 9 | Gprc5a |
| 3.75461601703056e-06 | 4.122937 | 0.324 | 0.285 | 1.13E-02 | 9 | Bphl |
| 3.88067271870045e-06 | 2.830308 | 0.168 | 0.147 | 1.16E-02 | 9 | Ackr2 |
| 4.24460302958665e-06 | 2.819364 | 0.336 | 0.408 | 1.27E-02 | 9 | Ccnd1 |
| 4.88198233114373e-06 | 2.405066 | 0.232 | 0.206 | 1.46E-02 | 9 | Tmem44 |
| 4.99141568913988e-06 | 2.194910 | 0.204 | 0.187 | 1.50E-02 | 9 | Tbx4 |
| 5.08835227753903e-06 | 5.475245 | 0.364 | 0.314 | 1.53E-02 | 9 | Basp1 |
| 5.9225130679203e-06 | 4.297838 | 0.328 | 0.28 | 1.78E-02 | 9 | Gm13889 |
| 6.35814685553016e-06 | 5.328638 | 0.288 | 0.389 | 1.91E-02 | 9 | Brix1 |
| 6.8353447549414e-06 | 2.264183 | 0.156 | 0.134 | 2.05E-02 | 9 | Tcf21 |
| 8.96876760836105e-06 | 3.358538 | 0.196 | 0.169 | 2.69E-02 | 9 | Vgll3 |
| 9.3357016460435e-06 | 3.416036 | 0.172 | 0.18 | 2.80E-02 | 9 | Fgf2 |
| 1.0329078910456e-05 | 2.577711 | 0.2 | 0.187 | 3.10E-02 | 9 | Vsig2 |
| 1.07150593991508e-05 | 2.442579 | 0.264 | 0.237 | 3.21E-02 | 9 | Ifi27l2a |
| 1.36862351445438e-05 | 2.343669 | 0.368 | 0.297 | 4.11E-02 | 9 | Ndrg2 |
| 1.45330531742324e-05 | 3.100985 | 0.164 | 0.157 | 4.36E-02 | 9 | Taf9b |
| 1.52104196516327e-05 | 2.058510 | 0.272 | 0.234 | 4.56E-02 | 9 | Rab11fip1 |
| 1.52323818307707e-05 | 3.311458 | 0.324 | 0.278 | 4.57E-02 | 9 | Cebpa |
| 1.55172150959401e-05 | 2.015079 | 0.388 | 0.284 | 4.66E-02 | 9 | Tcn2 |
| 1.59690268352692e-05 | 2.663639 | 0.212 | 0.178 | 4.79E-02 | 9 | Cd163 |
| 1.63861749936862e-05 | 4.744292 | 0.232 | 0.227 | 4.92E-02 | 9 | Eps8l2 |
