# Supplementary material for: Efineptakin alfa (NT-I7) improves overall survival and induces immune niches in murine tumors
Source: Genes Dis. 2026 Mar 10;14(1):102136. doi: 10.1016/j.gendis.2026.102136 (PMC13583571; doi:10.1016/j.gendis.2026.102136)
Supplement: Supplementary file 3 [file mmc2.docx]

SUPPLEMENTARY FIGURE LEGEND

**Supplementary figure 1. Effect of NT-I7 treatment on lymphoid organs, bone marrow, and blood.** (A) Representative spleens from control and NT-I7-treated mice. (B) Summary graph of spleen weights from control and NT-I7-treated mice (n=16 control and n=18 NT-I7). (C-G) Representative FACS plots and summary graphs of total cell counts of various immune cell types from non-draining lymph nodes (inguinal). (H-I) Summary graphs of total cell count of immune cell types in the spleen (H) and bone marrow (I). (J) Summary graphs of proportion of immune cell types in the blood. N=4 mice per group. P-values are of unpaired t test or Manny Whitney test based on normality and lognormality tests. Tissues were harvested on day 10 after NT-I7 treatment.

**Supplementary figure 2. Localization of stem-like CD8 T cells in the TME.** (A) Representative image with immune aggregate areas and remaining tissue areas annotated in NT-I7-treated tumors from Fig. 2E. (B) Ratio of stem-like CD8 and of other CD8 T cells in immune aggregates to no aggregate areas (C) and the ratio of stem-like CD8 to other CD8 T cells in immune aggregates and in areas with no aggregates (n=2).

**Supplementary Figure 3. Lymphoid structures in lymph node and intraperitoneal CT26 tumors.** (A) Representative image of a conventional secondary lymphoid organ (peripheral lymph node) with classically organized T-cell/ B-cell compartments. Scale bar: 50 μm. (B) Left panel: representative image of i.p. tumor from control tumor. Right panel: representative images of individual i.p. tumors from the NT-I7-treated group. Scale bar: 50 μm. n = 1 for the control group and n = 3 for the NT-I7 treatment group. i.p. intraperitoneal.
